# Preparation of Thermally and Photochemically Immobilized N‐type Conjugated Polymer Films via Quantitative Backbone Editing

**DOI:** 10.1002/anie.202505608

**Published:** 2025-04-10

**Authors:** Charlotte Rapley, Adam V. Marsh, Edgar Gutierrez‐Fernandez, Mohamad Insan Nugraha, Flurin Eisner, Martina Rimmele, Jaime Martín, Thomas D. Anthopoulos, Martin Heeney

**Affiliations:** ^1^ Department of Chemistry and Centre for Processable Electronics Imperial College London White City Campus London W12 0BZ UK; ^2^ Department of Physical Science and Engineering King Abdullah University of Science & Technology (KAUST) Thuwal 23955–6900 Kingdom of Saudi Arabia; ^3^ POLYMAT University of the Basque Country UPV/EHU Avenida de Tolosa 72 Donostia‐San Sebastián 20018 Spain; ^4^ Research Center for Nanotechnology Systems National Research and Innovation Agency (BRIN) South Tangerang Banten 15314 Indonesia; ^5^ School of Engineering and Materials Science Queen Mary University of London London E1 4NS UK; ^6^ Universidade da Coruña Campus Industrial de Ferrol CITENI Ferrol Esteiro 15403 Spain; ^7^ Henry Royce Institute and Photon Science Institute Department of Electrical and Electronic Engineering The University of Manchester Manchester M13 9PL UK

**Keywords:** Backbone editing, Conjugated polymer, Cross‐linking, N‐type material, Postpolymerization functionalization

## Abstract

We report a series of n‐type conjugated polymers based on PNDI‐TfBTT and PNDIV‐TfBTT backbones constructed from electron‐deficient naphthalene diimide (NDI) and fluorinated benzothiadiazole (fBT) units, with PNDIV‐TfBTT incorporating a vinylene spacer. Quantitative postpolymerization modification (PPM) via nucleophilic substitution replaced the fBT fluorine with thioether side chains, optionally containing azide groups. Thioether substitution improved solubility, while subtly changing the ordering of polymer films. Azide incorporation enabled both thermal and photochemical crosslinking, yielding insoluble and immobile films that retained good electron transport; although UV crosslinking initially reduced mobility, subsequent thermal annealing largely restored crystallinity and performance. This work underscores the utility of precise backbone editing to fine‐tune the electronic and morphological properties of n‐type polymers, offering new avenues for the fabrication of stable, patterned active layers in advanced organic electronic devices.

## Graphical abstract

A postpolymerization modification (PPM) of n‐type conjugated polymers by nucleophilic aromatic substitution is reported, enabling precise tuning of solubility, ordering, and stability. The method allows the facile introduction of azide groups, which can undergo UV‐ or thermally induced crosslinking, to afford stable, insoluble films with retained electron transport in transistor devices.

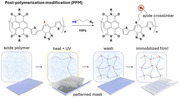

## Introduction

The electrical characteristics of organic conjugated polymers (CPs) are continually being harnessed across various electronic devices such as organic light‐emitting diodes (OLEDs),^[^ ^1^, ^2^, ^3^, ^4^ ^]^ organic photovoltaic (OPV) cells,^[^ ^5^ ^]^ organic electrochemical transistors (OECTs),^[^ ^6^, ^7^ ^]^ and particularly pertinent to this study, organic thin‐film transistors (OTFTs).^[^ ^8^, ^9^ ^]^ OTFTs consist of a dielectric film, three electrodes (source (S), drain (D), and gate (G)), and an organic semiconducting layer characterized as p‐type, n‐type, or ambipolar, depending on the predominant charge carriers of holes, electrons, or both, respectively.^[^ ^10^ ^]^ Historically, p‐type donor CPs have been more prevalent in research due to the difficulties in synthesizing stable n‐type CPs with low LUMOs for electron conduction.^[^ ^11^, ^12^ ^]^ However, recent efforts have resulted in advanced n‐type CPs possessing solubility, processability, and thermal stability properties comparable to their p‐type counterparts.^[^ ^13^, ^14^, ^15^, ^16^ ^]^

Naphthalene diimide (NDI), an electron‐deficient building block containing a conjugated naphthalene core and electron‐withdrawing diimide groups, has been widely investigated for use in n‐type CPs.^[^ ^17^, ^18^, ^19^, ^20^ ^]^ For example, conjugated polymers containing NDI as a comonomer have demonstrated some of the highest electron mobilities reported in OTFT devices.^[^ ^21^, ^22^, ^23^, ^24^ ^]^ In addition to the choice of comonomer, the nature of the solubilizing chain has been shown to have an important impact on device performance. The side chains help solubilize the polymer, and importantly promote solid‐state aggregation and self‐assembly, but also influence polymer energetics.^[^ ^22^, ^25^ ^]^ Usually, the side chains are incorporated by functionalization of the monomer prior to polymerization. Screening different side chains can therefore be time consuming, since new monomer synthesis is required for each variation. Moreover, changes in molecular weight and dispersity can occur for each different (co)polymerization, potentially complicating structure‐property investigation. The addition of reactive side chains can also present challenges, as such groups may not survive the polymerization conditions. Postpolymerization modification (PPM), though less explored, presents another viable method for introducing functional or solubilizing side chains or functionalized pendant groups to conjugated polymers.^[^ ^26^, ^27^, ^28^, ^29^ ^]^ Direct PPM onto the conjugated chain has a crucial advantage over monomer modification in that the final polymer products are comparable with respect to chain length and dispersity (*Ð*), whereas this is not necessarily the case for (co)polymerizations of different monomer materials. Although click reactions typically used in PPM have enabled the synthesis of an extensive library of new polymer materials, they are typically not performed directly on the conjugated backbone, and therefore have limited electronic impact. They can have also limitations such as a lack of selectivity or incomplete/partial substitution,^[^ ^30^, ^31^ ^]^ and these challenges have led to the exploration of alternative editing methods.

Previous research carried out by Creamer et al. focused on using nucleophilic aromatic substitution (S_N_Ar) as a PPM method to modify, or backbone edit, the p‐type polymer poly(9,9′‐dioctylfluorene‐5‐fluoro‐2,1,3‐benzothiadiazole) (F8fBT). The fluoride on 5‐fluorobenzo‐2,1,3‐thiadiazole (fBT) was displaced quantitatively via S_N_Ar with a range of functionalized thiols, thioacetates, and alcohols.^[^ ^26^ ^]^ Further reports have developed S_N_Ar methods for PPM on other p‐type CPs to tune electronic properties, modify the planarity of the backbone, manipulate physical properties (e.g., solubility), and introduce new functionality.^[^ ^32^, ^33^, ^34^, ^35^, ^36^, ^37^, ^38^, ^39^ ^]^ However, PPM implementation of such techniques on n‐type CPs has yet to be achieved.

In this study, we designed and synthesized new CPs from two fundamental building blocks: A strong electron‐withdrawing NDI repeat unit and a thiophene‐flanked fBT repeat unit (also electron withdrawing), with (PNDIV‐TfBTT) and without (PNDI‐TfBTT) a vinylene spacer. The vinylene spacer has previously been shown to help alleviate torsional twisting between the NDI and the adjacent thiophene.^[^ ^40^, ^41^ ^]^ The resulting polymers showed deep lowest unoccupied molecular orbitals (LUMOs, −4.07 and −3.94 eV for PNDI‐TfBTT and PNDIV‐TfBTT, respectively) that enable electron injection, and promote good transistor performance, especially for the highly ordered films of PNDI‐TfBTT (electron mobility of 1.4 × 10^−1^ cm^2^ V^−1^ s^−1^). As a proof of concept, quantitative PPM of these n‐type polymers by S_N_Ar from 20 to 100 mol% was demonstrated with 1‐octanethiol, achieving enhanced solubility while slightly increasing the highest occupied molecular orbital (HOMO) and LUMO energy levels due to increased backbone twisting. Additional PPM studies incorporated a side chain with a photoreactive azide group directly on PNDI‐TfBTT, yielding PNDI‐T(SAz)BTT, which enabled up to a tenfold electron mobility increase after thermal and UV crosslinking treatment, due to improved morphological order. Moreover, the presence of high levels of crosslinking azide groups facilitated polymer photopatterning and immobilization on glass substrates, exhibiting resistance to good solvents like chlorobenzene while maintaining electron transport properties. Beyond expanding the scope of stable n‐type CPs, our work highlights the effectiveness of PPM in introducing new functionality to n‐type polymers and demonstrates the potential of utilizing thermally and photochemically active backbones for patterned active layers in multilayered electronic devices.

### Results and Discussion

**Scheme 1:**
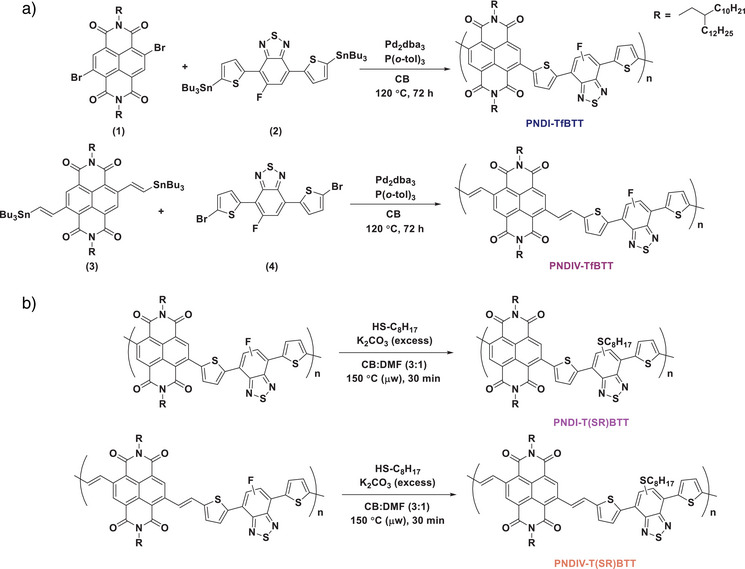
a) Synthesis of n‐type polymers PNDI‐TfBTT and PNDIV‐TfBTT backbone polymers, and b) the synthesis of PNDI‐T(SR)BTT and PNDIV‐T(SR)BTT by PPM of the n‐type parent polymers using 1‐octanethiol.

Polymers PNDI‐TfBTT and PNDIV‐TfBTT were synthesized using palladium‐catalyzed Stille polymerization, outlined in Scheme 1 (details in the Supporting Information). Stoichiometric amounts of **1** and commercially available 5‐fluoro‐4,7‐*bis*(5‐(trimethylstannyl)thiophen‐2‐yl)benzo[c][1,2,5]thiadiazole (**2**) were reacted in the presence of a palladium(0) catalyst and phosphine ligand at 120 °C in chlorobenzene in a sealed vial for three days. The mixture was precipitated into methanol, filtered, and purified by Soxhlet extraction to yield PNDI‐TfBTT as chlorobenzene‐extracted and unextracted (i.e., not soluble in hot chlorobenzene) material fractions in a combined 86% yield. Using the same procedure, stannylated NDI **3** and commercially available 4,7‐dibromo‐5‐fluoro‐2,1,3‐benzothiadiazole (**4**) were reacted and purified to produce PNDIV‐TfBTT in chlorobenzene and unextracted material fractions in a combined 82% yield.

Because of the increased planarity of the polymer relative to PNDI‐TfBTT, enabled by the vinylene spacer, PNDIV‐TfBTT was found to strongly aggregate, resulting in reduced NMR signal intensities (^1^H and ^19^F NMR spectra shown in Figures S1–S4; similar aggregation effects were previously reported for conjugated copolymers containing vinylene‐flanked NDI repeat units^[^ ^40^, ^41^ ^]^). The soluble fractions of the polymers were subjected to analytical gel‐permeation chromatography (GPC), where the number average molecule weight (M_n_), weight average molecular weight (M_w_), and dispersity (Đ) values were measured. PNDI‐TfBTT had GPC values of *M*_n_ = 26 kDa and *Đ* = 3.7, while PNDIV‐TfBTT had values of *M*_n_ = 43 kDa and *Đ* = 2.1 versus polystyrene standards (Table S1 and Figure S5). For PNDI‐TfBTT, the more soluble polymer material extracted from chlorobenzene was used for the partial substitution studies outlined later, while for both PNDI‐TfBTT and PNDIV‐TfBTT, the unextracted polymer material was used for complete substitution reaction studies. It is noteworthy that this allows the conversion of a poorly processable, essentially waste, polymer into a useful material. Partial substitution as the reaction proceeds helps to fully solubilize the polymer, enabling full displacement.

### PPM with Thioalkyl Substituents via S_N_Ar

Before embarking on PPM of the polymers, we investigated the stability of 2,7‐*bis*(2‐decyltetradecyl)benzo[*lmn*][3,8]phenanthroline‐1,3,6,8 (2H,7H)‐tetraone (NDI) against a series of bases under S_N_Ar conditions except with no nucleophile present, and found K_2_CO_3_ to be the most suitable with no degradation of the NDI core up to 150 °C (Figure S6).

To examine the feasibility and effects of PPM on the n‐type polymers PNDI‐TfBTT and PNDIV‐TfBTT, 1‐octanethiol was chosen for the backbone substitution given the good nucleophilicity of thiols in S_N_Ar fluoride displacement reactions and the likely solubilizing effect of the resulting octyl thioether. Accordingly, the insoluble polymers were reacted with excess 1‐octanethiol in the presence of K_2_CO_3_ in a chlorobenzene/DMF (3/1 v/v) solvent mixture at 150 °C for 30 min in a microwave reactor (Scheme 1), obtaining PNDI‐T(SR)BTT and PNDIV‐T(SR)BTT, in yields of 62% and 48%, respectively (^1^H and ^19^F NMR characterization data shown in Figures S7–S10). This temperature was chosen to ensure some solubility of the polymers in the reaction solvent. The absence of any discernible peaks in the ^19^F NMR spectra of the two polymers demonstrated complete substitution with 1‐octanethiol, which was also supported by the observation that both alkyl‐functionalized polymers were found to be more soluble after the reaction, where they were extracted with chloroform. This allows analysis by GPC (Table S1 and Figure S5), with values of *M*_n_ = 29 kDa and *Đ* = 2.2 (PNDI‐T(SR)BTT) and *M*_n_ = 41 kDa and *Đ* = 2.2 (PNDIV‐T(SR)BTT). These are not directly comparable to the values for the starting polymers, which were insoluble and therefore could not be analyzed by GPC. The enhanced solubility is likely related to twisting in the polymer backbone as a result of the bulky thioether groups,^[^ ^42^ ^]^ reducing the propensity for aggregation. Supporting evidence for this theory can be found in the density functional theory (DFT) optimized structures, showing increased twisting of the thiol‐substituted polymers relative to the parent fluorinated polymers (Table S2).

### Quantitative Postpolymerization Modification

**Figure 1:**
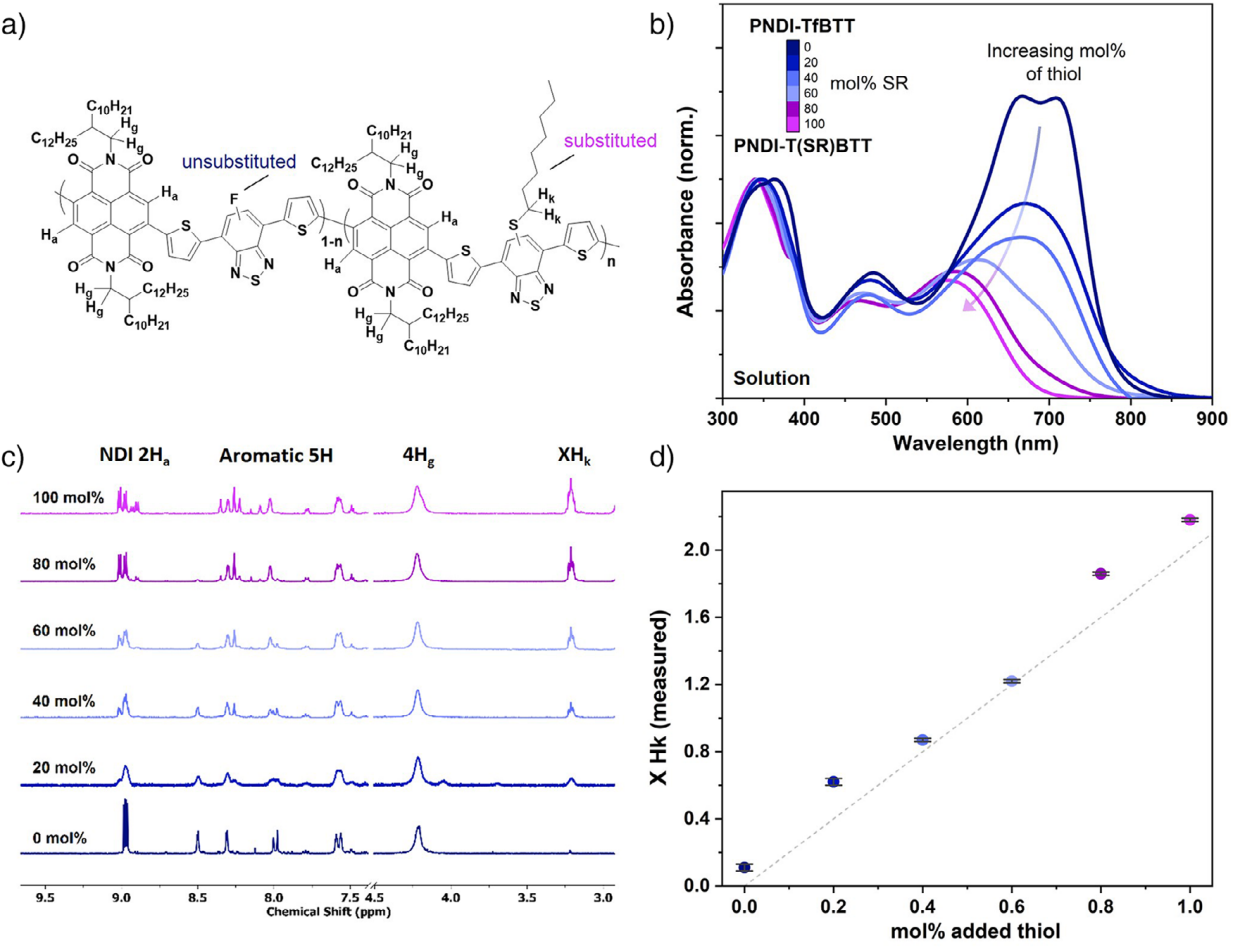
Quantitative reaction of 1‐octanethiol with the PNDI‐TfBTT backbone. a) Structure of partially substituted PNDI‐T(SR)BTT. b) UV–vis absorption spectra in chlorobenzene (normalized to the peak at 350–370 nm) for the unsubstituted PNDI‐TfBTT (0 mol%), partially substituted (20, 40, 60, and 80 mol%) and fully substituted (100 mol%) PNDI‐T(SR)BTT polymers. c) ^1^H NMR spectra of PNDI‐TfBTT as a function of increasing 1‐octanethiol inclusion along the backbone where the aromatic peaks for H_a_ were set to an integral of 2H and directly compared with XH_k_. d) Measured (from ^1^H NMR) XH_k_ relative intensities as a function of thiol concentration used in the reaction. Error bars indicate the peak integration signal divided by the signal‐to‐noise ratio.

After confirming that complete S_N_Ar PPM was possible on the n‐type polymers using excess 1‐octanethiol, we investigated whether the process could be tuned quantitatively by controlling the molar quantity of the side chain added to PNDI‐TfBTT. We performed S_N_Ar PPM reactions with defined (0, 20, 40, 60, 80, and 100 mol%) quantities of thiol reactant (relative to the mass of the polymer repeat unit) and studied the properties of the resulting polymers (Figure 1a).

The UV–vis absorbances of the polymers shown in Figure 1b demonstrate that, up to 40 mol% substitution, the absorption band edge did not change significantly in terms of wavelength but there is a clear reduction in intensity of the long wavelength peak relative to the higher energy absorption peaks. Upon further substitution (60 mol% or greater), there was a significant blue shift in the band edge, which can be associated with the attachment of the thioether substituent and its effect on conjugation length as observed in similar investigations with F8fBT.^[^ ^26^ ^] 1^H NMR investigation showed that, as the quantity of thiol added was increased, the integrated triplet signal at *δ* 3.21 ppm (─CH_2_─ adjacent to the thioether, *H*_k_) increased relative to the signal at *δ* 8.98 ppm (corresponding to hydrogen atoms of the NDI unit, *H*_a_; Figure 1c). Note a very weak peak present in the unreacted polymer (0 mol%) at a similar chemical shift is related to the presence of methanol impurity. A similar trend was observed in the ^19^F spectra, where the benzothiadiazole fluorine environment peak decreased with increasing octanethiol mol% (Figure S11). When comparing the relative integrations of the *H*_g_ and *H*_k_, the degree of substitution along the polymer backbone was found to increase approximately quantitatively (Figure 1d and Table S3). Collectively, these data confirmed the quantitative inclusion of the thioether along the polymer backbone, and we next set out to examine the resulting effects on the polymer properties.

### Polymer Optical, Morphological, and Electronic Properties

**Figure 2:**
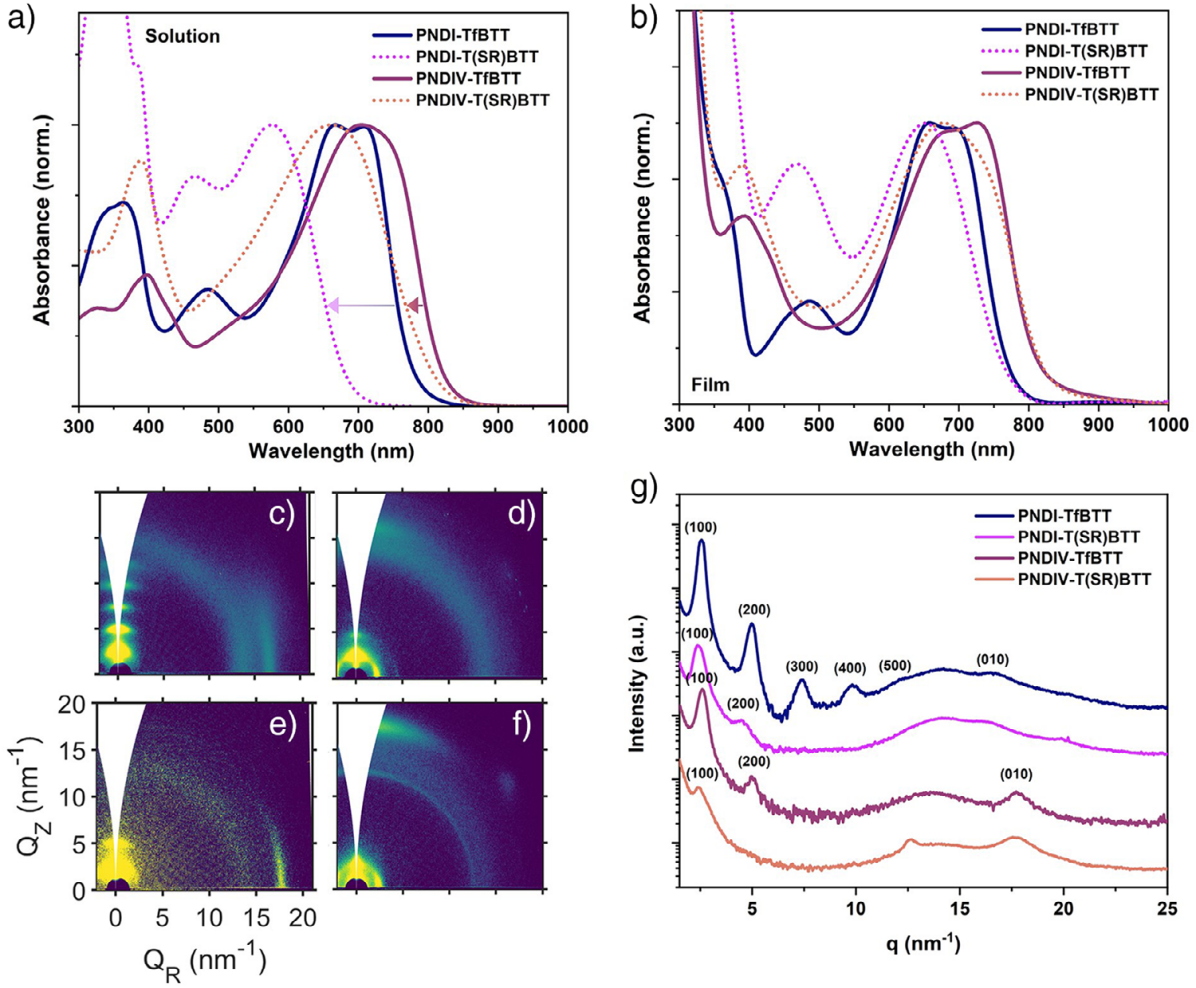
a) UV–vis absorption spectra for PNDI‐TfBTT, PNDI‐T(SR)BTT, PNDIV‐TfBTT, and PNDIV‐T(SR)BTT in chlorobenzene, and b) as a spun‐cast thin film onto glass. c)–f) 2D GIWAXS diffraction images for c) PNDI‐TfBTT, d) PNDIV‐TfBTT, e) PNDI‐T(SR)BTT, and f) PNDIV‐T(SR)BTT thin films annealed at 200 °C. g) GIWAXS diffraction patterns (azimuthal integrations) for PNDI‐TfBTT, PNDIV‐TfBTT, PNDI‐T(SR)BTT, and PNDIV‐T(SR)BTT films annealed at 200 °C.

The UV–vis absorption spectra of polymers PNDI‐TfBTT and PNDI‐T(SR)BTT, both in solution (chlorobenzene) and film, were examined (Figure 2a,b and Table S4). Both polymers exhibited three principal absorption bands in solution, with two high‐energy bands attributed to the π‐π* transition of the NDI and fBT units (300–400 and 400–525 nm, respectively) and a third lower energy band (525–800 nm) linked to intramolecular charge transfer (ICT) from the electron‐rich thiophene to the electron‐poor NDI/fBT moieties. The spectra are similar to reported analogues with difluorinated or nonfluorinated BT.^[^ ^43^, ^44^ ^]^ Thioether substitution resulted in a notable blue‐shift of the ICT absorption peak from 710 to 575 nm in PNDI‐T(SR)BTT likely due to backbone twisting that reduces the effective conjugation length, as well as the replacement of the electron‐withdrawing fluorine with a donating thioether. The π‐π* and ICT transition was also observed in thin films, but the blue shift for PNDI‐T(SR)BTT was less pronounced than in solution, indicating a lesser backbone twist in aggregated states. For the vinylene‐based polymers in solution, similar phenomena were found, but the changes were less pronounced. We posit that this is due to the smaller twisting of the backbone relative to PNDI‐TfBTT as a result of the vinylene group acting as a spacer between the NDI and substituted BTT units. Interestingly, all polymers were shown to be somewhat photoluminescent in solution, with the luminescence originating from the ICT excited states (Figure S12).

In the solid‐state UV–vis absorption spectra (Figure 2b), the unsubstituted polymers (PNDI‐TfBTT and PNDIV‐TfBTT) exhibited pronounced long‐wavelength absorption bands extending to about 800 nm, characteristic of strong intermolecular interactions and efficient ICT. When the fluorine atoms were replaced with thioether substituents (PNDI‐T(SR)BTT and PNDIV‐T(SR)BTT), the main ICT peak blue‐shifted, and the vibronic structure changed while the overall absorption onset (and thus the extracted bandgap) remained similar. We attribute this blue‐shift primarily to increased twisting in the polymer backbone upon thioether substitution, which disrupts extended conjugation and slightly weakens π‐π interactions. Nonetheless, the bulk electronic structure, reflected in the bandgap, and the vibronic structure of these copolymers did not fundamentally change as a function of the substituent because the donor–acceptor framework remained largely preserved.

The solid‐state microstructure is known to have a profound impact on the carrier mobility of NDI polymers.^[^ ^45^, ^46^, ^47^ ^]^ The microstructure of thin‐films was probed using grazing‐incidence wide‐angle X‐ray scattering (GIWAXS), which were spun from chlorobenzene directly onto silicon wafers and analyzed in various states: as‐cast, post annealing for 30 mins at 100 °C, and after a 30‐min annealing at 200 °C (Figure 2c–f, with supporting data in Figures S13, S14, and calculated crystal correlation length (CCL) (100) and (010) values in Table S5). The structural ordering and crystal orientation of the films improved with annealing in all cases, and here we focus our discussion on the films annealed at 200 °C. Distinct diffraction peaks (100), corresponding to interlayer d‐spacings of 24.6 and 24.1 Å (measured from the azimuthal integrations of the 2D GIWAXS patterns, Figure 2g) were observed for PNDI‐TfBTT, and PNDIV‐TfBTT, respectively, signifying lamellar‐type packing of the conjugated polymer strands.^[^ ^48^ ^]^ PNDI‐TfBTT displayed an edge‐on orientation, with up to five orders (500) of reflection apparent, suggestive of good structural coherence of the lamellar‐like order of the crystallites. Introduction of the vinylene group resulted in a slight reduction in lamellar spacing. Additionally, fewer diffraction peaks were observed, while the predominately edge‐on orientation remained intact. For both polymers, π‐π stacking (Figure S14), diffraction peaks corresponding to d‐spacings of 3.8 and 3.5 Å, respectively, were observed. The latter is similar to that previously observed for NDIV polymers and demonstrates a known phenomenon of reduced π‐π stacking distances due to the presence of the vinyl spacer that facilitates packing of the conjugated backbones.^[^ ^40^, ^41^ ^]^ Collectively, these data suggest that the vinylene group increases packing density, both in the lamellar and π‐π packing directions, but reduces the overall crystallinity.

Upon substitution with the thioether group, a lamellar spacing of 26.2 Å was observed for both polymers, representing a small increase relative to the parent polymers. The π‐π distance was unchanged for PNDI‐T(SR)BTT and slightly increased to 3.6 Å for PNDIV‐T(SR)BTT. Notably, both polymers appeared to exhibit a lower tendency to pack in lamellar stacks compared to their parent materials, which is transduced into weaker (100) peaks. Moreover, contrarily to PNDI‐TfBTT and PNDIV‐TfBTT, face‐oriented crystallites were found to dominate in PNDI‐T(SR)BTT and PNDIV‐T(SR)BTT samples (although edge‐on orientations were also present). We attribute the increase in lamellar spacing to increased steric bulk resulting from the thioether groups. The change to a predominate face‐on orientation may result from decreased solution aggregation for the thioether substituted polymers, due to the increase in the number of solubilizing groups and the backbone twisting induced by the thioether. Previous studies have found that reducing preaggregation in solution can favor face‐on packing upon spin casting.^[^ ^49^, ^50^, ^51^ ^]^ For all polymers, maximal morphological order, and thus anticipated increased mobility, was achieved when annealed at 200 °C.

The measured HOMO and LUMO energy levels of the polymer thin films are presented in Table 1 in comparison to those calculated from the DFT optimized structures (with visualizations of the orbitals in Figures S15–S18). The HOMO energy levels of PNDI‐TfBTT, PNDIV‐TfBTT, PNDI‐T(SR)BTT, and PNDIV‐T(SR)BTT were measured (by photoelectron spectroscopy in air (PESA), Figure S19) to be −5.67, −5.53, −5.47, and −5.35 eV, respectively, while the LUMO energy levels were estimated by the addition to the optical band gap to be −4.07, −3.92, −3.94, and −3.85 eV, respectively. The estimated frontier orbital energy levels from the DFT calculations deviate from the experimentally determined values by a few tenths of eV, however, the relative differences broadly agree with these observed trends. For all polymers, the HOMOs were found to center on the more electron rich dithienyl‐benzothiadiazole (T‐BT‐T) core, while the LUMOs had a larger wavefunction density on, and spanned across, the flanking NDI groups. For the parent polymers, the slight increase in HOMO and LUMO levels after inclusion of the vinylene group can be explained by a combination of the addition of the electron rich double bond and reduced level of twisting in the backbone.^[^ ^52^, ^53^ ^]^ A similar trend after thioether substitution can be explained by the displacement of the strongly electron‐withdrawing fluoride group by the mildly electron‐donating ‐SR group, which would be expected to increase both HOMO and LUMO energy levels.^[^ ^42^, ^54^ ^]^

**Table 1:** Electronic properties of PNDI‐TfBTT, PNDIV‐TfBTT, PNDI‐T(SR)BTT, and PNDIV‐T(SR)BTT.

Polymer	*E*_g,opt_ ^a)^ (eV)	HOMO^b)^ (eV)	LUMO^c)^ (eV)	HOMO (eV) DFT	LUMO (eV) DFT
**PNDI‐TfBTT**	1.60	−5.67	−4.07	−5.55	−3.51
**PNDIV‐TfBTT**	1.53	−5.47	−3.94	−5.40	−3.51
**PNDI‐T(SR)BTT**	1.61	−5.53	−3.92	−5.54	−3.45
**PNDIV‐T(SR)BTT**	1.50	−5.35	−3.85	−5.41	−3.43

^a)^Estimated from the onset of absorption from the measured UV–vis spectra where E = hc/λ;

^b)^Measured by PESA;

^c)^LUMO = HOMO + E_g,opt_

### Thermal Properties of the Polymers

Thermogravimetric analysis (TGA) and differential scanning calorimetry (DSC) techniques were used to investigate the degradation, melt, and recrystallisation temperatures for PNDI‐TfBTT, PNDI‐T(SR)BTT, PNDIV‐TfBTT, and PNDIV‐T(SR)BTT, shown in Figure S20.

In TGA analysis, heating polymers from 25 to 750 °C under nitrogen revealed good thermal stability in PNDI‐TfBTT, PNDIV‐TfBTT, PNDI‐T(SR)BTT, and PNDIV‐T(SR)BTT, with degradation temperatures of 441, 413, 346, and 393 °C, respectively, showing a slight decrease in stability with substitution. In DSC analysis, PNDI‐TfBTT exhibited distinct melt (347 °C) and recrystallisation peaks (322 °C). Upon substitution with the thioether group, no thermal transitions were observed, although heating was limited to 300 °C due to the relatively low decomposition temperature. PNDIV‐TfBTT did not display a clear melting and recrystallization peak, although weak endotherm (141 °C) and exotherm (112 °C) transitions were visible. These disappeared upon thioether substitution, with PNDIV‐T(SR)BTT exhibiting clear melt (324 °C) and recrystallization (315 °C) transitions. From analysis of the TGA and DSC data, an annealing temperature range between 120 and 250 °C was determined suitable for exploring the electrical properties of the polymers in OTFT devices.

### OTFT Charge Transport Properties of the Thioether Polymers

To investigate the electron charge‐carrier mobility (*µ*_e_) for each polymer, OTFTs were fabricated with a top‐gate, bottom‐contact (TG/BC) configuration, using gold source and drain electrodes, poly(methyl methacrylate) (PMMA) as the gate dielectric, and an aluminum gate electrode (Figure S21). The polymers were deposited from 1,2‐dichlorobenzene solutions (5 mg mL⁻¹) via spin coating. The thin films were then annealed at temperatures ranging from 120 to 250 °C for 30 min, followed by spin‐coating of PMMA and annealing at 95 °C for 2 h. Finally, the Al gate electrode was deposited by thermal evaporation under high vacuum.

**Figure 3:**
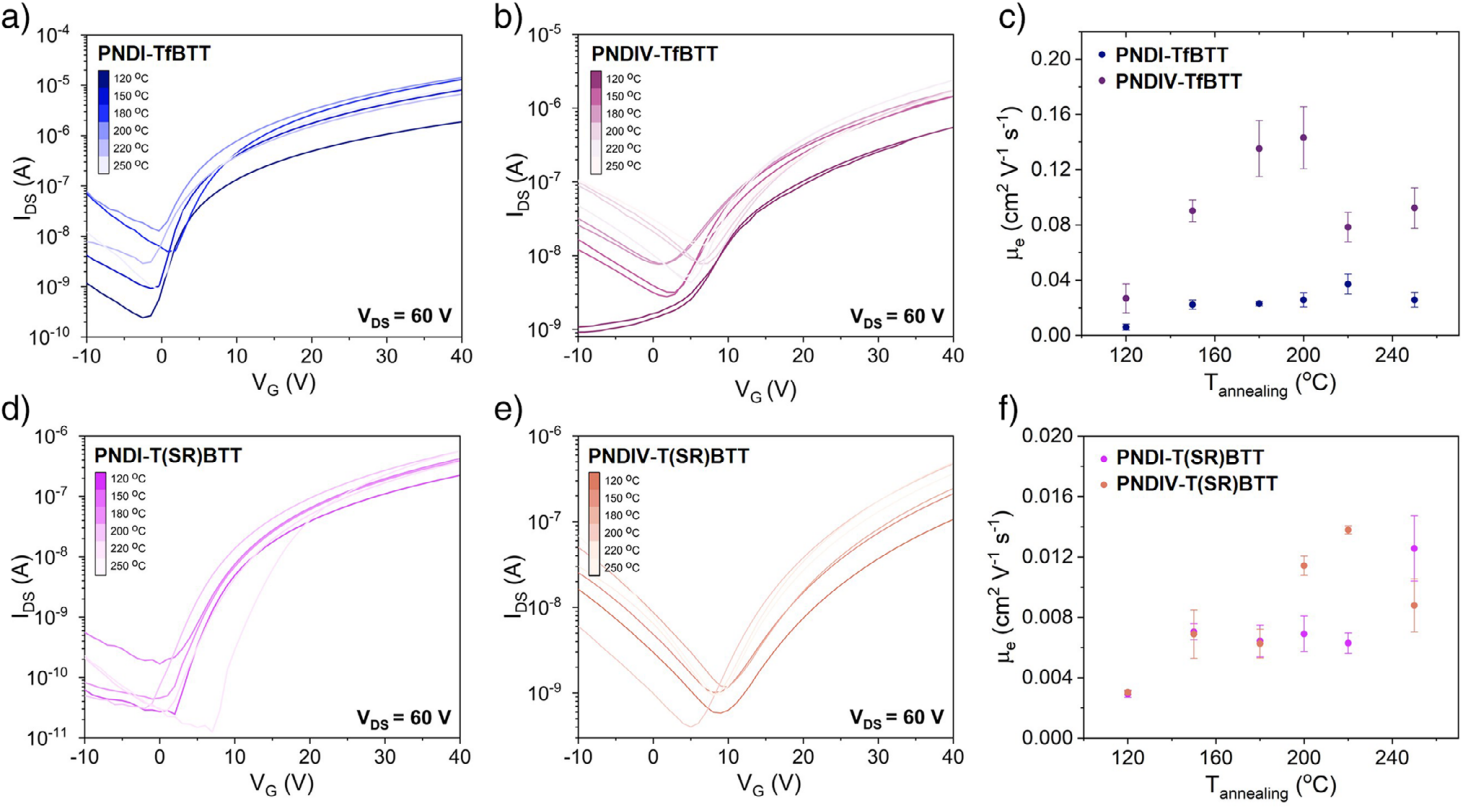
a), b) Transfer characteristics of a) PNDI‐TfBTT and b) PNDIV‐TfBTT OTFT devices. c) Electron mobility of PNDI‐TfBTT and PNDIV‐TBTT OTFT devices as a function of annealing temperature of the polymer thin film. d), e) Transfer characteristics of d) PNDI‐T(SR)BTT and e) PNDIV‐T(SR)BTT OTFT devices. f) Electron mobility of PNDI‐T(SR)BTT and PNDIV‐T(SR)BTT OTFT devices as a function of annealing temperature of the polymer thin film. Transistor channel length 30 µm and channel width 1000 µm.

Transfer characteristics under a positive gate bias for PNDI‐TfBTT and PNDIV‐TfBTT, along with the electron mobility as a function of annealing temperature, are presented in Figure 3a–c, with similar data for PNDI‐T(SR)BTT and PNDIV‐T(SR)BTT also shown in Figure 3d–f with a summary of all the data in Table S6. Here, we discuss the devices that were annealed at 200 °C. PNDI‐TfBTT exhibited an electron mobility of 0.16 cm^2^ V^−1^ s^−1^, the highest of all the polymers. A reduction by over two‐thirds to 0.047 cm^2^ V^−1^ s^−1^ was noted for the electron mobility of PNDIV‐TfBTT compared to PNDI‐TfBTT, aligning with thin film morphology studies which indicated lower crystallinity for PNDIV‐TfBTT. Previous reports have affirmed a similar reduction in thin film crystallinity and charge carrier mobility with the incorporation of a vinylene linker within the polymer backbone.^[^ ^55^, ^56^ ^]^ Post thioether substitution, the induced twisting in the backbone was expected to reduce the electron mobility,^[^ ^57^ ^]^ and a reduction in electron mobility was indeed observed for both PNDI‐T(SR)BTT (0.015 cm^2^ V^−1^ s^−1^) and PNDIV‐T(SR)BTT (0.014 cm^2^ V^−1^ s^−1^) compared to the unsubstituted parent polymers PNDI‐TfBTT (0.16 cm^2^ V^−1^ s^−1^), and PNDIV‐TfBTT (0.047 cm^2^ V^−1^ s^−1^), respectively. The reduction in electron mobility correlates with the reduced degree of crystallinity, and the mix of face‐on and edge‐on crystallites for these polymers as demonstrated by the GIWAXS data. For all polymers, the highest electron mobility was observed at an annealing temperature of 200 °C or higher (Table S6). This is also in agreement with observations from the GIWAXS data where crystalline domains were maximized after annealing at 200 °C or above, with a higher degree of microstructural order within deposited and annealed thin films known to increase CP charge carrier mobility.^[^ ^45^, ^58^, ^59^ ^]^

### Inclusion of Azide Functionality on PNDI‐TfBTT

Thus far in this study, PNDI‐TfBTT has shown significant promise as an n‐type polymer capable of quantitative backbone editing via S_N_Ar on the backbone. Although thioether substitution led to increased solubility of the polymer, aiding processing and the fabrication of OTFT devices, we hypothesized that we could introduce multifunctionality via the substituent. Specifically, with the inclusion of an azide (─N_3_) functional group at the end of a short alkyl chain, we envisaged the preparation of an n‐type polymer that could be thermally and photochemically crosslinked^[^ ^60^, ^61^ ^]^ and, therefore, immobilized on a surface while retaining good electron mobility. We note that azide functionality is also an excellent handle for further potential functionalization via Huisgen type click reactions with functional alkynes.^[^ ^62^, ^63^ ^]^

**Scheme 2:**
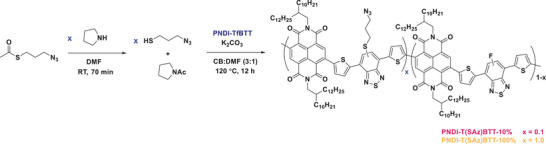
Postpolymerization modification of PNDI‐TfBTT with 3‐azidopropane‐1‐thiol in 10% and 100% relative molar equivalents.

**Figure 4:**
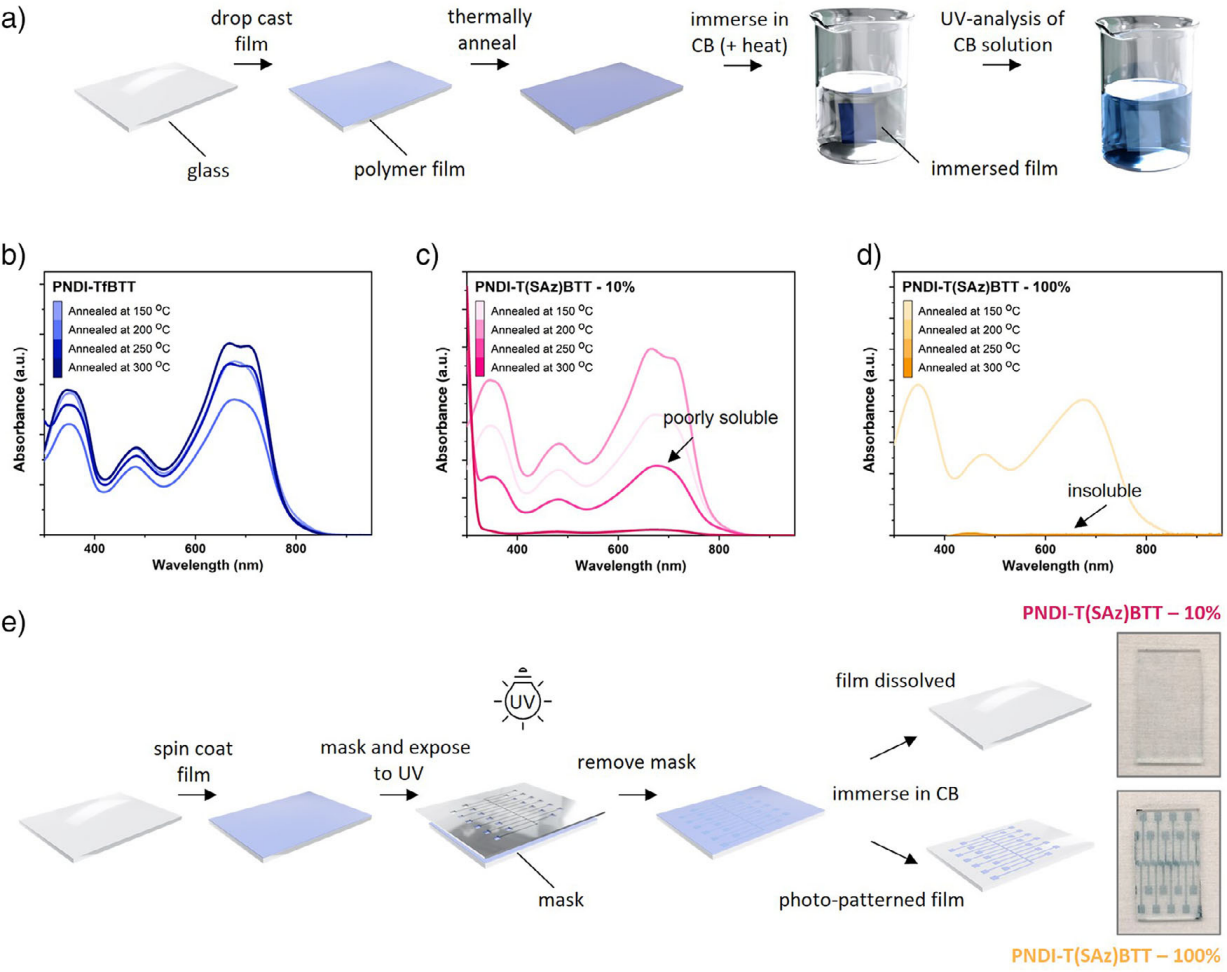
Thermal crosslink studies of PNDI‐TfBTT, PNDI‐T(SAz)BTT – 10%, and PNDI‐T(SAz)BTT – 100%. a) Drop‐cast films were annealed at 150, 200, 250, and 300 °C for 30 mins under N_2_ before immersing in chlorobenzene where the resulting solution was analyzed by UV–vis. b)–d) UV–vis absorption spectra for solutions from b) of PNDI‐TfBTT, c) PNDI‐T(SAz)BTT – 10%, and d) PNDI‐T(SAz)BTT – 100%. e) Schematic of the photocrosslinking studies where spin coated films were exposed to UV light (254 nm) for 60 mins under N_2_ before immersion in chlorobenzene for 24 h. Photographs of the resulting uncoated glass (with most of the polymer having dissolved) and photopatterned film (with only the nonUV‐cured polymer having dissolved) for PNDI‐T(SAz)BTT – 10%, and PNDI‐T(SAz)BTT – 100%, respectively, are shown to the right of the schematic.

Scheme 2 shows the synthesis of the two azide‐functionalized PNDI‐TfBTT polymers using PPM via S_N_Ar: PNDI‐T(SAz)BTT – 10% (low azide content) and PNDI‐T(SAz)BTT – 100% (high azide content). To synthesize 3‐azidopropane‐1‐thiol in situ, commercially available S‐(3‐azidopropyl)thioacetate was deacylated by reaction with pyrrolidine in DMF at room temperature for approximately 70 mins. The resulting mixture was added to a solution of PNDI‐TfBTT (insoluble fraction) and K_2_CO_3_ in chlorobenzene/DMF (3:1) and reacted at 120 °C overnight in an oil bath (Figure 4a). For PNDI‐T(SAz)BTT – 10% (low azide content) and PNDI‐T(SAz)BTT – 100% (high azide content), 10 mol% and 100 mol% of 3‐azidopropane‐1‐thiol were reacted relative to the polymer repeat unit, respectively. The mixtures were precipitated into methanol and purified by Soxhlet extraction to remove the acetylpyrrolidine (formed in the synthesis of 3‐azidopropane‐1‐thiol), excess base and any unreacted 3‐(azidopropyl)thioacetate. In the final Soxhlet washing step, the polymers PNDI‐T(SAz)BTT – 10% and PNDI‐T(SAz)BTT – 100% were extracted with chloroform. PNDI‐T(SAz)BTT – 100% was mostly dissolved with chloroform to afford a dark blue solid after evaporation of the solvent (69% yield). The partially substituted polymer PNDI‐T(SAz)BTT – 10% could not be dissolved in chloroform, and it was collected from the Soxhlet thimble and dried to give a dark blue solid (85% yield). The poor solubility resulted in a high tendency to aggregate, particularly for PNDI‐T(SAz)BTT – 10%, which was reflected in the apparent GPC data (*M*_n_ = 100 kDa, *Đ* = 2.5, Figure S22 and Table S7). PNDI‐T(SAz)BTT – 100% also exhibited some signs of aggregation, but GPC values (*M*_n_ = 23 kDa, *Đ* = 2.8) were close to those of PNDI‐T(SR)BTT.

The successful reaction of 3‐azidopropane‐1‐thiol and introduction of the azide groups along the polymer backbone was confirmed initially by Fourier transform infrared spectroscopy (FTIR) (Figure S23), where the IR spectra of both PNDI‐T(SAz)BTT – 10% and PNDI‐T(SAz)BTT – 100% displayed an absorption peak at 2100 cm^−1^ corresponding to the azide stretch. The distinct absorption peak also demonstrated that the attached azide group remained intact after the S_N_Ar reaction. Similarly, UV–vis absorption spectra demonstrated a distinct decrease in the ICT absorption with increasing azide substitution. Furthermore, ^1^H and ^19^F NMR (Figures S24–S27) data confirmed the displacement of the fluoride on the fBT with the azide‐containing substituent due to the appearance of two new triplet peaks in the ^1^H NMR spectra at *δ* 3.53 and *δ* 3.25 ppm, corresponding to the ─CH_2_─N_3_ and ─S─CH_2_─ protons from the substituted pendant group, and a reduction in the intensity of the single fluorine environment. The ^1^H NMR data, although with a relatively large signal to noise ratio, especially for PNDI‐T(SAz)BTT 10%, allowed us to quantify the degree of substitution for PNDI‐T(SAz)BTT – 10% and PNDI‐T(SAz)BTT – 100% to be 15% and 100%, respectively (Table S8). Although ^1^H NMR suggests complete substitution, a small signal is apparent in ^19^F NMR, which suggests some backbone fluorine is retained. The lower substitution in the −100% polymer and the higher for the nominal 10% polymer likely relate to the difficulty in accurately added small amounts of nucleophile to the reaction, in combination with the integration errors related to the signal to noise values.

### Thermal Crosslinking of the Azide Polymers

Azide‐containing polymer thin films can be thermally crosslinked by simply annealing the thin film in an inert atmosphere.^[^ ^64^, ^65^ ^]^ Using TGA and DSC measurements, the thermal crosslink temperature range for two polymers was determined, as displayed in Figure S28. From the TGA traces, both polymers were shown to thermally degrade at 400 °C or above. Between 230 and 300 °C, a minor mass reduction of the sample was measured, corresponding to nitrogen (N_2_) loss from the azide group and signaling the onset of crosslinking. A more prominent mass loss was measured for PNDI‐T(SAz)BTT – 100% than PNDI‐T(SAz)BTT – 10%, with theoretical N_2_ mass losses calculated to be 2.0% and 0.2%, respectively. From the DSC curves, an exothermic peak was observed within 230 to 285 °C and 210 to 295 °C ranges for PNDI‐T(SAz)BTT – 10% and PNDI‐T(SAz)BTT – 100%, respectively. Liberation of N_2_ within the same temperature range was indicated by both TGA and DSC data, confirming thermal crosslinking of the thin films between 250 and 300 °C.

The effect of thermal crosslinking on thin film morphology was investigated by drop‐casting chlorobenzene (10 mg mL^−1^) solutions of PNDI‐TfBTT, PNDI‐T(SAz)BTT – 10%, and PNDI‐T(SAz)BTT – 100% onto glass substrates, which were then annealed at four different temperatures: 150, 200, 250, and 300 °C, and immersed in hot chlorobenzene (Figure 4a). It was anticipated that thermally crosslinked polymers would form a high molecular weight network, yielding thin films insoluble in hot chlorobenzene. The resulting chlorobenzene solutions were analyzed by UV–vis (Figure 4b–d) to detect any redissolved noncrosslinked material. In the case of PNDI‐TfBTT, all four samples, post annealing at 150, 200, 250, and 300 °C, redissolved in the hot chlorobenzene. For PNDI‐T(SAz)BTT – 10%, films annealed at 150 and 200 °C redissolved in hot chlorobenzene, demonstrating the presence of noncrosslinked polymer. When annealed at 250 °C, the sample was only partially soluble, and the resulting thin film delaminated from the glass slide. Complete insolvency and no delamination were observed at 300 °C, indicating some degree of crosslinking. PNDI‐T(SAz)BTT – 100% thin films, annealed at 150 °C, partially dissolved, while those annealed at 200, 250, and 300 °C showed no signs of dissolution or delamination from UV data, suggesting a high degree of crosslinking and thermal immobilization.

### Photochemical Crosslinking of the Azide Polymers

In order to further explore the crosslinking functionality of the azide‐containing polymers, the ability of the polymer thin films to crosslink upon exposure to UV light was studied. As in the case of thermal crosslinking, exposing the azide‐containing polymer to UV light liberates N_2_, leaving behind a reactive nitrene radical that initiates multiple crosslinking mechanisms.^[^ ^61^, ^66^ ^]^ For the photocrosslink experiments, polymers dissolved in chlorobenzene (10 mg mL^−1^) were spin‐coated onto glass substrates and irradiated in an inert atmosphere with UV light (254 nm) for 1 h through a patterned mask. The samples were then immersed in chlorobenzene, and the solubility of the resulting thin films analyzed (Figure 4e).

For PNDI‐T(SAz)BTT ‐ 10%, the resulting patterned material did not remain completely intact on the glass substrate after immersion in chlorobenzene. Closer inspection of the sample revealed slight retention of the polymer on the glass slide, indicating partial crosslinking. For PNDI‐T(SAz)BTT – 100%, the resulting patterned material remained intact on the glass substrate after immersion in chlorobenzene, showing a higher degree of azide content is required for photochemical crosslinking.

### Thin Film Morphology Post Crosslinking

To determine the changes in the microstructure of PNDI‐T(SAz)BTT – 10% and PNDI‐T(SAz)BTT – 100% after crosslinking, we investigated the thin film morphologies of the polymers spun on silicon wafer substrates from chlorobenzene (10 mg mL^−1^) and after exposure to i) different annealing temperatures (200 and 300 °C in an inert atmosphere for 30 mins), ii) to UV light (254 nm) for 1 h, and iii) UV light followed by thermal annealing.

**Figure 5:**
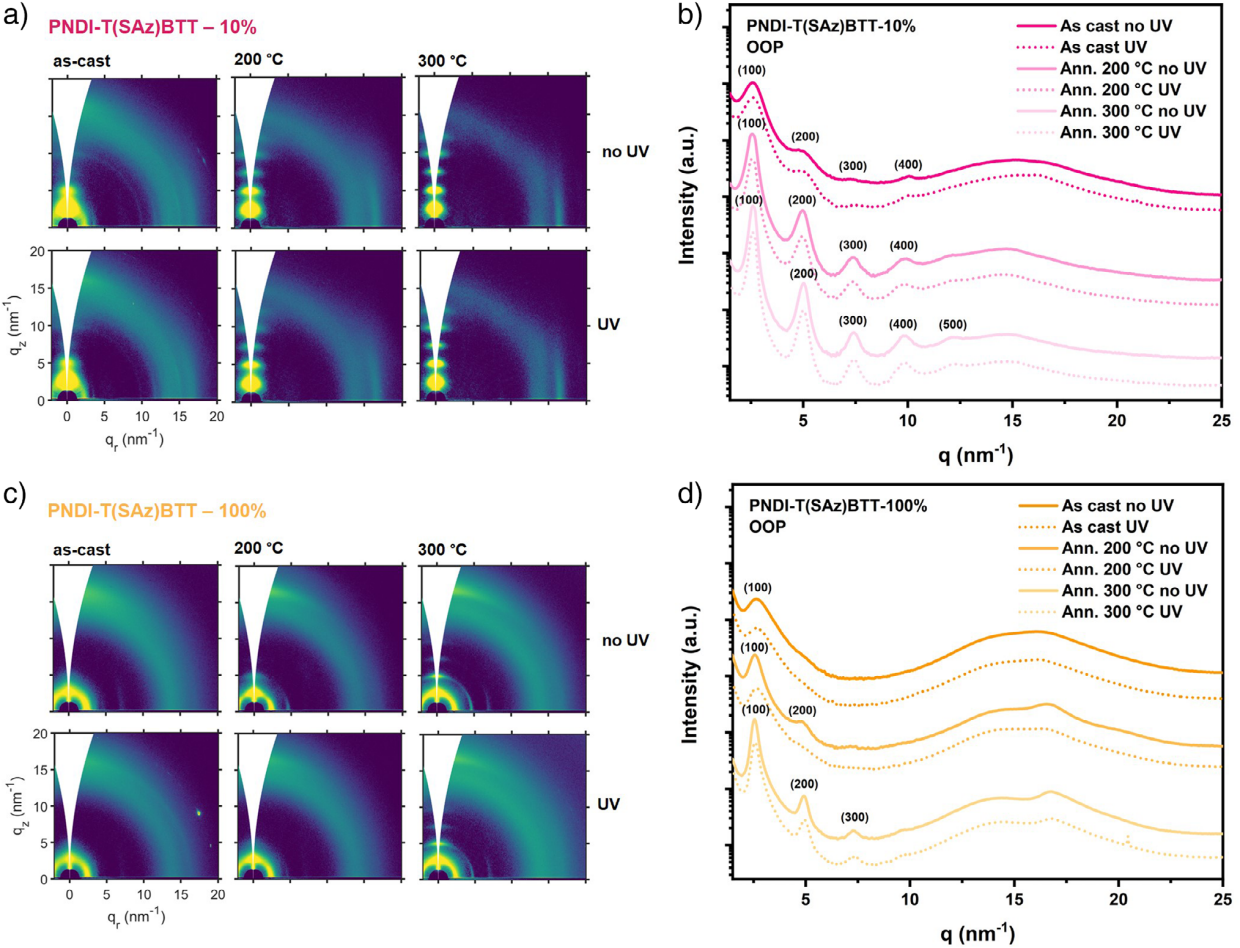
a), b) GIWAXS images a) and GIWAXS diffraction patterns (out‐of‐plane) b) of PNDI‐T(SAz)BTT – 10% films as‐cast (no thermal anneal), and after annealing at 200 and 300 °C, with and without pre‐exposure to UV light (254 nm for 60 min). c), d) GIWAXS images c) and GIWAXS diffraction patterns (out‐of‐plane) d) of PNDI‐T(SAz)BTT – 100% films as‐cast (no thermal anneal), and after annealing at 200 and 300 °C, with and without pre‐exposure to UV light (254 nm for 60 min).

To assess the impact of these treatments on thin film morphology, GIWAXS investigations were carried out (Figures S29, S30, Table S9). The thermally‐annealed thin films of PNDI‐T(SAz)BTT – 10% (as‐cast, annealed at 200 °C, and at 300 °C) exhibited lamellar‐type packing with diffraction peaks (100) in the out‐of‐plane orientation at 2.56, 2.57, and 2.59 nm^−1^ corresponding to interlayer d‐spacings of 24.5, 24.4, and 24.3 Å, respectively (Figure 5a,b), with an edge‐on orientation; all similar to the parent PNDI‐TfBTT which is perhaps unsurprising given the relatively low degree of thioazide substitution. Although the as‐cast sample showed lamellar peaks up to the second order (200), the thermally annealed thin films became more ordered with a further advancement up to the fourth (400) and fifth order (500) in the lamellar peaks when annealed at 200 and 300 °C, respectively. Moreover, the π‐π stacking diffraction peak (predominantly oriented in the in‐plane) became more prominent after annealing, indicating a higher degree of order, where the π‐π stacking peaks found at 16.5 to 16.7 nm^−1^ corresponds to a d‐spacing of 3.8 Å, again similar to PNDI‐TfBTT. After exposure of the samples to UV, the GIWAXS patterns did not significantly change, with no evidence of alterations to the structure or orientation of the polymer film. This is likely due to insufficient azide content to drive effective photocrosslinking.

The as‐cast PNDI‐T(SAz)BTT – 100% film showed a reduced degree of order compared to PNDI‐T(SAz)BTT – 10%, similar to the reduction in order observed for PNDI‐TfBTT to PNDI‐T(SR)BTT. All thin films of PNDI‐T(SAz)BTT – 100% (as‐cast, annealed at 200, and at 300 °C) showed lamellar‐type packing with diffraction peaks (100) in the out‐of‐plane orientation at 2.63, 2.56, and 2.55 nm^−1^ corresponding to interlayer d‐spacings of 23.9, 24.5, and 24.6 Å, respectively (Figure 5c,d), with a mixed face‐on and edge‐on orientation akin to the thioether‐substituted polymers. For the as‐cast thin film, first order lamellar peaks (100) were observed; however, for the thin films annealed at 200 and 300 °C, lamellar peaks up to the second (200) and third order (300), respectively, were present. From the GIWAXS data, it was possible to conclude that thermally crosslinking PNDI‐T(SAz)BTT – 100% also leads to a higher degree of crystallinity as compared to the as‐cast films. When the thin film was exposed to UV light before thermal annealing, initially a new isotropic reflection (prominently oriented in the in‐plane) appeared at 16.2 nm^−1^ (d‐spacing 3.9 Å), as shown in Figure S29, indicating a reduction in overall crystallinity as compared to the as‐cast film. However, this reflection was found to disappear following annealing at 200 or 300 °C. We propose that partial crosslinking during UV exposure initially “freezes” this more disordered morphology, and subsequent annealing provides enough chain mobility for local reorganization prior to full network formation, thereby recovering the crystallinity. Moreover, the loss of molecular nitrogen and the formation of nitrene intermediates upon azide decomposition may simultaneously reduce backbone disorder and allow closer interchain contacts, further driving the observed re‐establishment of the packing observed in the parent polymer.^[^ ^67^, ^68^ ^]^ AFM characterization of the films (Figure S31) was largely in agreement with the trends observed from the GIWAXS data.

### OTFT Charge Transport Properties of the Crosslinked Polymers

To understand the effects of crosslinking on electron charge carrier mobility of PNDI‐T(SAz)BTT – 10% and PNDI‐T(SAz)BTT – 100%, OTFT devices were fabricated with a TG/BC configuration in which the polymer thin film active layers were spin‐coated and i) thermally annealed at 200 or 300 °C for 30 mins, ii) exposed to UV‐light (254 nm) for 1 h, or iii) both exposed to UV‐light (254 nm) for 1 h and then thermally annealed at 200 or 300 °C for 30 mins. The transfer characteristics for PNDI‐T(SAz)BTT – 10% and PNDI‐T(SAz)BTT – 100% under a positive gate bias are shown in Figures S32–S35, with the electron mobility data presented in Table 2.

**Table 2:** Summary of OTFT data (electron transport) for PNDI‐T(SAz)BTT – 10% and PNDI‐T(SAz)BTT – 100% with a (TG/BC) configuration.

		No treatment (as‐cast)		Thermal (annealed at 200 °C)		Thermal (annealed at 300 °C)	
Polymer	UV treatment	*µ*_avg_ (cm^2^ V^−1^ s^−1^)	*µ*_max_ (cm^2^ V^−1^ s^−1^)	*µ*_avg_ (cm^2^ V^−1^ s^−1^)	*µ*_max_ (cm^2^ V^−1^ s^−1^)	*µ*_avg_ (cm^2^ V^−1^ s^−1^)	*µ*_max_ (cm^2^ V^−1^ s^−1^)
**PNDI‐T(SAz)BTT – 10%**	No	1.4 × 10^−2^	1.6 × 10^−2^	4.0 × 10^−2^	5.0 × 10^−2^	7.7 × 10^−2^	9.8 × 10^−2^
	Yes	1.5 × 10^−2^	2.2 × 10^−2^	–	–	–	–
**PNDI‐T(SAz)BTT – 100%**	No	8.1 × 10^−3^	1.0 × 10^−2^	1.3 × 10^−2^	1.6 × 10^−2^	7.4 × 10^−3^	9.6 × 10^−3^
	Yes	2.1 × 10^−4^	3.0 × 10^−4^	1.5 × 10^−3^	1.8 × 10^−3^	1.3 × 10^−3^	1.7 × 10^−3^

For the as‐cast films, the average electron mobilities for PNDI‐T(SAz)BTT – 10% and PNDI‐T(SAz)BTT – 100% were 1.4 × 10^−2^ and 8.1 × 10^−3^ cm^2^ V^−1^ s^−1^ respectively, with the lower mobility of the latter likely attributable to the larger degree of twisting in the backbone from increased substitution along the polymer backbone. For PNDI‐T(SAz)BTT – 10%, the average electron mobility increased to a high of 7.7 × 10^−2^ cm^2 ^V^−1^ s^−1^ (annealed at 300 °C) as a result of improved ordering and crosslinking, while for PNDI‐T(SAz)BTT – 100% lower average electron mobilities were observed at all annealing conditions, with a high of 7.3 × 10^−3^ cm^2^ V^−1^ s^−1^ (annealed at 200 °C), likely due to greater thermal crosslinking.

In photocrosslinking experiments, when tested in an OTFT device the average electron mobility of PNDI‐T(SAz)BTT – 10% was virtually unchanged from the as‐cast thin film (1.4 × 10^−2^ cm^2 ^V^−1^ s^−1^) to the UV exposed as‐cast thin film (1.5 × 10^−2^ cm^2 ^V^−1^ s^−1^). This can be rationalized by the limited crosslinking of this polymer with UV exposure. The average electron mobility values of PNDI‐T(SAz)BTT – 100% OTFT devices were, however, shown to decrease by an order of magnitude from the as‐cast thin film (8.1 × 10^−3^ cm^2 ^V^−1^ s^−1^) to the UV‐exposed thin film (2.1 × 10^−4^ cm^2 ^V^−1^ s^−1^). The reduction in electron mobility was likely due to a reduction in crystallinity of the polymer upon exposure to UV light, as observed in the GIWAXS data. Gratifyingly, annealing the UV‐exposed samples improved the average mobility to 1.5 × 10^−3^ cm^2 ^V^−1^ s^−1^ (annealed at 200 °C) and 1.3 × 10^−3^ cm^2 ^V^−1^ s^−1^ (annealed at 300 °C), again in line with GIWAXS and AFM data showing increasing crystallinity of the polymer thin films after UV exposure and thermal annealing. These results demonstrate that postpolymerization photocrosslinking is an effective strategy toward achieving immobilized conjugated polymer films that retain good crystallographic order and electron mobility.

## Conclusions

In summary, we prepared two novel n‐type conjugated polymers, PNDI‐TfBTT and PNDIV‐TfBTT, both bearing electron‐withdrawing NDI and fBT units, with PNDIV‐TfBTT further incorporating a vinylene spacer. Compared to PNDI‐TfBTT, PNDIV‐TfBTT exhibited slightly higher‐lying HOMO/LUMO levels, narrower lamellar spacing, and smaller crystalline domain sizes. These differences in electronic structure and packing resulted in a lower electron mobility for PNDIV‐TfBTT relative to PNDI‐TfBTT, which reached 0.14 cm^2^ V⁻¹ s⁻¹ in OTFT devices. Backbone editing by postpolymerization substitution of fluorine atoms with thioalkyl groups enhanced solubility through increased backbone twisting and slightly raised both HOMO and LUMO energy levels. GIWAXS analysis revealed that this substitution also led to widened lamellar spacing and a shifted film orientation partially from edge‐on to face‐on, reducing long‐range order and thereby diminishing electron transport. Building on this work, further exploration focused on incorporating 3‐azidopropanethiol, formed in situ, in 10 mol% and 100 mol% stoichiometries onto PNDI‐TfBTT (yielding PNDI‐T(SAz)BTT – 10%, and PNDI‐T(SAz)BTT – 100%, respectively) to enable both thermal and photochemical crosslinking and thereby create immobilized, conductive active layers. Thermal crosslinking at 300 °C PNDI‐T(SAz)BTT – 10 mol% led to insoluble films displaying electron mobilities up to 0.098 cm^2^ V⁻¹ s⁻¹ in OTFT devices. Annealing PNDI‐T(SAz)BTT – 100% above 200 °C, also led to insoluble films, as did UV photopatterning treatment for 1 h. Although direct photopatterning initially decreased electron transport, subsequent thermal annealing largely restored the mobility. These polymers are promising materials for integration into multicomponent organic electronic devices, with fabrication enabled by their thermal and photochemical crosslinking mechanisms.

## Conflict of Interests

The authors declare no conflict of interest.

## Supporting information



Supporting Information

## Data Availability

The data that support the findings of this study are available from the corresponding author upon reasonable request.
