# Supplementary material for: Preparation of Thermally and Photochemically Immobilized N‐type Conjugated Polymer Films via Quantitative Backbone Editing
Source: Angew Chem Int Ed Engl. 2025 Apr 10;64(23):e202505608. doi: 10.1002/anie.202505608 (PMC12124454; doi:10.1002/anie.202505608)
Supplement: Supplementary file 1 — Supporting Information [file ANIE-64-e202505608-s001.docx]

*Supporting information for*

Preparation of thermally and photochemically immobilized N-type conjugated polymer films via quantitative backbone editing

*Charlotte Rapley^1^, Adam V. Marsh^2^, Edgar Gutierrez-Fernandez^3^, Mohamad Insan Nugraha^2,4^, Flurin Eisner^5^, Martina Rimelle^1^, Jaime Martín^6^, Thomas D. Anthopoulos^7^, Martin Heeney*^1,2^*

^1^ Department of Chemistry and Centre for Processable Electronics, Imperial College London, White City Campus, London, W12 0BZ, UK.

^2^ Department of Physical Science and Engineering, King Abdullah University of Science & Technology (KAUST), Thuwal 23955-6900, Kingdom of Saudi Arabia.

^3^ POLYMAT, University of the Basque Country UPV/EHU Avenida de Tolosa 72, 20018 Donostia-San Sebastián, Spain.

^4^ Research Center for Nanotechnology Systems, National Research and Innovation Agency (BRIN), South Tangerang, Banten 15314, Indonesia

^5^ School of Engineering and Materials Science, Queen Mary University of London, London E1 4NS, UK.

^6^ Universidade da Coruña, Campus Industrial de Ferrol, CITENI, Esteiro, 15471 Ferrol, Spain.

^7^ Henry Royce Institute and Photon Science Institute, Department of Electrical and Electronic Engineering, The University of Manchester, Manchester M13 9PL, UK.

## Supplementary Methods

Synthetic reactions were carried out in an inert nitrogen atmosphere using dry solvents and standard Schlenk line techniques, unless otherwise stated. All analyses and characterisations were carried out in air at room temperature unless otherwise stated.

Microwave-assisted reactions were carried out in a Biotage Initiator+ reactor using Teflon-capped glass vials. All nuclear magnetic resonance (NMR) spectra were recorded on either an AV-400 (400 MHz) or AV-500 (500 MHz) Bruker Spectrometer using d-chloroform (CDCl_3_) or d-trichloroethene (TCE) as solvents. Chemical shift (δ) values are reported in parts per million (ppm) relative to reference solvent signals (CHCl_3_: ^1^H 7.26 ppm, ^13^C 77.16 ppm; and TCE; ^1^H 6.0 ppm, ^13^C 73.78 ppm). Preparative gel permeation chromatography (GPC) of monomers and polymers was carried out on a Shimadzu GPC system running in hexane at 40 °C with an Agilent PLgel 10 µm mixed-d column, DGU-20A3 Degasser, LC-20A Pump, CTO-20A Column Oven, and SPD-20A UV Detector. High-resolution mass spectrometry data were collected using a Thermo Scientific Q-Exactive/Dionex Ultimate 3000 operated in either ES or APCI ionization modes. Photoelectron spectroscopy in air (PESA) measurements were recorded on a Riken Keiki PESA spectrometer (Model AC-2) using a 10 nW power setting and a power number of 0.3. Fourier-transform infrared (FTIR) spectra were recorded on a Cary 630 FTIR spectrometer. Ultraviolet-visible (UV-Vis) absorption measurements were carried out on either an Agilent Cary 60 UV-Vis spectrometer or a Shimadzu UV-1800 UV-Vis spectrometer. Photoluminescence emission (PL) measurements were collected using an Agilent Cary Eclipse fluorescence spectrometer. Solid state (thin-film) cyclic voltammetry (CV) experiments were carried out with a Metrohm Autolab PGSTAT101 Electrochemical Analyser using NOVA software. Number-average (M_n_) and weight-average (M_w_) molecular weights relative to narrow dispersity polystyrene standards (PS) were determined by analytical GPC on an Agilent Technologies 1200 series GPC running chlorobenzene solvent at 80 °C equipped with two PL mixed-B columns in series and refractive index (RI) and UV (254 nm) detectors. Dynamic scanning calorimetry (DSC) was performed on either a Mettler Toledo DSC1 or TA instruments model Q10 DSC at a scan rate of 10 °C min^-1^. Thermogravimetric analysis (TGA) was carried out using a Mettler Toledo TGA/DSC1, heating from 25 to 750 °C at 5 °C min^-1^ under nitrogen. Atomic force microscope (AFM) images were obtained from a Picoscan PicoSPM LE scanning probe in tapping mode. Density function theory (DFT) calculations were modeled using Gaussian 16 at the B3LYP/6-31G(d,p) level of theory and the results were visualized using GaussView 6.

Crosslinking was performed with a low-intensity hand‐held (6 W) UV lamp, set to emit at 254 nm, and held approximately 1 cm from the substrate. Crosslinking was performed in a nitrogen-filled glovebox, and optionally followed by thermal annealing.

**Thin-film transistor (TFTs) fabrication**

Glass substrates were initially cleaned by ultrasonication in dilute Extran 300 detergent solution, followed by DI water for 30 min each. The substrates were then cleaned with acetone and 2-propanol using ultrasonication for 10 min each. Next 35 nm of Au, with 5 nm of Al as adhesion layer were thermally evaporated as source/drain electrodes. The resulting substrates were exposed to UV-ozone treatment for 20 min. To enhance electron injection, a solution of polyethyleneimine (PEIE) in 2-methoxyethanol (0.03 wt%) was deposited on the UV-treated electrodes by spin-coating (5000 rpm for 60 sec). The resulting substrates were thermally annealed at 120 °C for 10 min.

A solution of the conjugated polymer (5 mg mL^-1^ in 1,2-dichlorobenzene) was prepared by stirring overnight at 120 °C. The resulting solution was deposited by spin-coating (1000 rpm, 60 sec). Films were annealed at the indicated temperatures for 30 min. After cooling to room temperature, a solution of polymethylmethacrylate (PMMA) in butyl acetate (80 mg mL^-1^) was deposited by spin-coating (2000 rpm for 60 s) and thermally annealed at 95 °C for 2 h. An Al gate electrode was then deposited as the gate electrode. All fabrication procedures were performed in a nitrogen-filled glove box. Electrical characterisation was performed in a N_2_ glove box using an Agilent B1500A semiconductor parameter analyzer.

**GIWAXS**

Grazing-incidence wide-angle X-ray scattering (GIWAXS) measurements were performed at the BL11 NCD-SWEET at ALBA Synchrotron Radiation Facility (Barcelona, Spain). The incident X-ray beam energy was set to 12.4 eV using a channel cut Si (1 1 1) monochromator. The angle of incidence *α_i_* was set between 0.1° and 0.2° to ensure surface sensitivity. Data are expressed as a function of the scattering vector, which was calibrated using Cr_2_O_3_, obtaining a sample-to-detector distance of 145.6 mm. The scattering patterns were recorded using a Rayonix LX255-HS area detector, which consists of a pixel array of 1920 × 5760 pixels (H × V) with a pixel size of 44 × 44 µm^2^. All the measurements were performed under N_2_ atmosphere to minimize the damage of the films. 2D GIWAXS patterns were corrected as a function of the components of the scattering vector (*q*). Polymer thin films spun were prepared on Si wafer substrates (5 mg mL^-1^ in 1,2-dichlorobenzene; 1000 rpm, 60 sec). Crystal correlation lengths (CCL) were estimated via Scherrer analysis using the following equation:

$${CCL}_{hkl} = \frac{2\pi K}{{FWHM}_{q}}$$

where the FWHM*_q_* is the full width at half maximum of the fitted peak in reciprocal space (*q*), and K = 0.9.

## Experimental

**2,7-bis(2-decyltetradecyl)benzo[lmn][3,8]phenanthroline-1,3,6,8 (2H,7H)-tetraone (NDI) stability tests**

Small molecule NDI was dissolved in a chlorobenzene:DMAc (3/1, v/v) mixture and heated to 110 °C in the presence of base overnight. The bases tested were potassium carbonate (K_2_CO_3_, weak base, weak nucleophile), potassium hydroxide (KOH, strong base, strong nucleophile), 1,8-diazabicyclo[5.4.0]undec-7-ene (DBU, non-nucleophilic base) and potassium tert-butoxide (^t^BuOK, strong base, weak nucleophile). The reaction was repeated with potassium carbonate (K_2_CO_3_) under identical conditions, but heating to 150 °C. Once the reaction time was reached the mixture was precipitated into methanol. If a precipitate formed it was filtered, redissolved into chloroform and washed with water and brine. After washing, the organic phase was dried, and the solvent removed under vacuum to give the final product. If a precipitate did not form, the methanol was removed under vacuum and the material was redissolved into chloroform and washed with water and brine. After washing, the organic phase was dried, and the solvent removed to give the final product. All the final products were characterised by ^1^H NMR and UV-Vis.

**Synthesis of 1-iodo-2-decyltetradecane**

*as prepared according to a modified procedure of Antonio Facchetti et al.*^1^

In a dry 250 mL two neck round bottom flask under nitrogen a solution of 2-decyl-1-tetradecanol (12.05 g, 34.0 mmol), triphenylphosphine (10.60 g, 40.4 mmol), and imidazole (2.80 g, 41.1 mmol) in toluene (100 mL) was cooled to 0 °C. Iodine (9.90 g, 39.0 mmol) was added, and the mixture was stirred at 0 °C for 15 min before being allowed to warm to room temperature. The reaction mixture was stirred for a further 2 h at room temperature followed by addition of sat. aq. Na_2_SO_3_ (10 mL). The organic solvent was concentrated using a rotary evaporator, and the residue was taken up in petroleum ether (250 mL) and washed with water (3 x 200 mL), and brine (180 mL). The organic phase was dried over MgSO_4_ and concentrated under reduced pressure. The residue was purified by column chromatography over silica (eluent: petroleum ether) to give 1-iodo-2-decyltetradecane as a colourless oil (14.2 g, 90% yield).

^1^H NMR (400 MHz, CDCl_3_): δ 3.26 (d, *J* = 4.56 Hz, 2H), 1.40-1.20 (m, 40H), 1.11 (m, 1H) 0.89 (t, *J* = 6.6 Hz, 6H) ppm. ^13^C NMR (101 MHz, CDCl_3_): δ 38.8, 34.6 (2C), 32.1 (2C), 29.9 (2C), 29.8-29.7 (8C), 29.5 (2C), 26.7 (2C), 22.9 (2C), 17.0, 14.3 (2C) ppm. MS (*m/z)* [M]^+^ calcd. for C_24_H_49_I: 463.2795, found: 463.2794 (APCI)

**Synthesis of 2-decyltetradecylamine**

*as prepared according to a modified procedure of Antonio Facchetti et al. and Watson et al.*^1,2^

In a dry 100 mL two neck round bottom flask under nitrogen a solution of 1-iodo-2-decyltetradecane (10.05 g, 21.6 mmol) and phthalimide (4.44 g, 30.2 mmol) in DMF (50 mL) was stirred vigorously for 72 h at 25 °C. The DMF was then removed using a rotary evaporator and the resultant oil was dissolved in petroleum ether (400 mL) and washed with water (3 x 300 mL) and brine (250 mL). The organic phase was dried over MgSO_4_, concentrated under reduced pressure, and passed through a silica plug (eluent: petroleum ether/chloroform 9:1). The resulting oil was dissolved in ethanol (300 mL) in a dry 500 mL two neck round bottom flask filled with nitrogen, hydrazine hydrate solution (10 mL, 80% in H_2_O) was added dropwise, and the mixture was refluxed overnight. After cooling to room temperature, ethanol was removed under reduced pressure and the remaining crude mixture was diluted with DCM (200 mL). The organic phase was washed with 10% aq. KOH (2 x 100 mL), and the resulting aqueous phase was extracted with DCM. The combined organic phases were washed with water (3 x 100 mL) and brine (80 mL), and dried over MgSO_4_. Solvent was removed by rotary evaporator to give 2-decyltetradecylamine as a yellow oil (1.32 g, 17% yield)

^1^H NMR (400 MHz, CDCl_3_): δ 4.44 (br, 2H), 2.61 (d, *J* = 4 Hz, 2H), 1.42 (m, 1H), 1.28 (br, 40H), 0.90 (t, *J* = 8 Hz, 6H) ppm. ^13^C NMR (101 MHz, CDCl_3_): δ 45.2, 40.7, 32.1 (2C), 31.6 (2C), 30.2 (2C), 29.8-29.8 (8C), 29.5 (2C), 26.9 (2C), 22.9 (2C), 14.3 (2C) ppm. MS (*m/z)* [M]^+^ calcd. for C_24_H_52_N: 354.4100, found: 354.4105 (ESI).

**Synthesis of N,N’-bis(2-decyltetradecyl)-2,6-dibromonaphthalene-1,4,5,8-bis(dicarbox-imide) (NDI-Br_2_) monomer**

*as prepared according to a modified procedure of Antonio Facchetti et al.*^3^

To a 20 mL 2-neck round bottom flask under nitrogen, 2,6-dibromonaphthalene-1,4,5,8-tetracarboxydianhydride (NDA-Br_2_) (1.00 g, 2.36 mmol), 2-decyltetradecylamine (2.08 g, 5.88 mmol) in *o*-xylene (8 mL) and propanoic acid (2.6 mL) were added and stirred at 140 °C for 2 h. The reaction was monitored by TLC to check for consumption of the starting material. The mixture was cooled to room temperature before direct purification by column chromatography over silica (eluent: hexane/chloroform 1:2). The solvent was removed under reduced pressure before the product was further purified by recrystallisation with ethyl acetate. The recrystallized product was collected by vacuum filtration through a nylon pad (pore size 20 μm), washed with ice-cold ethyl acetate and left to dry. The dry product was collected in a vial and dried under vacuum to yield the product as a yellow solid. (1.38 g, 55% yield)

^1^H NMR (400 MHz, CDCl_3_): δ 9.00 (s, 2H), 4.14 (d, *J* = 8 Hz, 4H), 1.98 (m, 2H), 1.15-1.40 (m, 80H), 0.83-0.92 (m, 12H) ppm. ^13^C NMR (101 MHz, CDCl_3_): δ 161.3 (2C), 161.2 (2C), 139.3 (2C), 128.5 (2C), 127.9 (2C), 125.4 (2C), 124.2 (2C), 45.6 (2C), 36.6 (2C), 32.1 (4C), 31.7 (4C), 30.2 (4C), 29.8 -29.7 (16C), 29.5 (4C), 26.47 (4C), 22.8 (4C), 14.3 (4C) ppm. MS (*m/z)* [M]^+^ calcd. for C_62_H_100_Br_2_N_2_O_4_: 1093.60, found: 1095.6 (MALDI)

**Synthesis of 2,7-bis(2-octyldodecyl)-4,9-bis((E)-2-(tributylstannyl)vinyl)benzo[lmn][3,8]-phenanthr-oline-1,3,6,8(2H,7H)-tetraone monomer**

*as prepared according to a modified procedure of Zhuping Fei et al.*^4^

To a 100 mL two-neck round bottom flask under nitrogen, NDI-Br_2_ (500 mg, 0.46 mmol), trans-1,2-bis(tributylstannyl)ethene (1.13 g, 1.9 mmol), tris(dibenzylideneacetone)dipalladium(0) (Pd_2_(dba)_3_) (24.3 mg, 0.024 mmol), and tri(o-tolyl)phosphine (P(*o*-tol)_3_) (33 mg, 0.036 mmol) were added and thoroughly degassed by purging with nitrogen for 30 min. Degassed anhydrous toluene (40 mL) was added, and the mixture was degassed for a further 10 min, before the solution was heated to 90 °C and stirred at this temperature overnight. The mixture was allowed to cool to room temperature and passed through silica/potassium fluoride plug (4:1 w:w, eluent: DCM). The solvent was removed under reduced pressure, and the residue was purified by chromatography over silica (eluent: DCM/hexane 1:2). The fractions containing the product were collected and the solvent was removed under reduced pressure. The product was dissolved in a minimum amount of hexane and further purified by preparative GPC (eluent: hexane). The fractions were collected, and the solvent removed to give the product as a yellow oil (277 mg, 38% yield).

^1^H NMR (400 MHz, CDCl_3_): δ 8.93 (s, 2H), 8.39 (d, *J* = 19.2 Hz, 2H), 7.36 (d, *J* = 19.2 Hz, 2H), 4.12 (d, *J* = 7.6 Hz, 4H), 2.04−1.93 (m, 2H), 1.68−1.58 (m, 12H), 1.43−1.17 (m, 92H), 1.08 (m, 12H), 0.93 (t, *J* = 7.2 Hz, 18H), 0.88−0.83 (m, 12H) ppm.

**Synthesis of** **PNDI-TfBTT polymer**

*as prepared according to a modified procedure of Huajie Chen and Yunqi Liu et al.*^5^

To a 20 mL microwave vial under nitrogen, 5-fluoro-4,7-bis(5-(trimethylstannyl)thiophen-2-yl)benzo[c][1,2,5]thiadiazole (450 mg, 0.70 mmol), NDI-Br_2_ (750 mg, 0.70 mmol), Pd_2_(dba)_3_ (22 mg, 0.024 mmol) and P(*o*-tol)_3_ (35 mg, 0.115 mmol) was added and thoroughly degassed. Dry chlorobenzene (12 mL) was added, and the mixture was degassed for a further 10 min before being heated to 120 °C and being stirred at this temperature for 72 h. The mixture was cooled to room temperature, precipitated in methanol and filtered into a Soxhlet thimble. The product was washed (Soxhlet) with methanol, acetone and hexane. The final product was obtained in three fractions: (1) extracted from chloroform, (2) extracted from chlorobenzene, and (3) unextracted material removed from the thimble. After extraction, the solvent was removed using a rotary evaporator from the chloroform and chlorobenzene fractions. A negligible amount of product was collected from the chloroform fraction. The product was dissolved in the minimum amount of chlorobenzene (approx. 10 mL) and precipitated into methanol (approx. 500 mL). The precipitate was collected by vacuum filtration through a nylon pad (pore size 20 μm), washed with methanol and left to dry. The dry product was collected in a vial and further dried under vacuum. The product obtained was a dark blue solid, with the total mass of the chlorobenzene and unextracted material being 768 mg (87% yield). 198 mg (22.6%) yield from the chlorobenzene fraction, and 570 mg (65%) unextracted. Subsequent polymers were synthesized from the insoluble, unextracted fraction, referred to as PNDI-TfBTT-Insol. Number and weight average molecular weight and polydispersity index were determined by GPC from the material collected in the chlorobenzene fraction, referred to as PNDI-TfBTT-CB.

^1^H NMR (500 MHz, 393K, TCE): δ 9.00-8.95 (m, 2H), 8.50 (d, *J* = 2.8 Hz, 1H), 8.30 (d, *J* = 3.2 Hz, 1H), 8.00 (d, *J* = 10 Hz, 1H), 7.60 (m, 1H), 7.56 (m, 1H), 4.22 (br, 4H), 2.14 (br, 2H), 1.30-1.50 (m, 80H), 0.90-0.97 (m, 12H) ppm. ^19^F NMR (471 MHz, 393K, TCE): δ 106.3 (s, 1F) ppm. GPC (80 °C, chlorobenzene, vs PS): M_n_ = 26 kDa, M_w_ = 96 kDa, *Đ* = 3.7.

**Synthesis of PNDIV-TfBTT polymer**

To a 20 mL microwave vial under nitrogen, 4,7-bis(5-bromothiophen-2-yl)-5-fluorobenzo[c][1,2,5]thia-diazole (44.2 mg, 0.093 mmol), 2,7-bis(2-octyldodecyl)-4,9-bis((E)-2-(tributylstannyl)vinyl)benzo[lmn]-[3,8]phenanthroline-1,3,6,8(2H,7H)-tetraone (145.8 mg, 0.093 mmol), Pd_2_(dba)_3_ (2.9 mg, 0.003 mmol) and P(*o*-tol)_3_ (4.6 mg, 0.015 mmol) was added and thoroughly degassed. Toluene (4 mL) was added, and the mixture was degassed for a further 10 min before being heated to 120 °C and stirred at this temperature for 72 h. The mixture was cooled to room temperature, precipitated in methanol and filtered into a Soxhlet thimble. The product was washed (Soxhlet) with methanol, acetone, and hexane. The final product was obtained in three fractions: (1) extracted from chloroform (negligible amount), (2) extracted from chlorobenzene, and (3) unextracted material removed from the thimble (84.9 mg, 69.8% yield). After extraction, the solvent was removed using a rotary evaporator. The product was dissolved in the minimum amount of chlorobenzene (approx. 2 mL) and precipitated into methanol (approx. 100 mL). The precipitate was collected by vacuum filtration through a nylon pad (pore size 20 μm), washed with methanol and left to dry. The dry product was collected in a vial and further dried under vacuum to yield the product as a dark blue solid (14 mg, 11.5% yield). Total combined yield of chlorobenzene and unextracted materials was 99 mg (81%). Subsequent polymers were synthesized from the insoluble unextracted material, termed PNDIV-TfBTT-Insol. Number and weight average molecular weight and polydispersity index were determined by GPC from the material collected in the chlorobenzene fraction, referred to as PNDI-TfBTT-CB.

^1^H NMR (500 MHz, 393K, TCE): δ 9.06-7.48 (br, 7H), 7.22-6.33 (br, 4H), 4.30 (br, 4H), 2.02 (br, 2H), 1.10-1.40 (m, 92H), 0.90-1.00 (br, 15H) ppm. ^19^F NMR (471 MHz, 393K, TCE): δ 106.0 (br, 1F) ppm. GPC (80 °C, chlorobenzene, vs PS): M_n_ = 43 kDa, M_w_ = 89 kDa, *Đ* = 2.1.

**Synthesis of PNDI-T(SR)BTT polymer**

To a 2-5 mL microwave vial under nitrogen, PNDI-TfBTT-Insol (10 mg, 0.008 mmol) and an excess of K_2_CO_3_ (13.8 mg, 0.10 mmol) were added. The vial was sealed with a septum and degassed with nitrogen before anhydrous chlorobenzene (1.5 mL) and DMF (0.5 mL) (concentration of 5 mg mL^-1^ of polymer) were added. Excess 1-octanethiol (4 mg, 0.027 mmol) was then added and the solution was degassed with nitrogen. The solution was heated in a microwave reactor at 150 °C for 30 min. After cooling, the solution was precipitated into methanol (10:1 v:v of methanol:solution), stirred for 30 min and filtered through a Soxhlet thimble. Unreacted thiol was removed by washing (Soxhlet) with acetone and the polymer was extracted with CHCl_3_. The CHCl_3_ fraction was concentrated to 1 mL, precipitated into MeOH (10:1 v:v of methanol:solution) and filtered to isolate the polymer as a dark blue solid (6.94 mg, 62% yield).

^1^H NMR (500 MHz, 393K, TCE): δ 9.00-8.85 (br, 2H), 8.32-7.78 (m, 3H), 7.58-7.39 (br, 2H), 4.13 (br, 4H), 3.13 (br, 2H), 1.22-1.40 (m, 80H), 0.90-1.00 (br, 12H) ppm. ^19^F NMR (471 MHz, 393K, TCE): No signal observed. GPC (80 °C, chlorobenzene, vs PS): M_n_ = 29 kDa, M_w_ = 64 kDa, *Đ* = 2.2.

**Synthesis of PNDIV-T(SR)BTT polymer**

To a 2-5 mL microwave vial under nitrogen, PNDIV-TfBTT-Insol (10 mg, 0.008 mmol) and an excess of K_2_CO_3_ (13.8 mg, 0.10 mmol) were added. The vial was sealed with a septum and degassed with nitrogen, before anhydrous chlorobenzene (1.5 mL) and DMF (0.5 mL) (concentration of 5 mg mL^-1^ of polymer) were added. Excess 1-octanethiol (4 mg, 0.027 mmol) was then added and the solution was degassed with nitrogen. The solution was heated in a microwave reactor at 150 °C for 30 min. After cooling, the solution was precipitated into methanol (10:1 v:v of methanol:solution), stirred for 30 min and filtered through a Soxhlet thimble. Unreacted thiol was removed by washing (Soxhlet) with acetone and the polymer was extracted with CHCl_3_. The CHCl_3_ fraction was concentrated to 1 mL, precipitated into MeOH (10:1 v:v of methanol:solution) and filtered to isolate the polymer as a dark blue solid (5.49 mg, 48% yield).

^1^H NMR (500 MHz, 393K, TCE): δ 9.23-7.40 (br, 7H), 7.25-6.35 (br, 4H), 4.29 (br, 4H), 3.18 (br, 2H), 1.40-1.22 (m, 92H), 0.90-1.00 (br, 15H) ppm. ^19^F NMR (471 MHz, 393K, TCE): No signal observed. GPC (80 °C, chlorobenzene, vs PS): M_n_ = 41 kDa, M_w_ = 90 kDa, *Đ* = 2.2.

**Synthesis of PNDI-T(SAz)BTT - 10% polymer**

To a 10 mL microwave vial under nitrogen, pyrrolidine (6.9 mg, 8.1µL, 0.097 mmol) was added to a solution of S-(3-azidopropyl)thioacetate (18.3 mg, 16.04 µL, 0.115 mmol) in DMF (10 mL) and stirred at room temperature for 70 min to obtain 3-azidopropane-1-thiol in situ. To a separate 20 mL microwave vial under nitrogen, PNDI-TfBTT-Insol (60 mg, 0.048 mmol) and an excess of K_2_CO_3_ (33 mg, 0.24 mmol) were added. The vial was sealed with a septum and degassed with nitrogen, before anhydrous chlorobenzene (4.5 mL) and DMF (1.0 mL) (final concentration of 10 mg mL^-1^ of polymer) were added. The mixture containing 3-azidopropane-1-thiol (0.50 mL, 0.0048 mmol) was then added and the solution was degassed with nitrogen. The solution was heated in an oil bath at 120 °C for 12 h. After cooling, the solution was precipitated into methanol (10:1 v:v of methanol:solution), stirred for 30 min and filtered through a Soxhlet thimble. Unreacted thiol was removed by washing (Soxhlet) with methanol and the polymer was extracted with CHCl_3_. The CHCl_3_ fraction was concentrated to 1 mL, precipitated into methanol (10:1 v:v of methanol:solution) and filtered to isolate the polymer as a dark blue solid (52 mg, 85% yield).

^1^H NMR (500 MHz, 393K, TCE): δ 9.00-8.90 (m, 2H), 8.50 (s, 1H), 8.30 (s, 1H), 8.05-7.75 (m, 1H), 7.61-7.47 (br, 2H), 4.35-4.00 (br, 4H), 3.60-3.45 (br, 0.3H), 3.30-3.20 (br, 0.3H), 1.40-1.22 (m, 80H), 0.90-1.00 (br, 12H) ppm. ^19^F NMR (471 MHz, 393K, TCE): δ 106.3 (br, 1F) ppm. GPC (80 °C, chlorobenzene, vs PS): M_n_ = 100 kDa, M_w_ = 251 kDa, *Đ* = 2.5; analysis affected by polymer aggregation.

**Synthesis of PNDI-T(SAz)BTT - 100% polymer**

To a 10 mL microwave vial under nitrogen pyrrolidine (68.2 mg, 81 µL, 0.96 mmol) was added to a solution of S-(3-azidopropyl)thioacetate (184 mg, 161 µL, 0.1.16 mmol) in DMF (10 mL) and stirred at room temperature for 70 min to obtain 3-azidopropane-1-thiol in situ. To a separate 20 mL microwave vial under nitrogen, PNDI-TfBTT-Insol (60 mg, 0.048 mmol) and an excess of K_2_CO_3_ (33 mg, 0.24 mmol) were added. The vial was sealed with a septum and degassed with nitrogen, before anhydrous chlorobenzene (4.5 mL) and DMF (1.0 mL) (final concentration of 10 mg mL^-1^ of polymer) were added. The mixture containing 3-azidopropane-1-thiol (0.50 mL, 0.048 mmol) was then added and the solution was degassed with nitrogen. The solution was heated in an oil bath at 120 °C for 12 h. After cooling, the solution was precipitated into methanol (10:1 v:v of methanol:solution), stirred for 30 min and filtered through a Soxhlet thimble. Unreacted thiol was removed by washing (Soxhlet) with methanol and the polymer was extracted with CHCl_3_. The CHCl_3_ fraction was concentrated to 1 mL, precipitated into MeOH (10:1 v:v of methanol:solution) and filtered to isolate the polymer as a dark blue solid (45 mg, 69% yield).

^1^H NMR (500 MHz, 393K, TCE): δ 9.05-8.85 (br, 2H), 8.40-7.75 (m, 3H), 7.65-7.45 (br, 2H), 4.40-4.00 (br, 4H), 3.60-3.45 (br, 2H), 3.32-3.20 (br, 2H), 1.40-1.22 (m, 82H), 1.00-0.85 (br, 12H) ppm. ^19^F NMR (471 MHz, 393K, TCE): 106.3. GPC (80 °C, chlorobenzene, vs PS): M_n_ = 23 kDa, M_w_ = 66 kDa, *Đ* = 2.8; analysis affected by polymer aggregation.


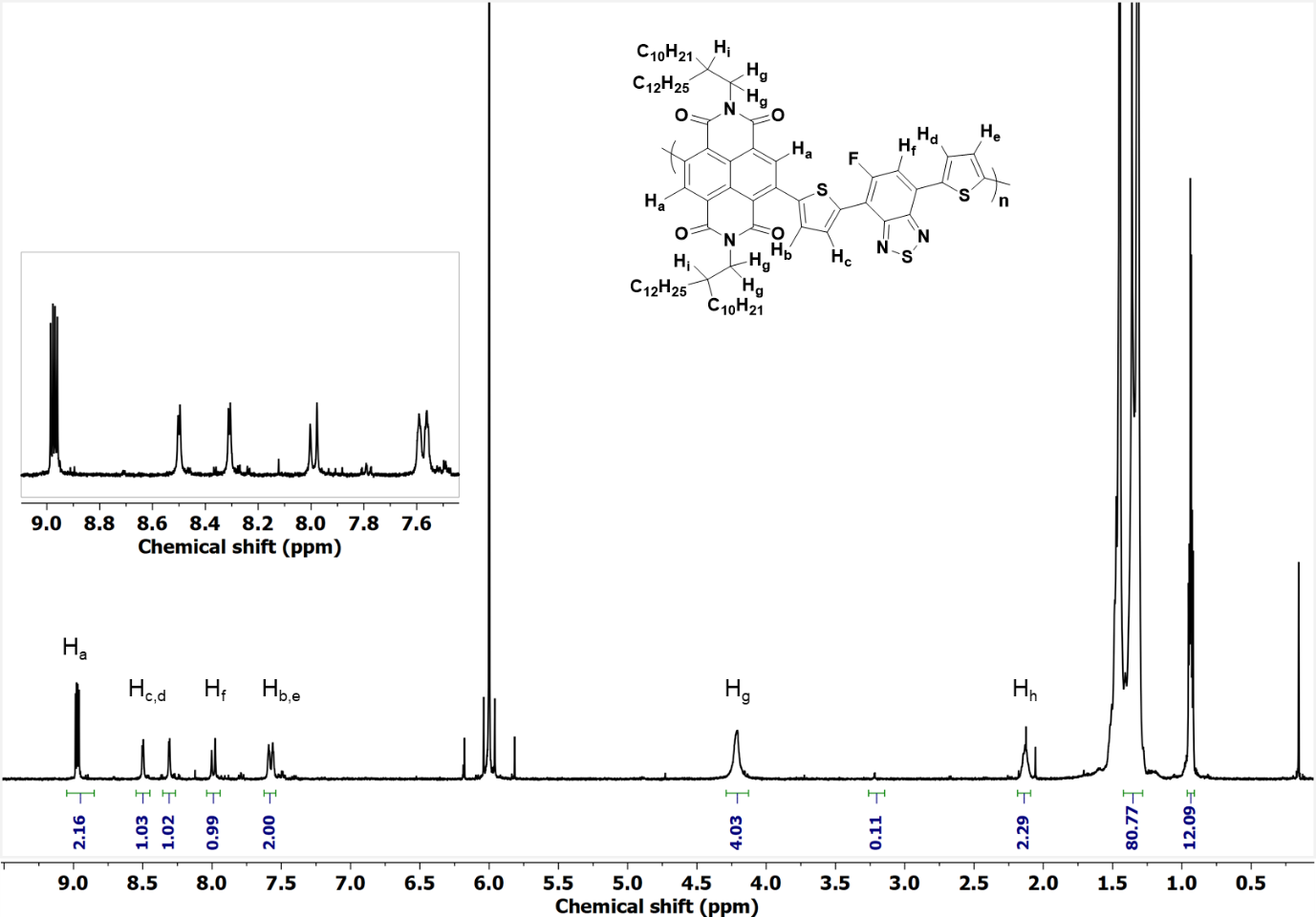


**Figure S1.** ^1^H NMR of PNDI-TfBTT in TCE-d.


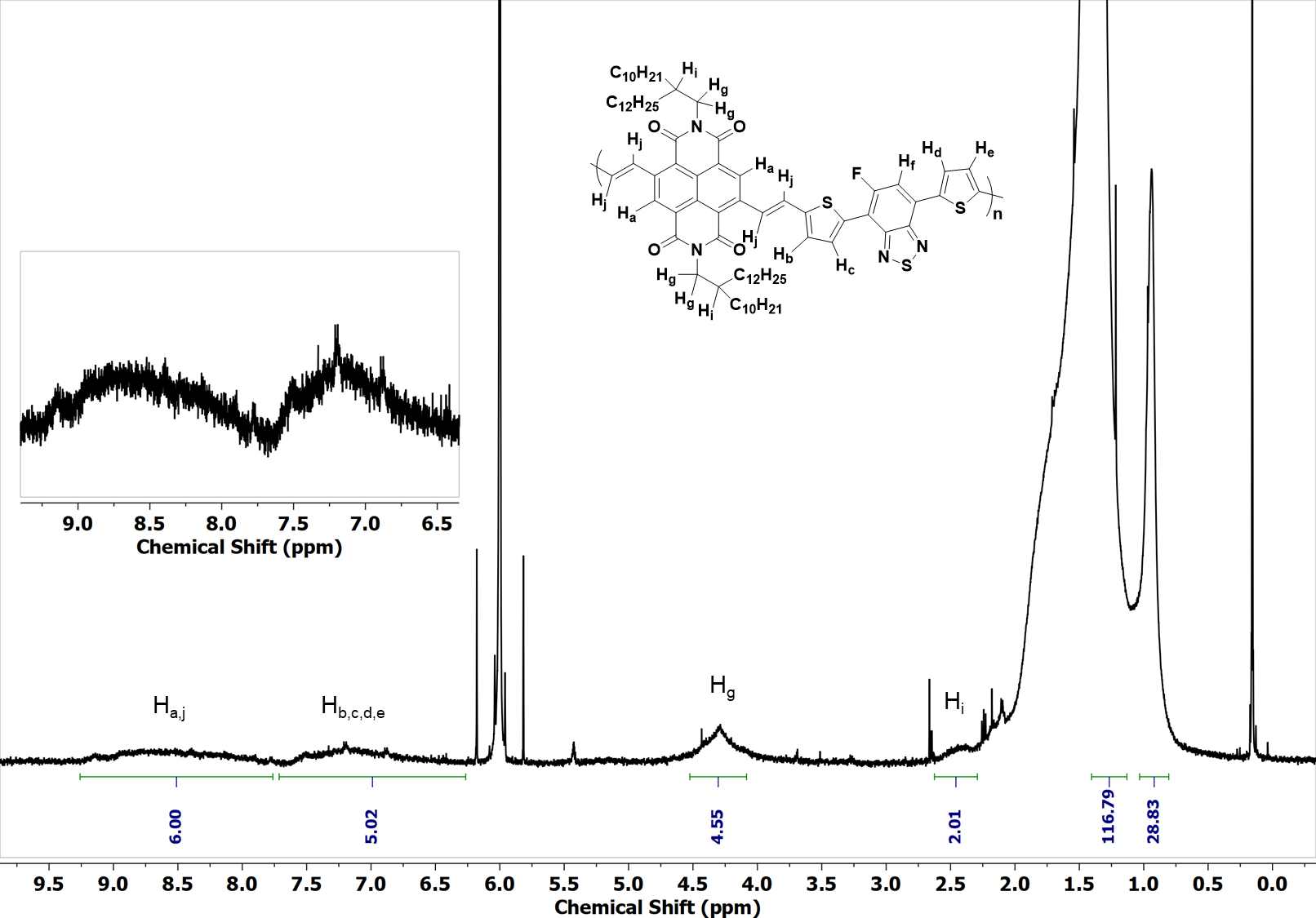


**Figure S2.** ^1^H NMR of PNDIV-TfBTT in TCE-d.

**
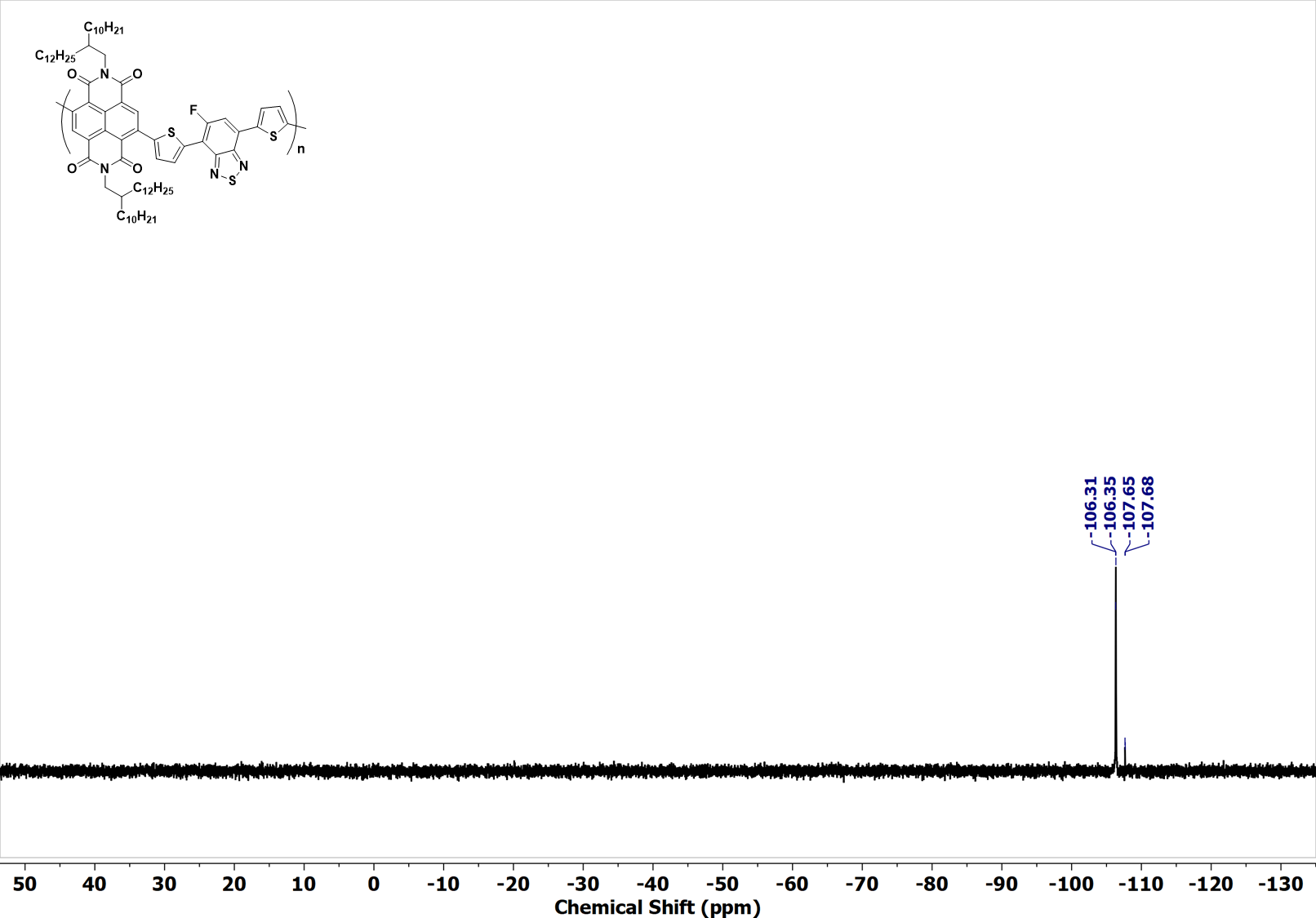
**

**Figure S3.** ^19^F NMR of PNDI-TfBTT. Baseline spline corrected.

**
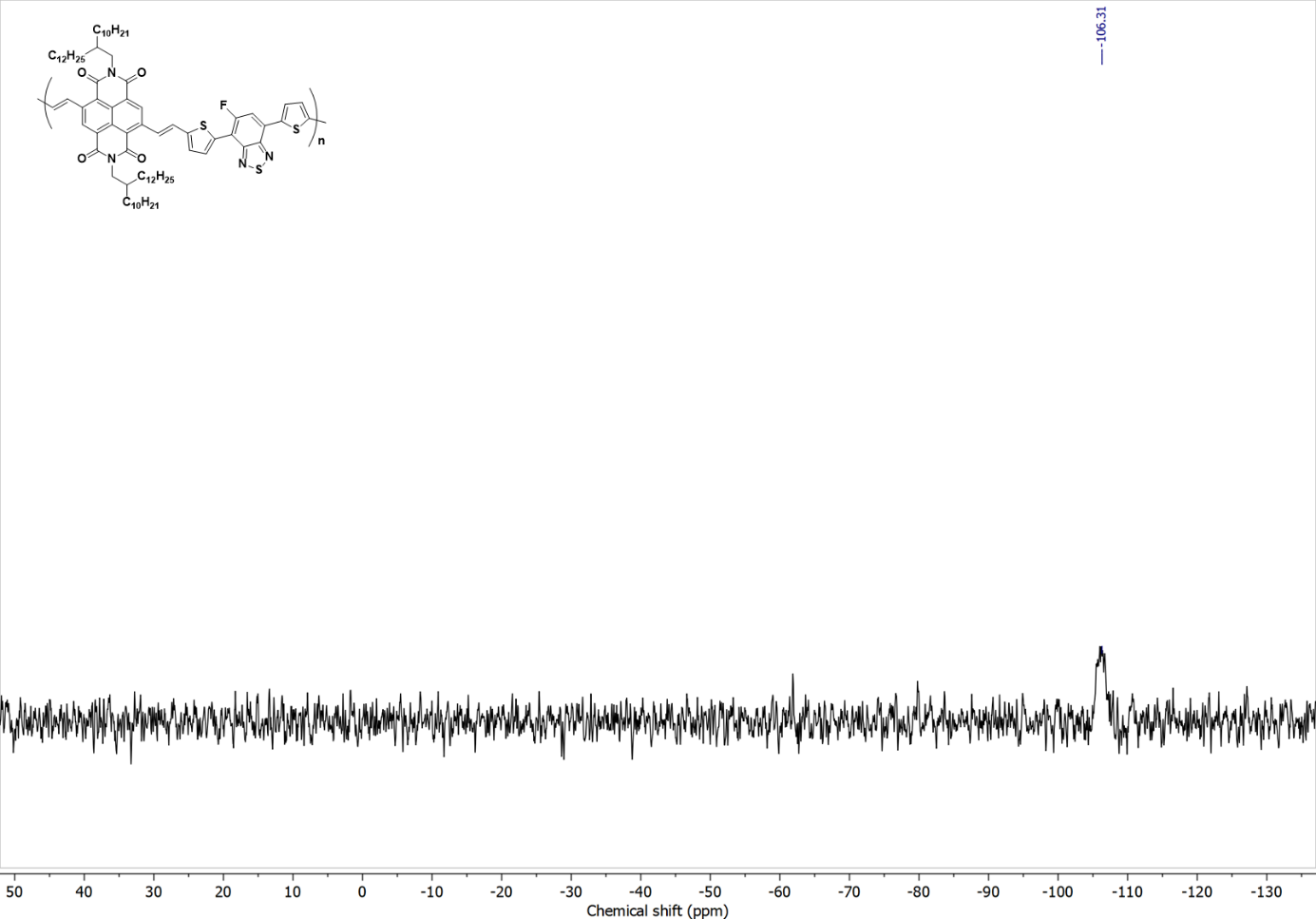
Figure S4.** ^19^F NMR of PNDIV-TfBTT. Baseline spline corrected.

**Table S1.** GPC data for PNDI-TfBTT, PNDIV-TfBTT, PNDI-T(SR)BTT, and PNDIV-T(SR)BTT in chlorobenzene at 80 °C.

| **Polymer** | **M_n_ (kDa)** | **M_w_ (kDa)** | ***Đ*** |
| --- | --- | --- | --- |
| PNDI-TfBTT | 26 | 96 | 3.7 |
| PNDIV-TfBTT | 43 | 89 | 2.1 |
| PNDI-T(SR)BTT | 29 | 64 | 2.2 |
| PNDIV-T(SR)BTT | 41 | 90 | 2.2 |


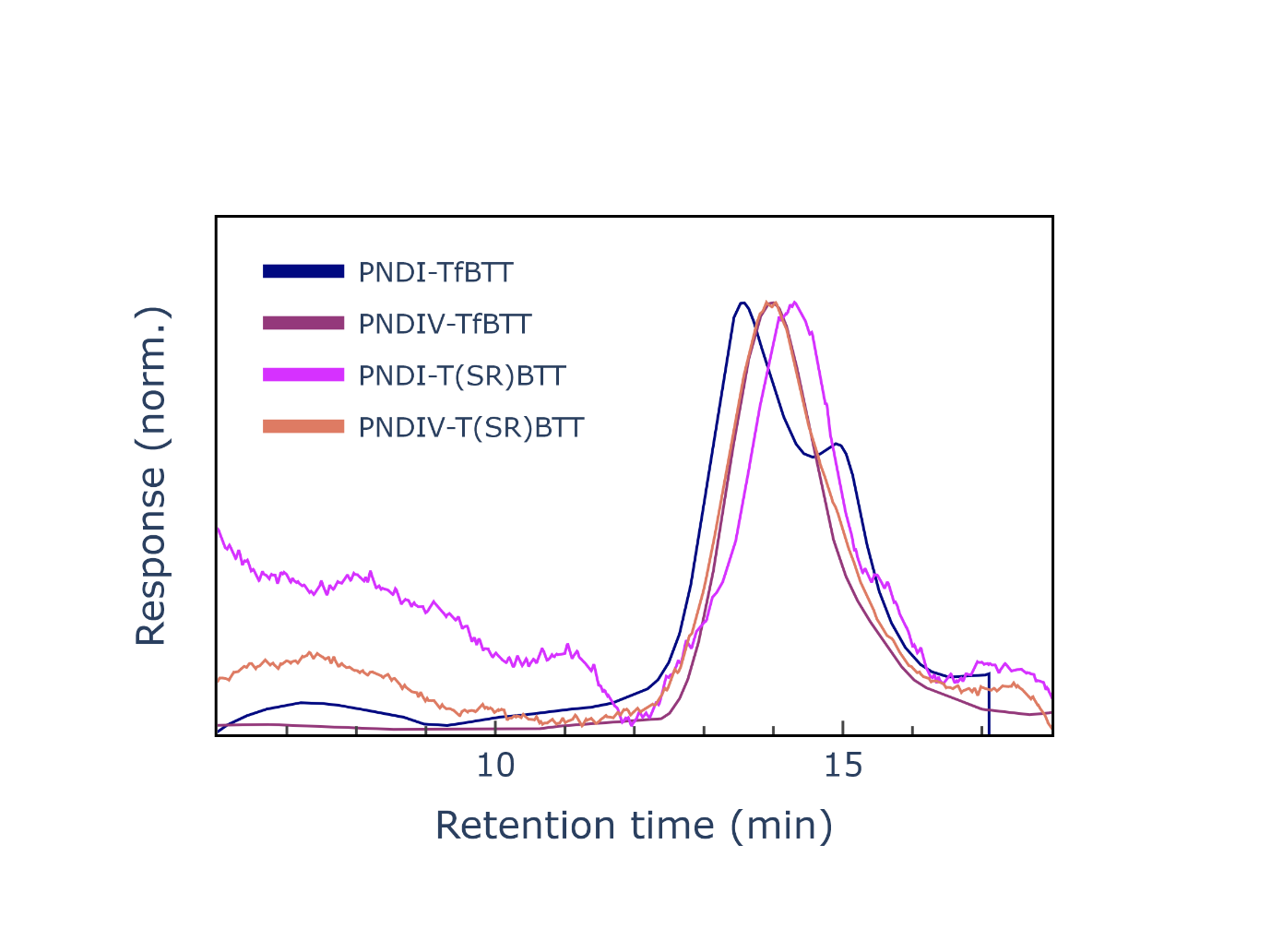


**Figure S5.** GPC traces for PNDI-TfBTT, PNDIV-TfBTT, PNDI-T(SR)BTT, and PNDIV-T(SR)BTT in chlorobenzene at 80 °C.


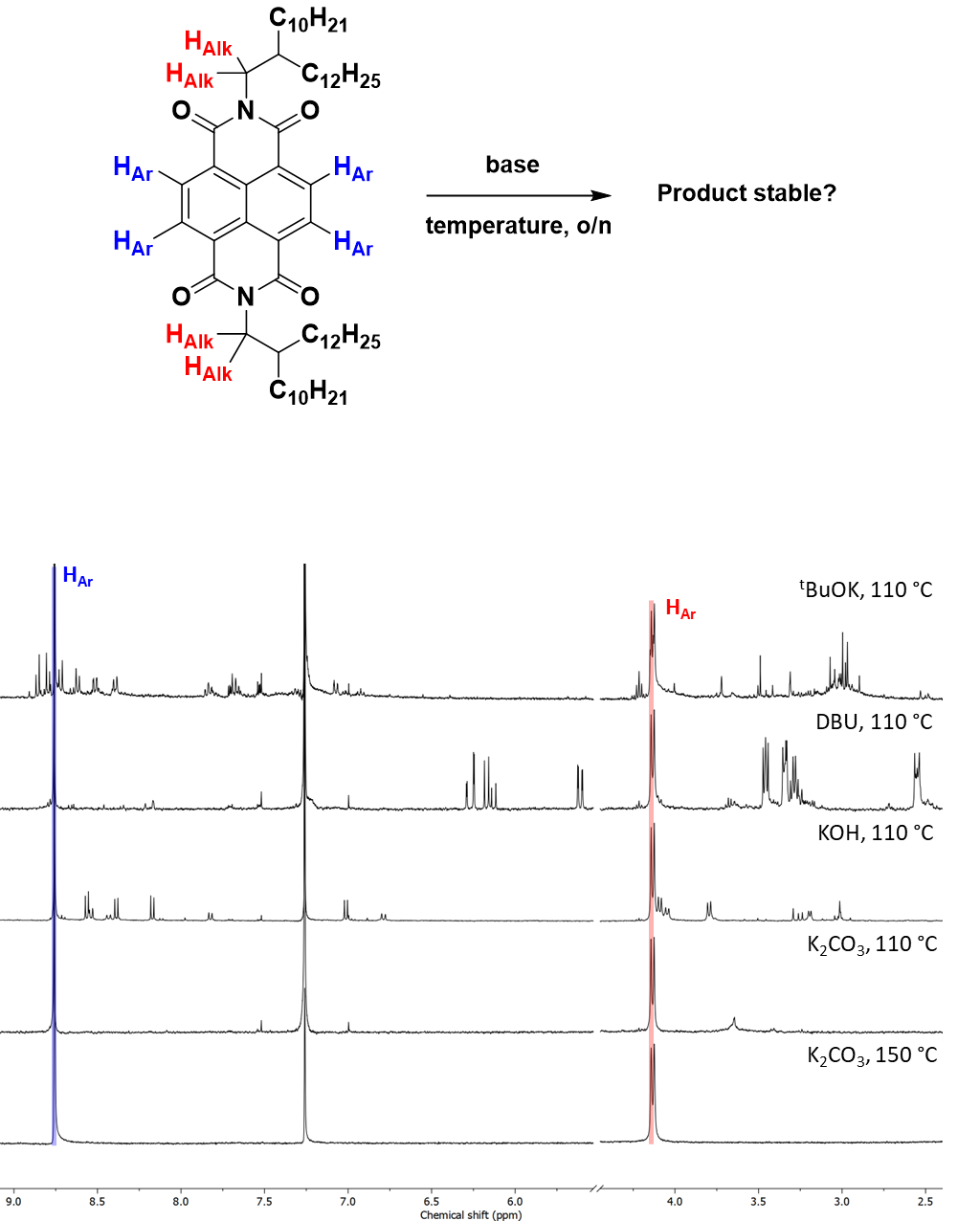


**Figure S6.** ^1^H NMR data of the products from the reaction of the alkylated NDI monomer at 110 °C in the presence of ^t^BuOK, DBU, KOH, and K_2_CO_3_, and at 150 °C in the presence of K_2_CO_3_.


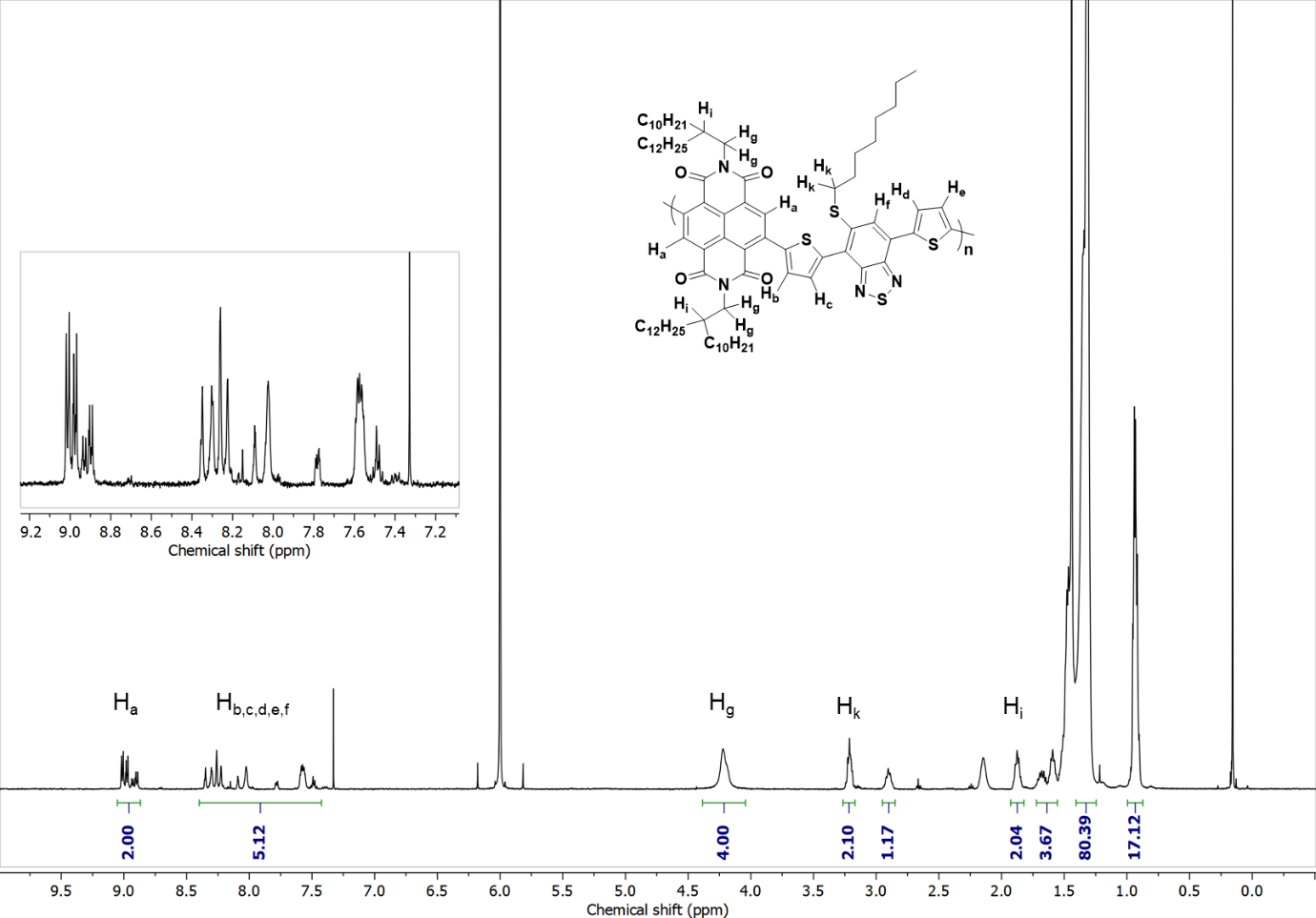
**Figure S7.** ^1^H NMR of PNDI-T(SR)BTT in TCE-d

**
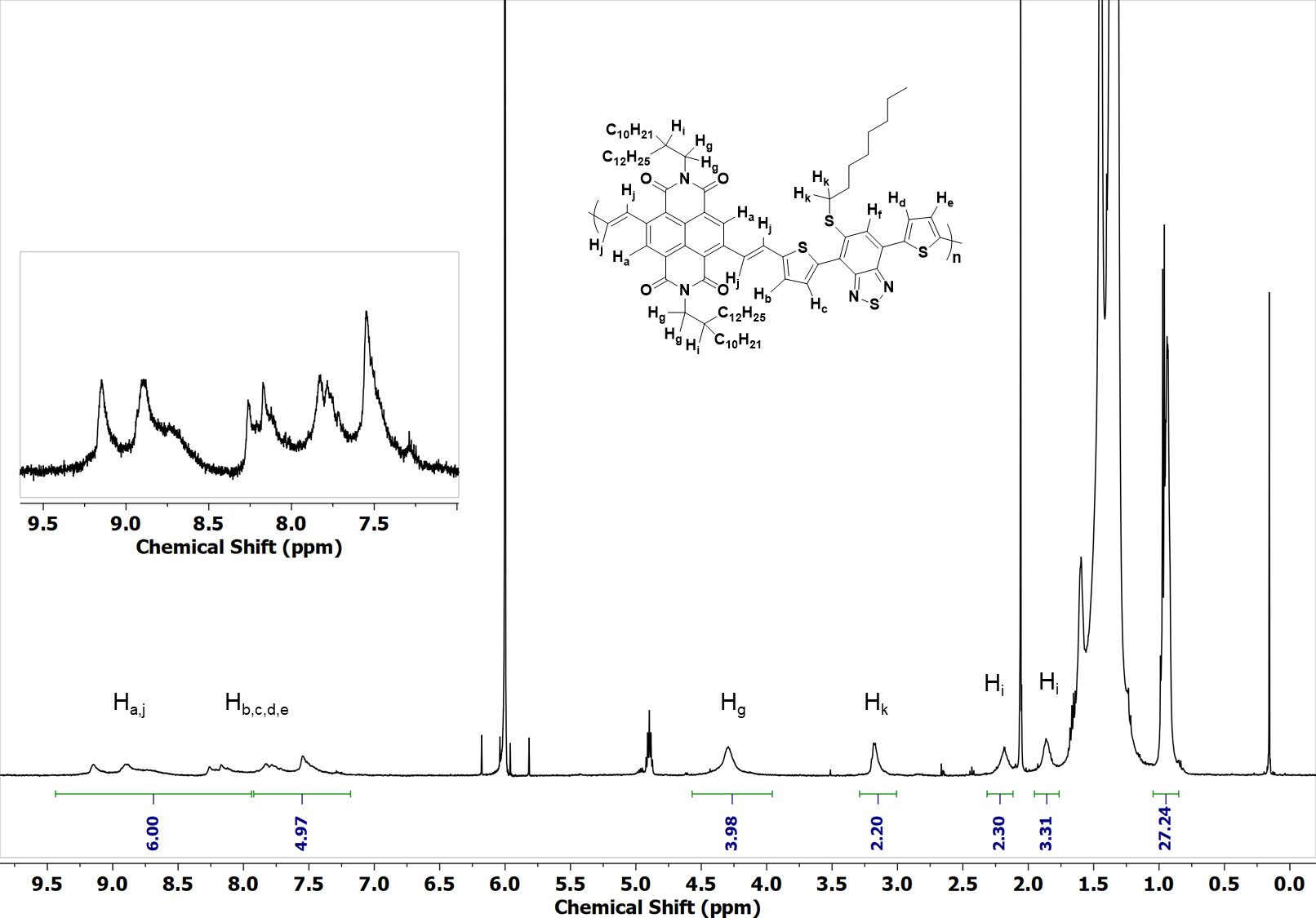
Figure S8.** ^1^H NMR of PNDIV-T(SR)BTT in TCE-d.

**
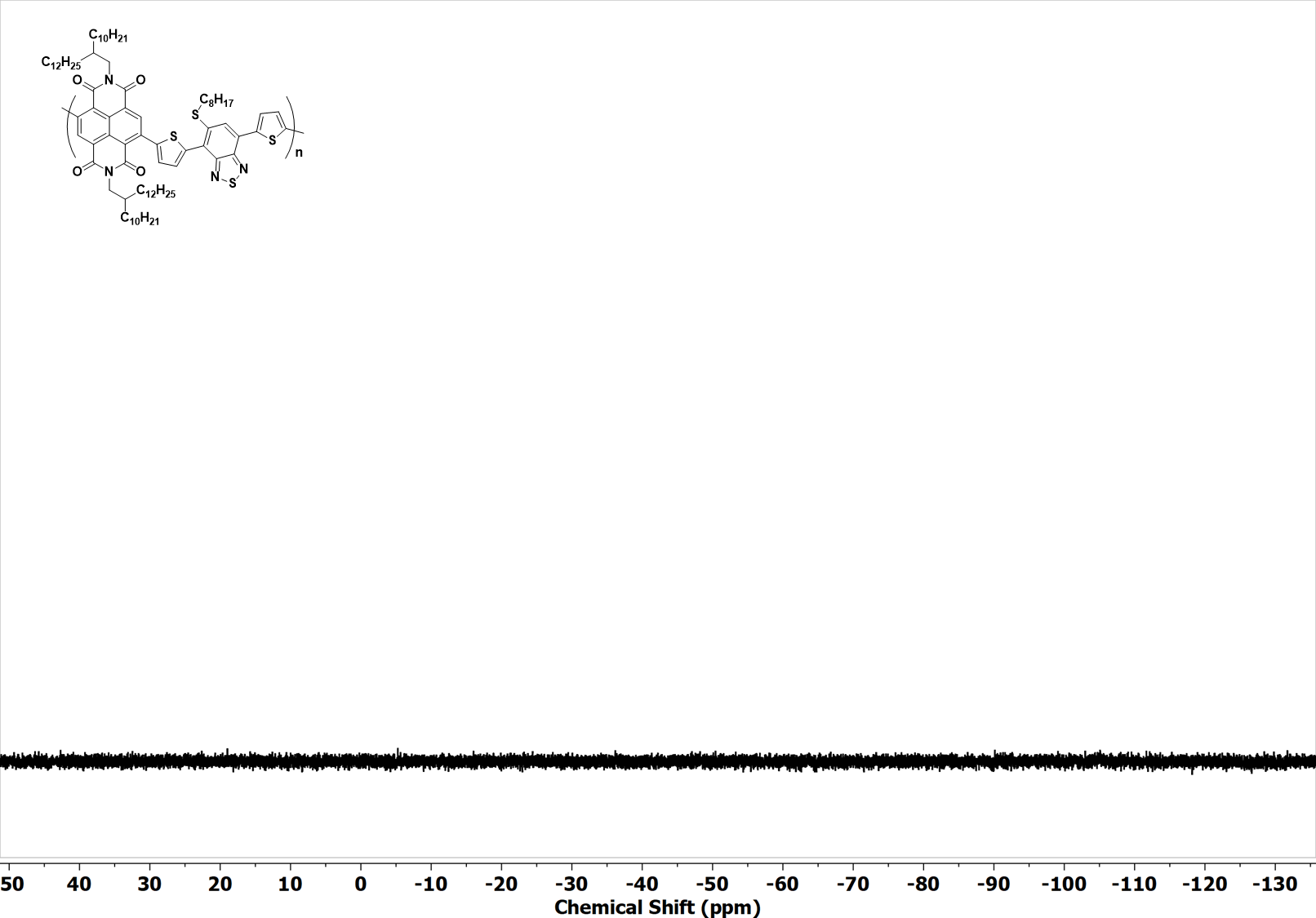
Figure S9.** ^19^F NMR of PNDI-T(SR)BTT. Baseline spline corrected.


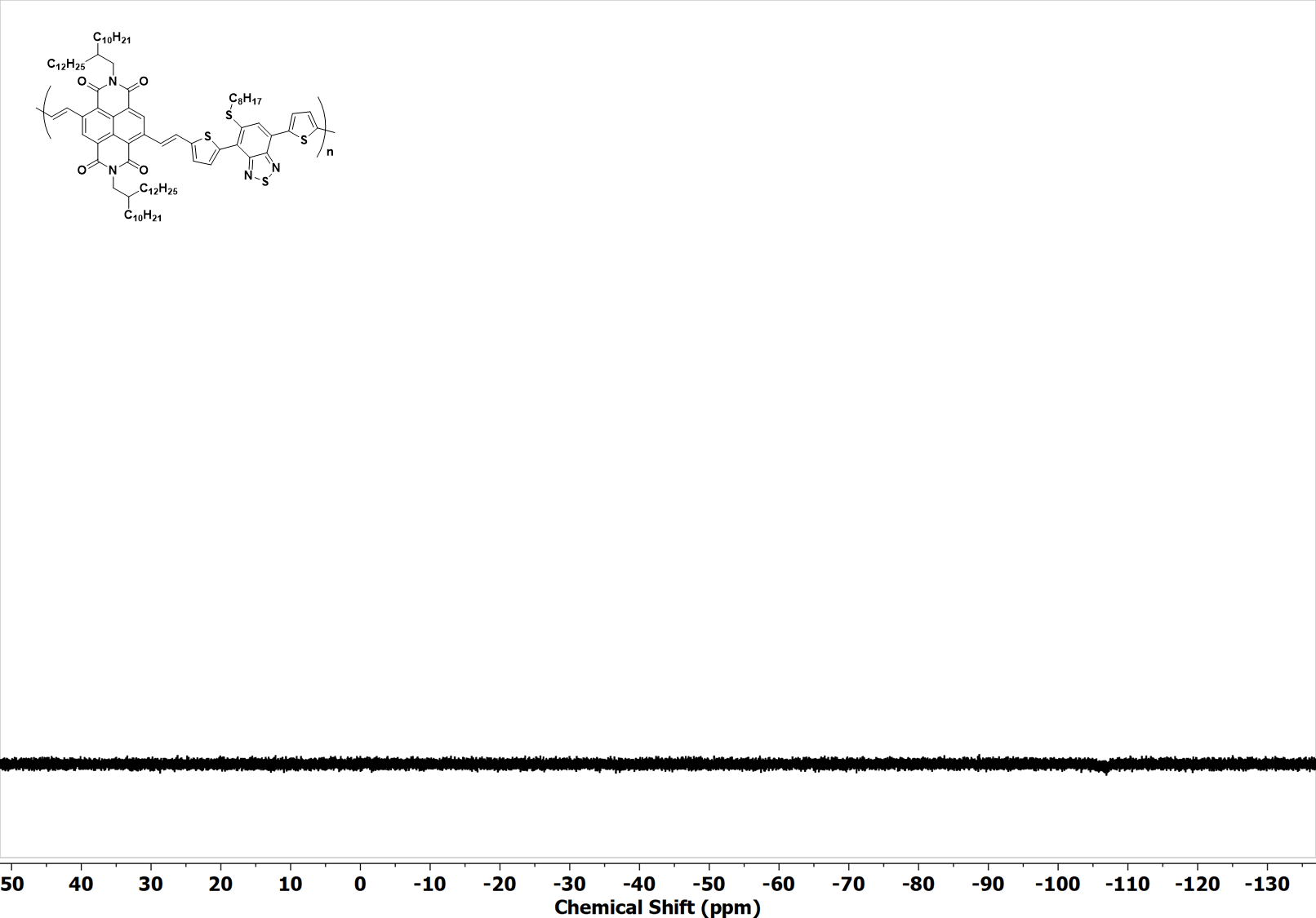
**Figure S10.** ^19^F NMR of PNDIV-T(SR)BTT. Baseline spline corrected.

**Table S2.** Summary of the dihedral angles within energy minimised structures of PNDI-TfBTT, PNDIV-TfBTT, PNDI-T(SR)BTT and PNDIV-T(SR)BTT calculated using DFT at the B3LYP/6-31G* level.

|  | |
| --- | --- |
|  | **Dihedral angle (L→R)** |
| **PNDI-TfBTT** | [1]-41.98, [2] -47.64  [3] 10.03, [4] 7.87  [5] 4.13, [6] 2.50  [7] -41.29, [8] -46.80 |
| **PNDI-T(SR)BTT** | [1] -41.99, [2] -48.29  [3] 16.98, [4] 14.38  [5] 41.79, [6] 39.39  [7] -40.82, [8] -47.88 |
| **PNDIV-TfBTT** | [1] 18.80, [2] -0.51  [3] 0.38, [4] 0.35  [5] 0.25, [6] 0.25  [7] -0.35, [8] 18.08 |
| **PNDIV-T(SR)BTT** | [1] 18.45, [2] 0.38  [3] -5.11, [4] -4.77  [5] 38.27, [6] 37.53  [7] -4.33, [8] 19.88 |

**Figure S11.** ^19^F NMR spectra of PNDI-TfBTT as a function of increasing octanethiol substitution along the backbone.

**Table S3.** Expected and measured integrations of proton environment H_k_ relative to H_a_ (set to 2H).

| **mol% of thiol added** | **XH_k_**  **(expected)** | **XH_k_**  **(measured)** |
| --- | --- | --- |
| 0.00 | 0.00 | 0.11 |
| 0.20 | 0.40 | 0.62 |
| 0.40 | 0.80 | 0.87 |
| 0.60 | 1.20 | 1.22 |
| 0.80 | 1.60 | 1.86 |
| 1.00 | 2.00 | 2.18 |

Deviation of the measured values from the expected values in ^1^H NMR could be related to aggregation of the polymer in solution. ^1^H NMR spectra were measured at high temperature (393 K), due to the low solubility of the polymer, but even at high temperature aromatic and aliphatic signals could be affected by aggregation of the polymer.

| **Table S4.** Optical and electronic properties for PNDI-TfBTT, PNDIV-TfBTT, PNDI-T(SR)BTT, and PNDIV-T(SR)BTT in solution and film states. | | | | | |
| --- | --- | --- | --- | --- | --- |
| **Polymer** | **λ_abs,max_ (nm), solution** | **λ_abs,max_ (nm), film** | **λ_onset_ (nm), solution** | **λ_onset_ (nm), film** | **E_g,opt_^a^ (eV)** |
| **PNDI-TfBTT** | 365, 485, 666, 710 | 485, 660, 695 | 773 | 773 | 1.60 |
| **PNDIV-TfBTT** | 325, 400, 710 | 392, 680, 724 | 819 | 812 | 1.53 |
| **PNDI-T(SR)BTT** | 340, 385, 460, 575 | 468, 650 | 685 | 770 | 1.61 |
| **PNDIV-T(SR)BTT** | 390, 665 | 390, 680 | 809 | 822 | 1.50 |
| ^a^Estimated from the onset of absorption from the measured thin film UV-Vis absorption spectra where E = hc/λ | | | | | |

**
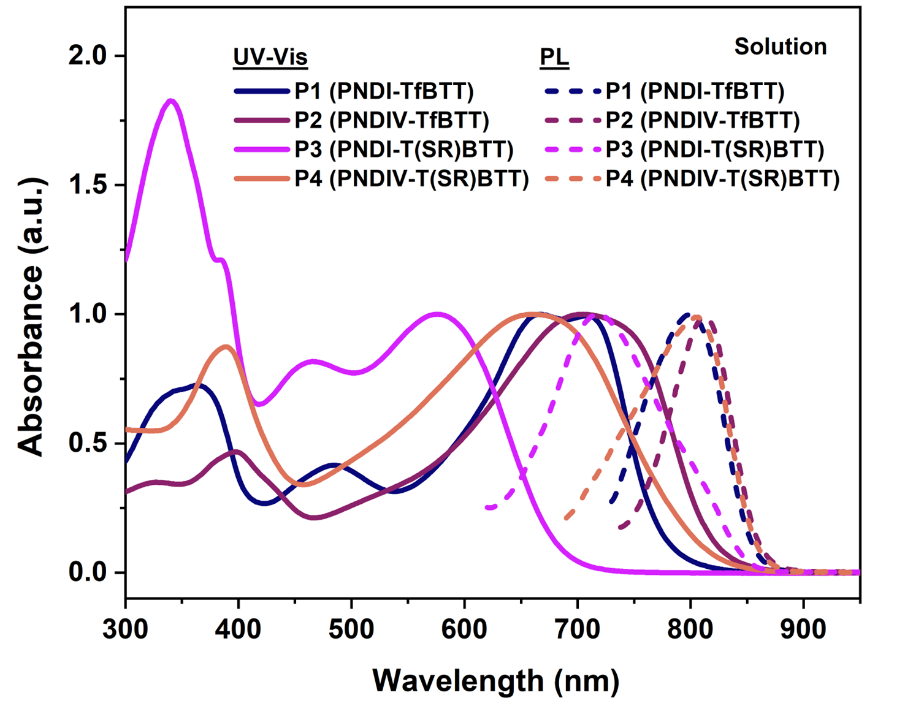
**

**Figure S12.** UV-Vis absorption and PL emission spectra for PNDI-TfBTT, PNDIV-TfBTT, PNDI-T(SR)BTT, and PNDIV-T(SR)BTT in chlorobenzene solution.

**PNDI-TfBTT**

**PNDIV-TfBTT**


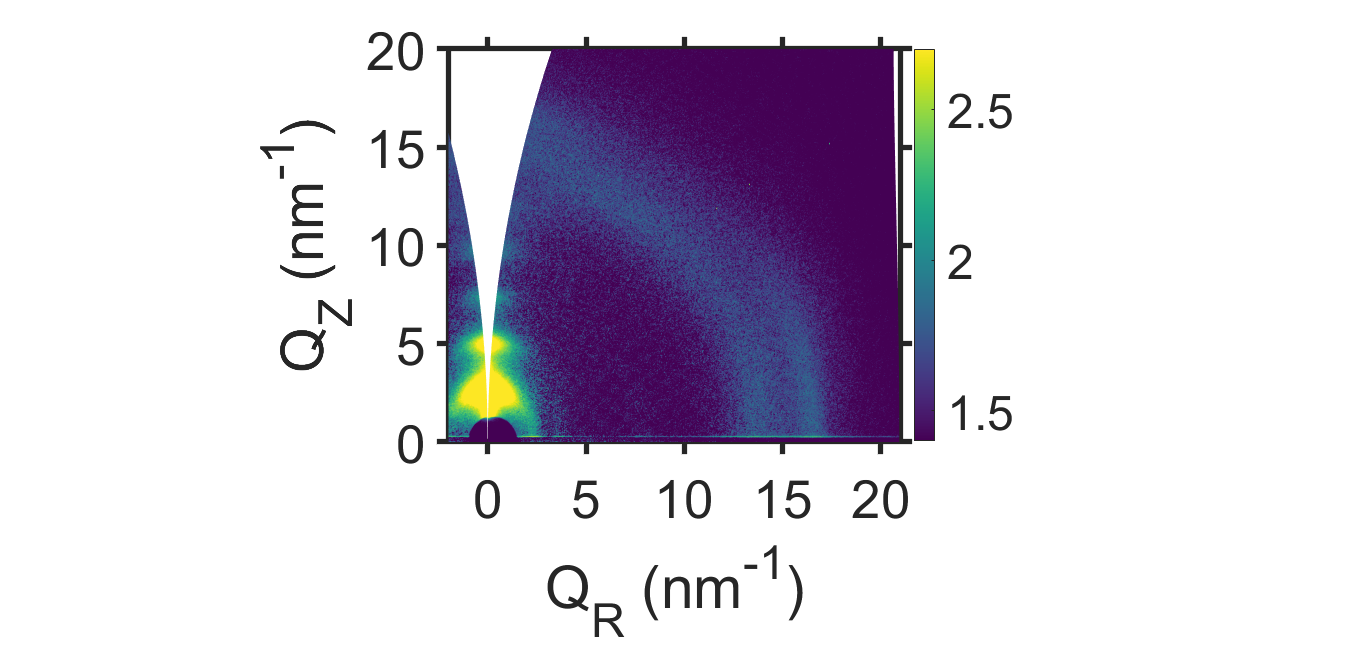

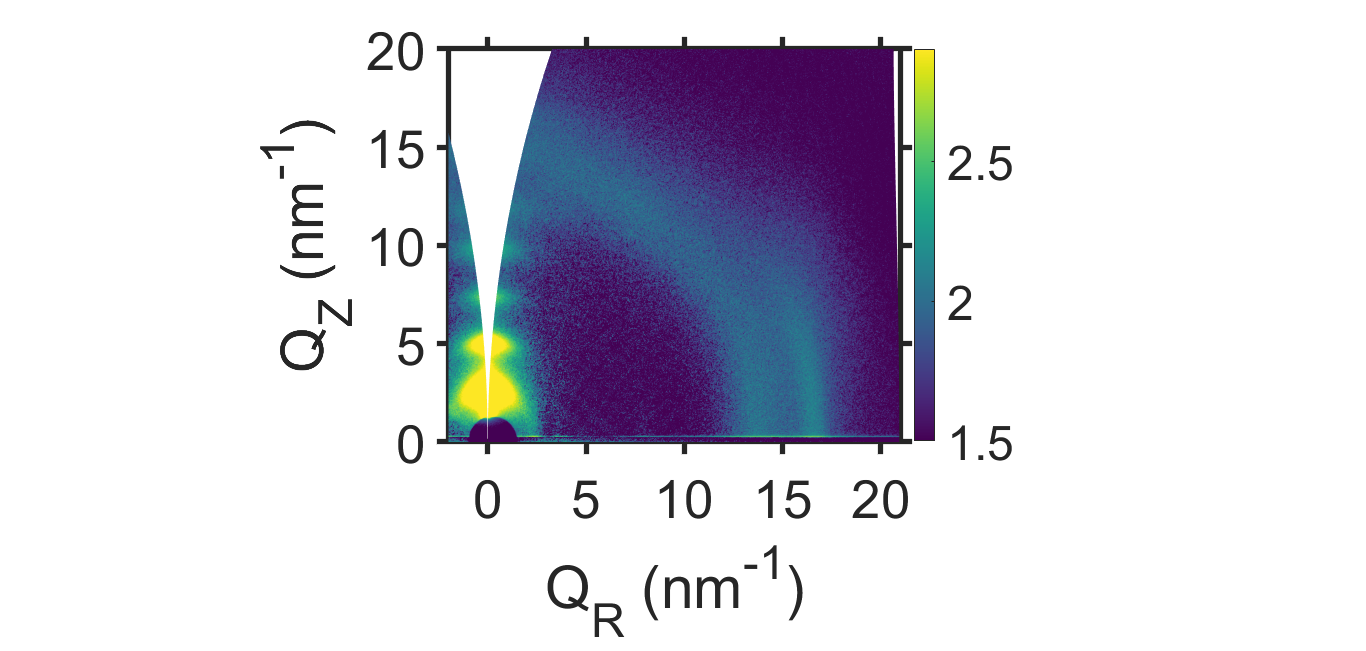


**as-cast**


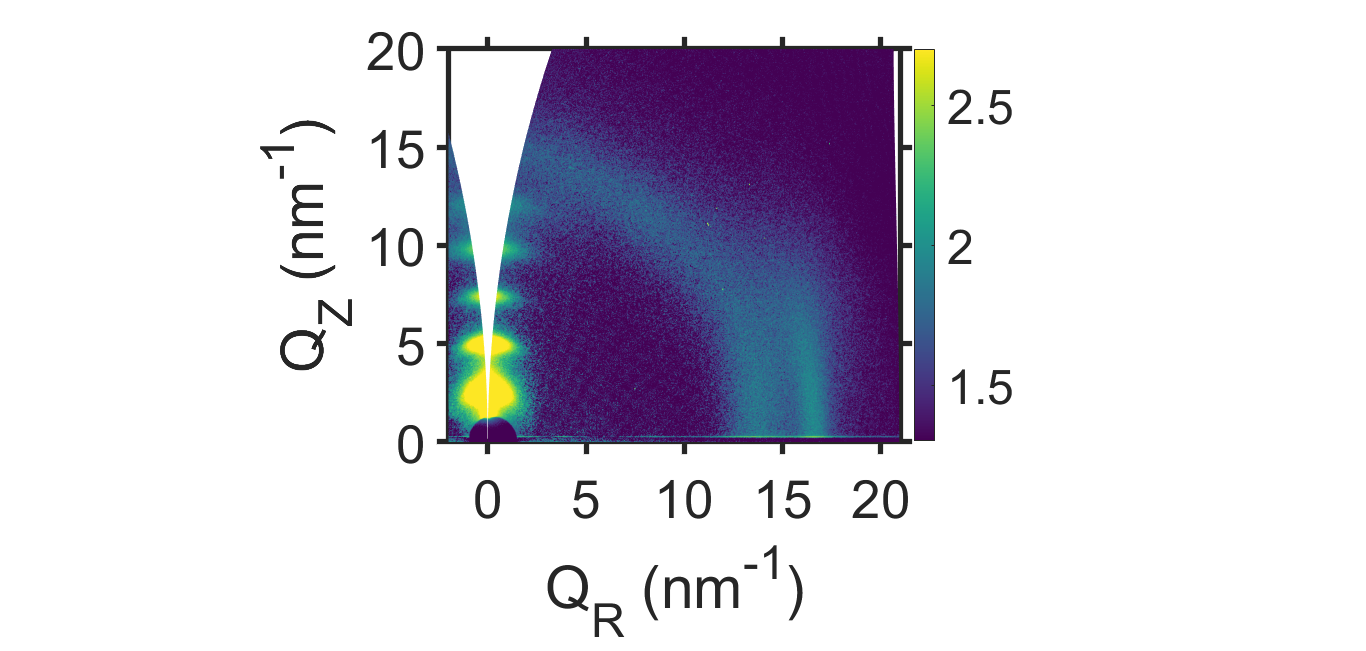


**200°C**

**100°C**


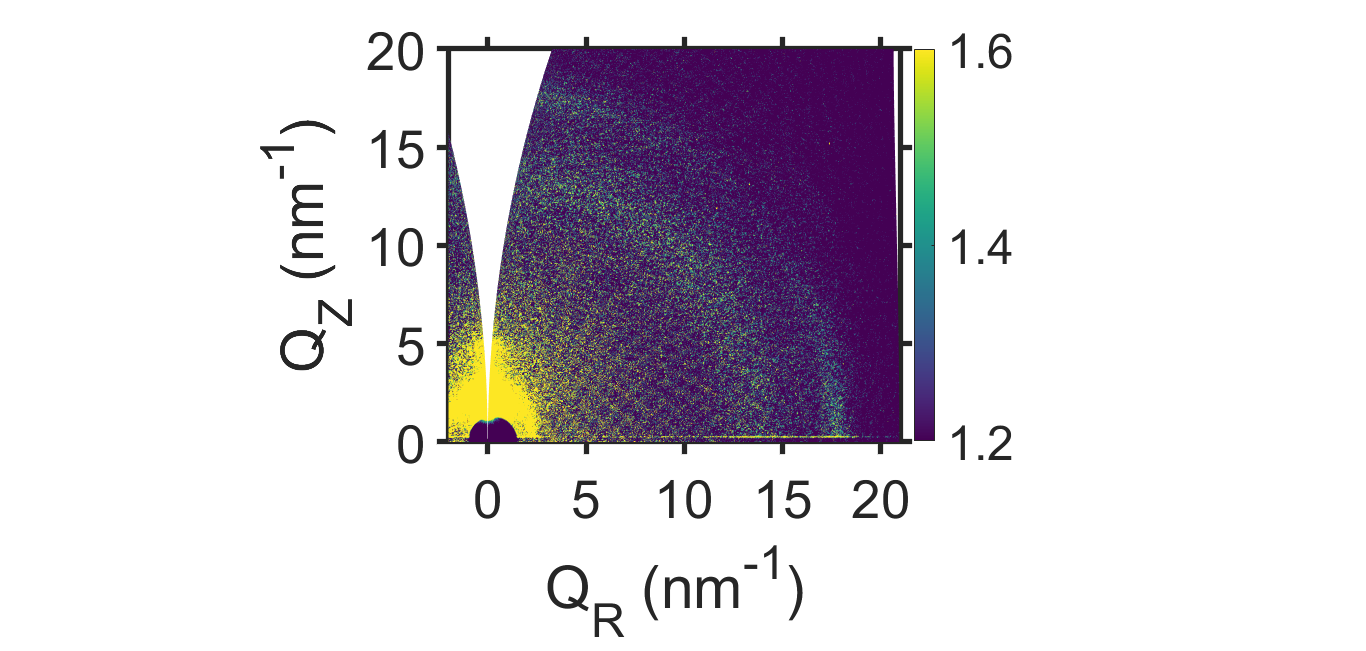

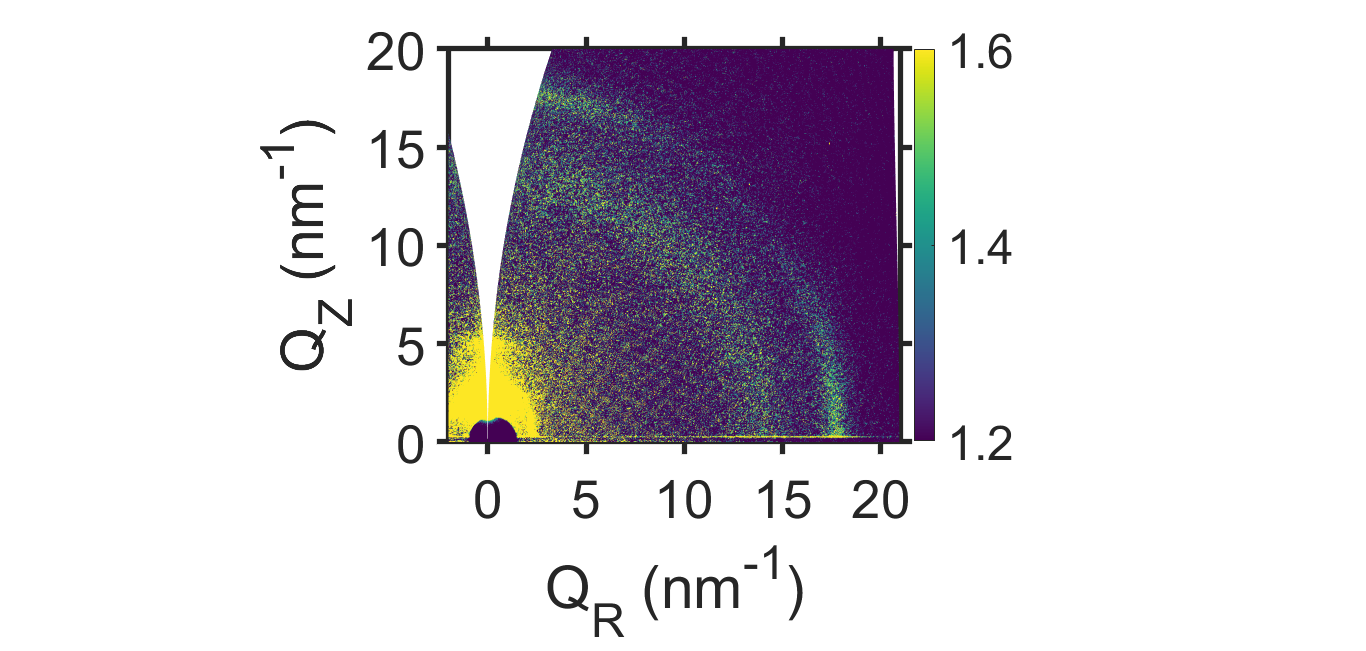


**as-cast**

**200°C**

**100°C**


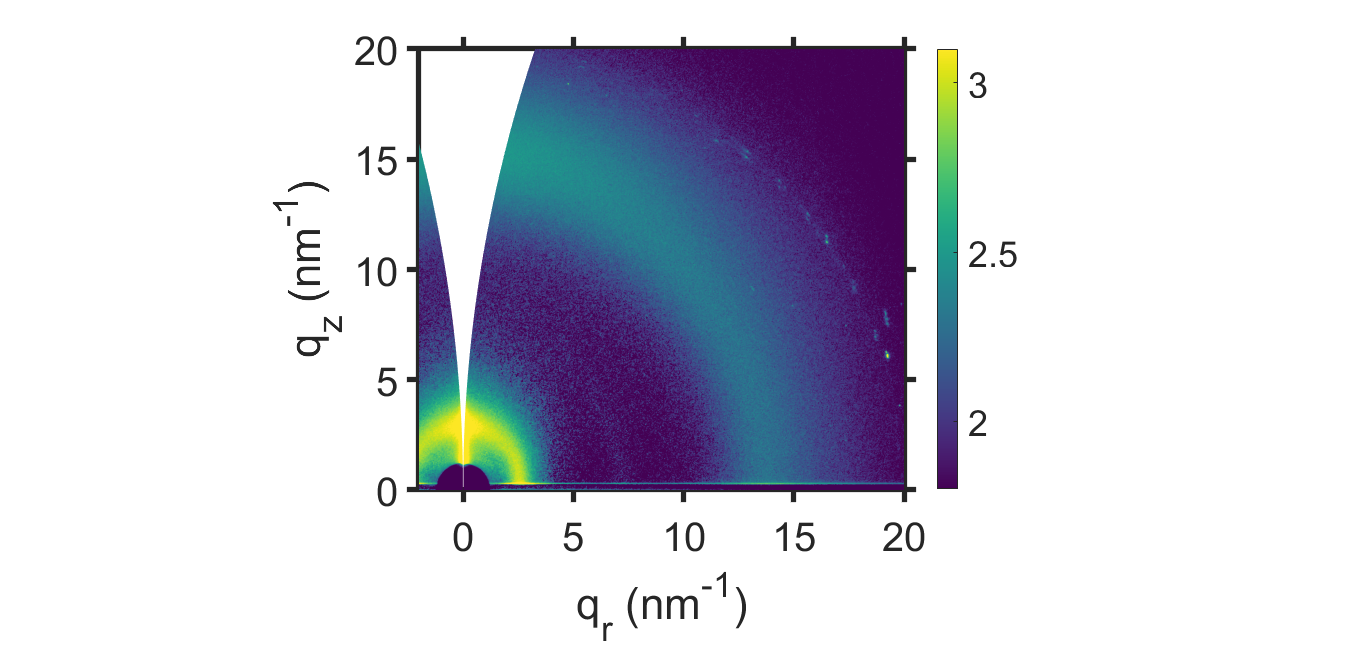

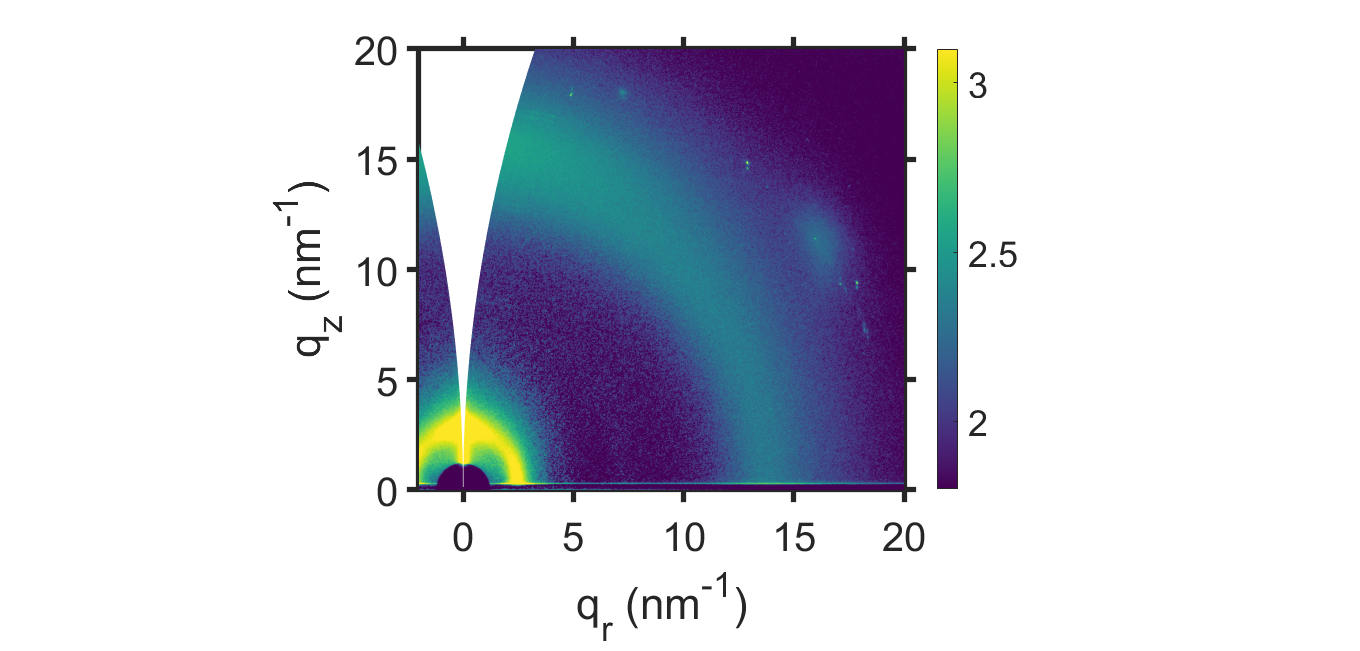


**as-cast**

**200°C**

**100°C**


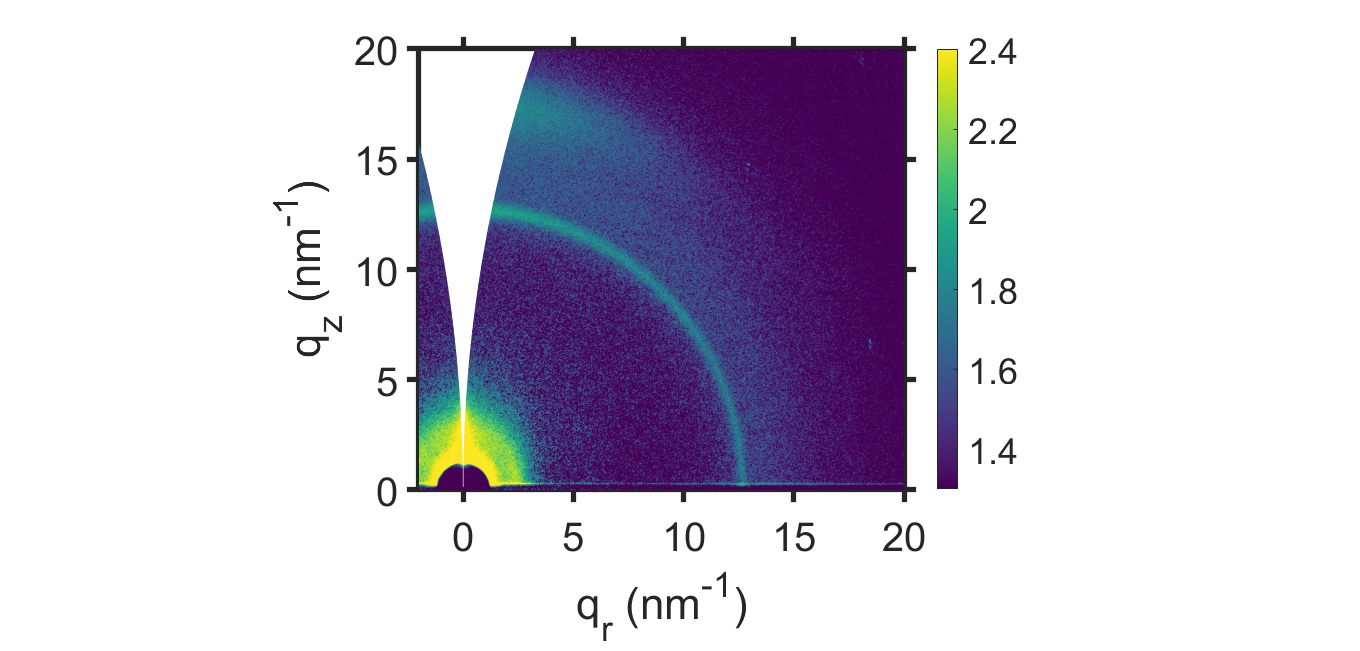

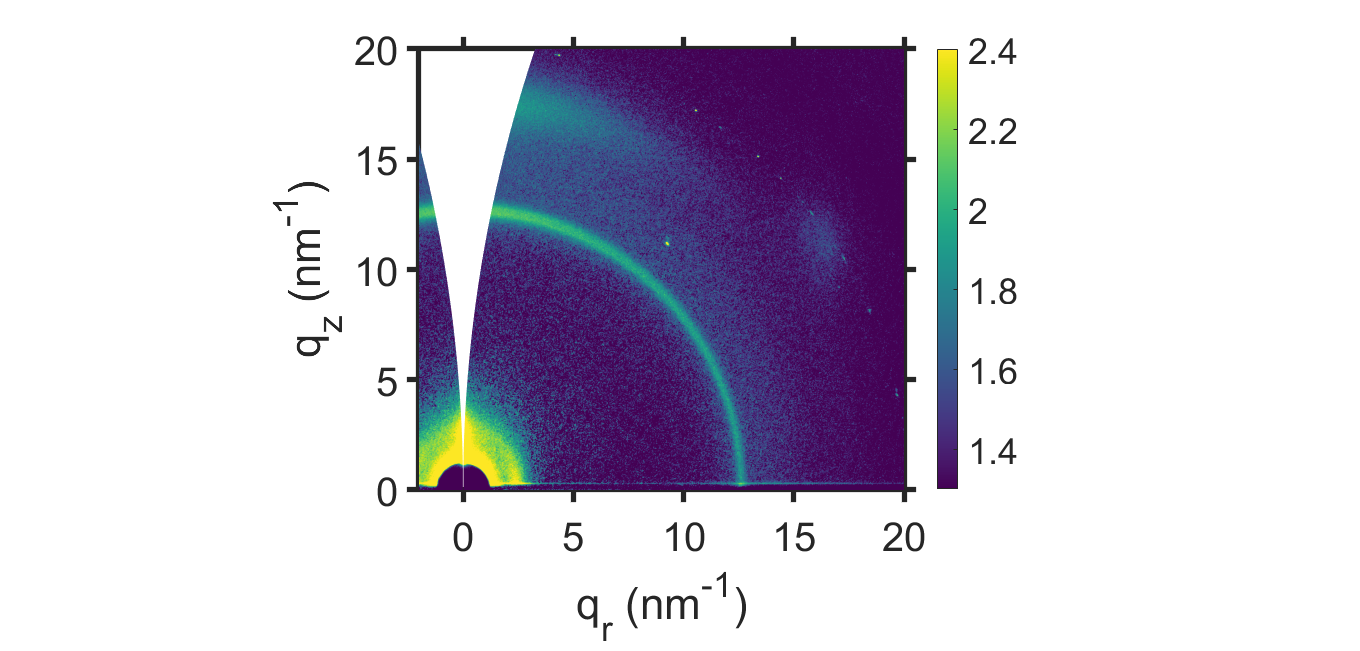


**as-cast**

**200°C**

**100°C**

**PNDI-T(SR)BTT**

**PNDIV-T(SR)BTT**


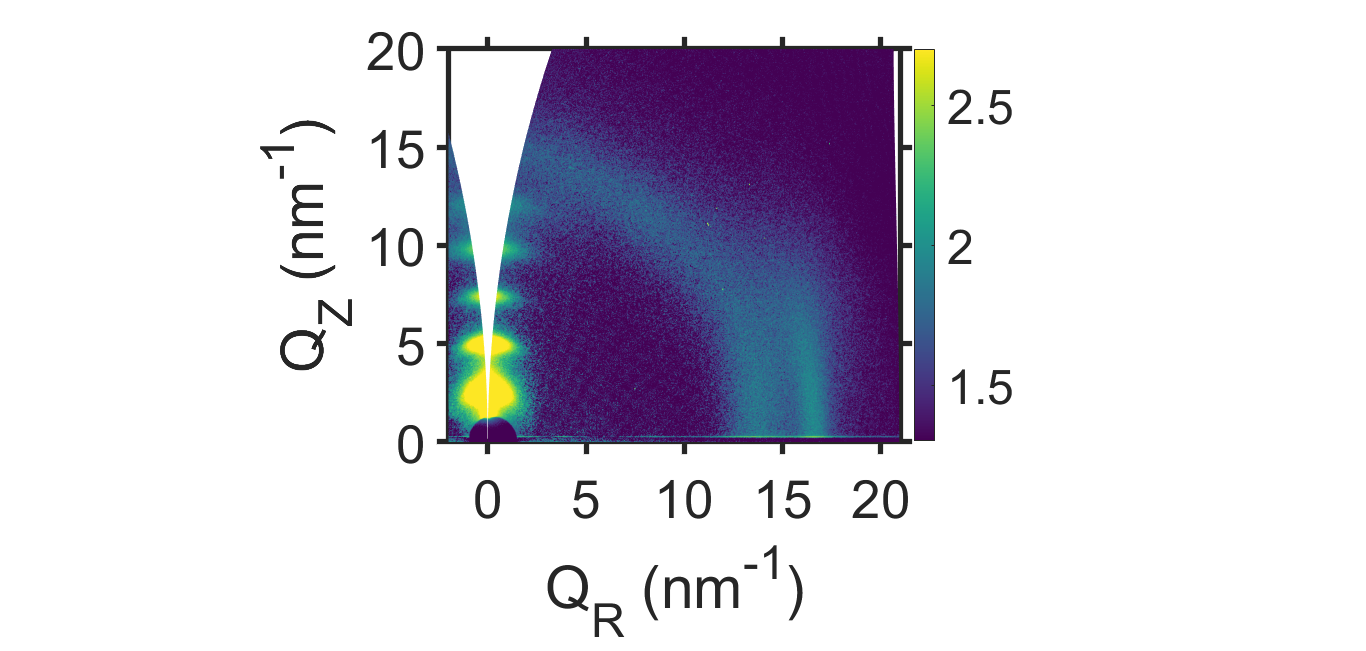


**Figure S13.** GIWAXS images of PNDI-TfBTT, PNDIV-TfBTT, PNDI-T(SR)BTT, and PNDIV-T(SR)BTT films as-cast and after annealing at 100 °C.


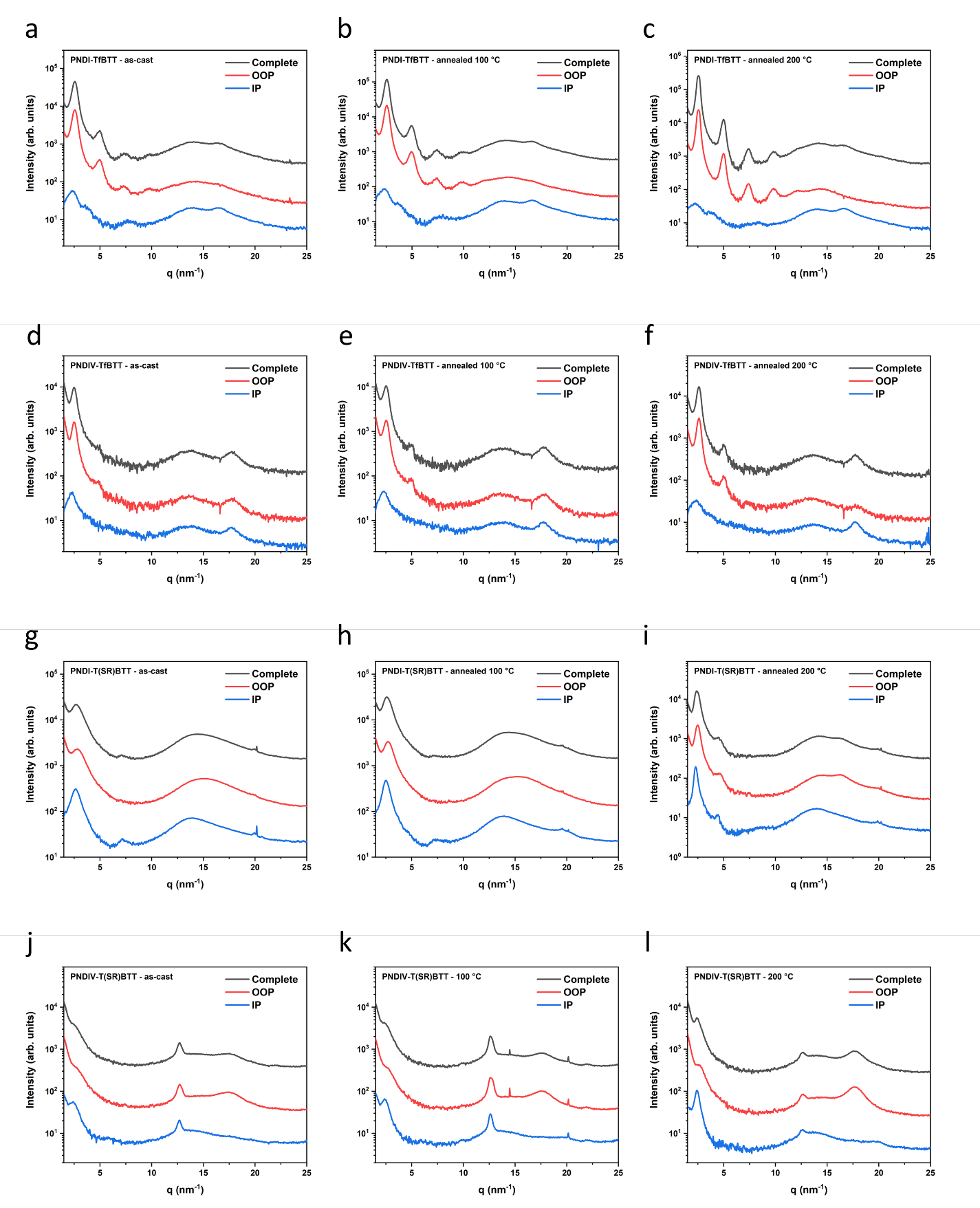


**Figure S14.** GIWAXS diffraction patterns (in-plane (IP), out-of-plane (OOP), and combined) for as-cast, annealed at 100 °C, and annealed at 200 °C, films of PNDI-TfBTT (a-c), PNDIV-TfBTT (d-f), PNDI-T(SR)BTT (g-i), and PNDIV-T(SR)BTT (j-l).

**Table S5.** Summary of key metrics obtained from the GIWAXS data of PNDI-TfBTT, PNDIV-TfBTT, PNDI-T(SR)BTT, and PNDIV-T(SR)BTT thin films annealed at 200 °C for 30 min.

| **Polymer** | **CCL (100) (nm)** | **CCL (010) (nm)** | **d-spacing (100) (Å)** | **d-spacing (010) (Å)** | **Orientation** |
| --- | --- | --- | --- | --- | --- |
| **PNDI-TfBTT** | 16.3 | 1.8 | 24.6 | 3.8 | Edge-on |
| **PNDIV-TfBTT** | 12.9 | 4.5 | 24.1 | 3.5 | Edge-on |
| **PNDI-T(SR)BTT** | 11.0 | 1.7 | 26.2 | 3.8 | Face-on + Edge-on |
| **PNDIV-T(SR)BTT** | 13.6 | 2.6 | 26.2 | 3.6 | Face-on + Edge-on |


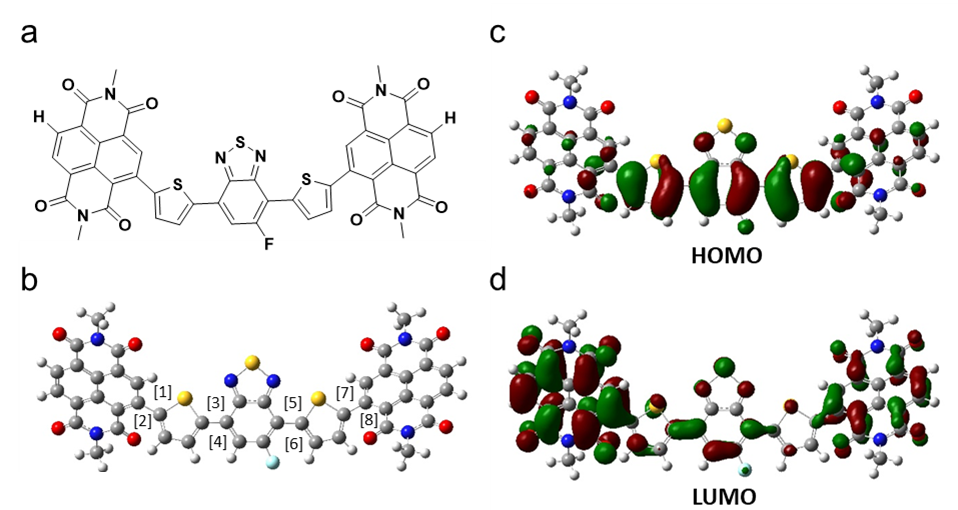


**Figure S15.** (a) Molecular structure used for DFT calculations. DFT optimisation of a model of PNDI-TfBTT at the B3LYP/6-31G(d,p) level showing (b) overall backbone geometry, (c) HOMO distribution along backbone, and (d) LUMO distribution along backbone.

**
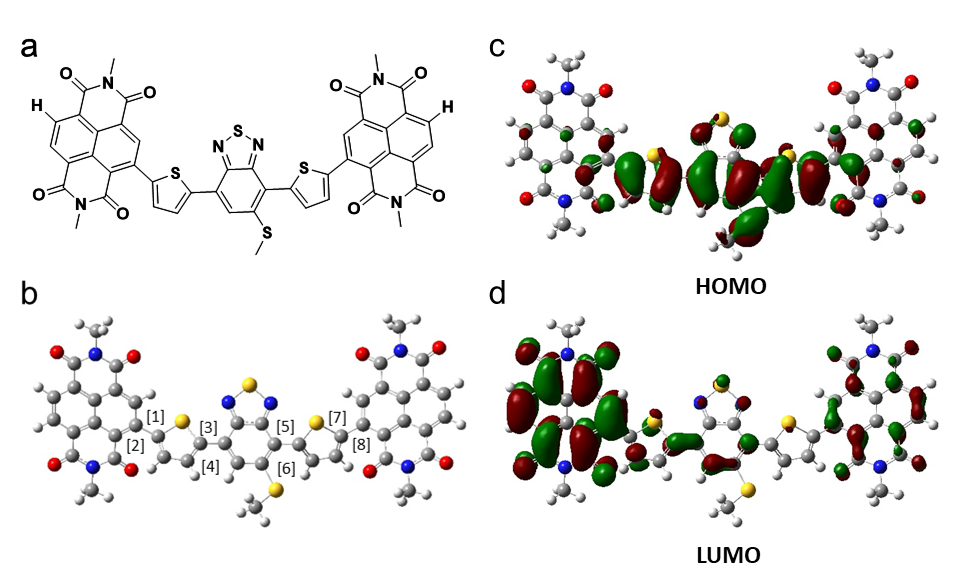
**

**Figure S16.** (a) Molecular structure used for DFT calculations. DFT optimisation of a model of PNDI-T(SR)BTT at the B3LYP/6-31G(d,p) level showing (b) overall backbone geometry, (c) HOMO distribution along backbone, and (d) LUMO distribution along backbone.

**
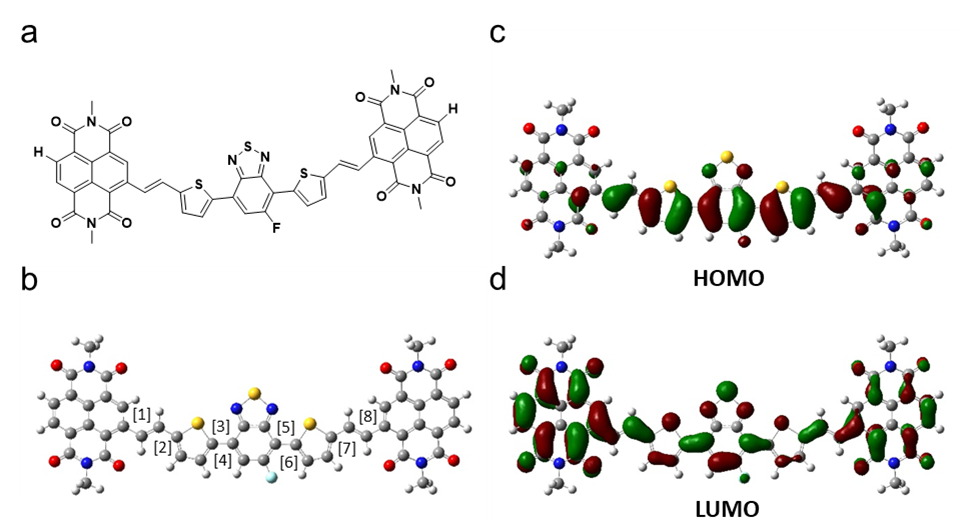
**

**Figure S17.** (a) Molecular structure used for DFT calculations. DFT optimisation of a model of PNDIV-TfBTT at the B3LYP/6-31G(d,p) level showing (b) overall backbone geometry, (c) HOMO distribution along backbone, and (d) LUMO distribution along backbone.


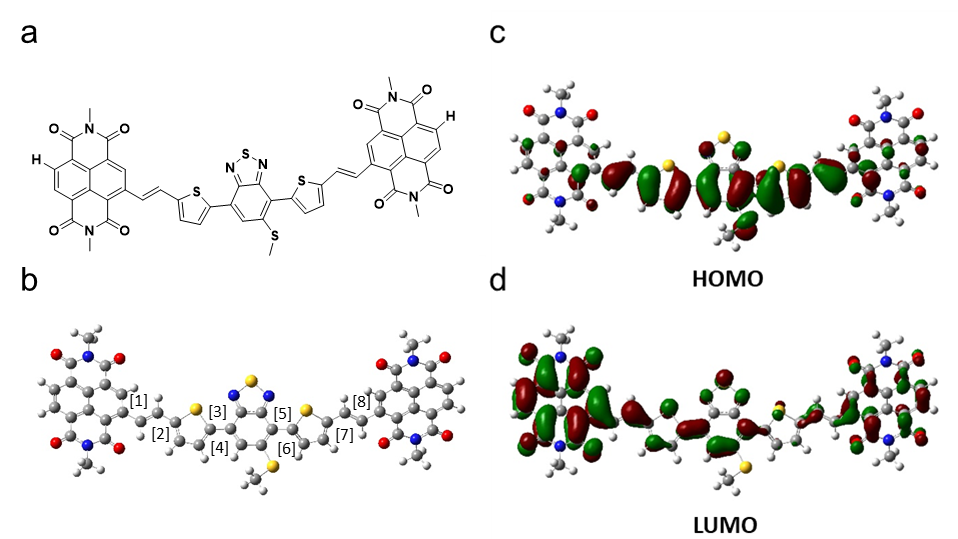


**Figure S18.** (a) Molecular structure used for DFT calculations. DFT optimisation of a model of PNDIV-T(SR)BTT at the B3LYP/6-31G(d,p) level showing (b) overall backbone geometry, (c) HOMO distribution along backbone, and (d) LUMO distribution along backbone.

**PNDI-TfBTT**

**PNDI-TfBTT**

**PNDI-T(SR)BTT**

**PNDI-T(SR)BTT**

**PNDIV-TfBTT**

**PNDIV-TfBTT**

**PNDIV-T(SR)BTT**

**PNDIV-T(SR)BTT**

**Figure S19.** PESA measurements for PNDI-TfBTT, PNDIV-TfBTT, PNDI-T(SR)BTT, and PNDIV-T(SR)BTT.


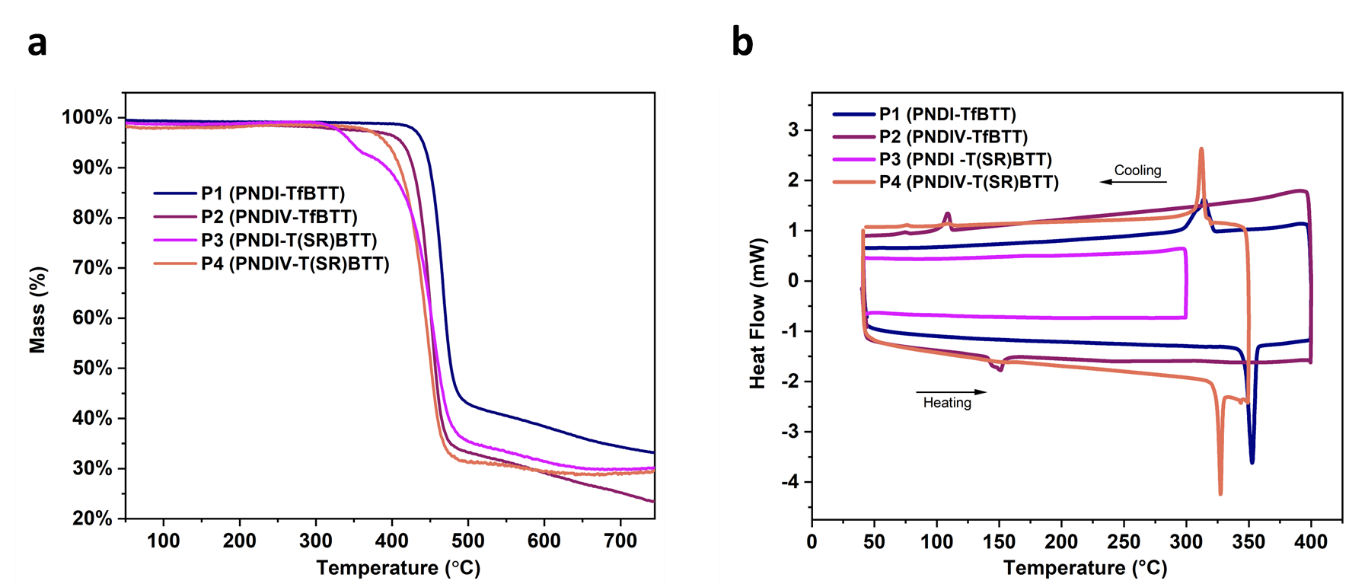


**Figure S20.** (a) TGA data for PNDI-TfBTT, PNDIV-TfBTT, PNDI-T(SR)BTT and PNDIV-T(SR)BTT carried out in a nitrogen atmosphere at 5 °C min^-1^. (b) DSC data for PNDI-TfBTT, PNDIV-TfBTT, PNDI-T(SR)BTT and PNDIV-T(SR)BTT carried out in a nitrogen atmosphere at 10 °C min^-1^.


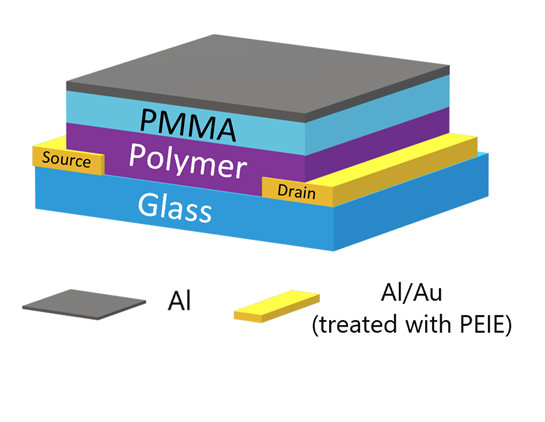


Glass

Polymer

**Figure S21.** OTFT device architecture.

**Table** **S6.** Summary of OTFT data for PNDI-TfBTT, PNDIV-TfBTT, PNDI-T(SR)BTT and PNDIV-T(SR)BTT with a (TG/BG) configuration.

| **Polymer** | **µ_e,av_**  **(cm^2^V^-1^s^-1^)** | **µ_e,max_**  **(cm^2^V^-1^s^-1^)** | **Temperature annealed (°C)** | **Optimum annealing temperature (°C)** |
| --- | --- | --- | --- | --- |
| **PNDI-TfBTT** | 1.4 x 10^-1^ | 1.6 x 10^-1^ | 200 | 180 - 200 |
| **PNDIV-TfBTT** | 3.7 x 10^-2^ | 4.7 x 10^-2^ | 220 | 220 |
| **PNDI-T(SR)BTT** | 1.3 x 10^-2^ | 1.5 x 10^-2^ | 250 | 250 |
| **PNDIV-T(SR)BTT** | 1.4 x 10^-2^ | 1.4 x 10^-2^ | 220 | 220 |

**Table S7.** GPC data for PNDI-T(SAz)BTT - 10%, and PNDI-T(SAz)BTT - 100% in chlorobenzene at 80 °C. Analysis was affected by aggregation observed in both polymers.

| **Polymer** | **M_n_ (kDa)** | **M_w_ (kDa)** | ***Đ*** |
| --- | --- | --- | --- |
| PNDI-T(SAz)BTT - 10% | 100 | 251 | 2.5 |
| PNDI-T(SAz)BTT - 100% | 23 | 66 | 2.8 |


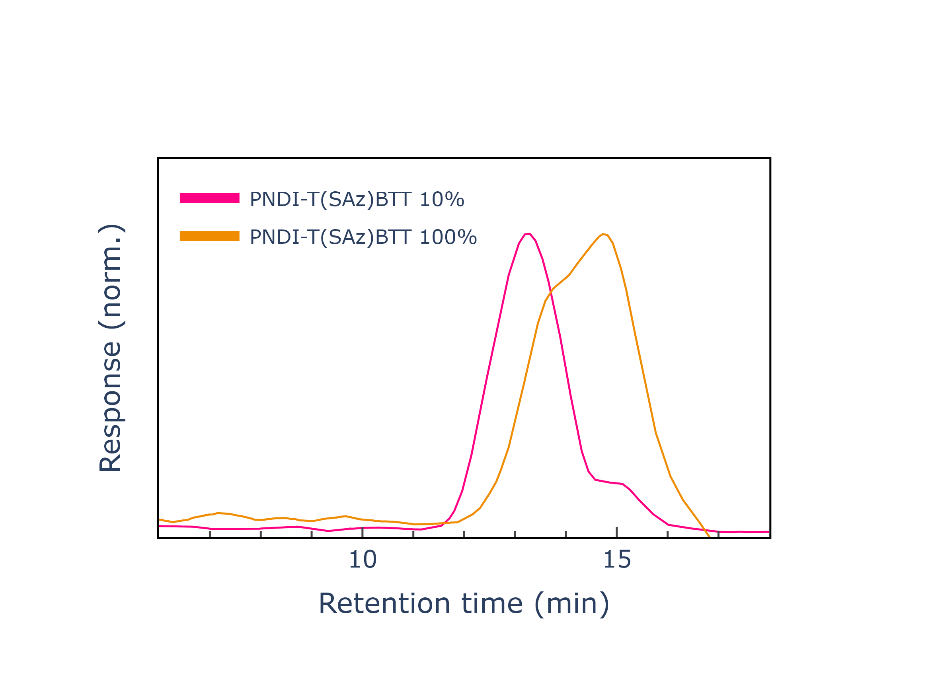


**Figure S22.** GPC traces for PNDI-T(SAz)BTT - 10%, and PNDI-T(SAz)BTT - 100% in chlorobenzene at 80 °C.


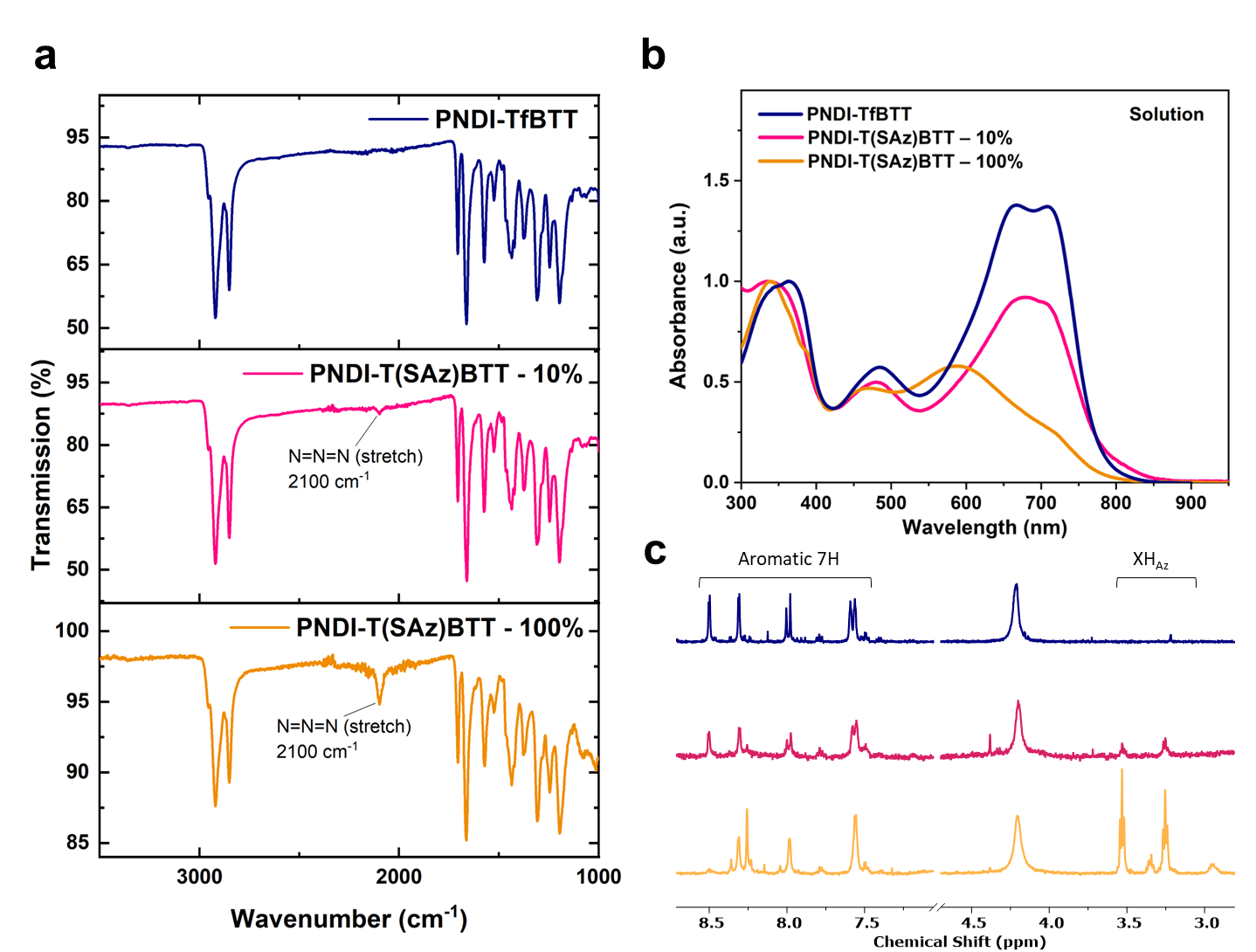


**Figure S23.** Substitution of 3-azidopropane-1-thiol onto the PNDI-TfBTT backbone with a low (10 mol%) and high (100 mol%) degree of substitution: (a) IR, (b) UV-Vis, and (c) ^1^H NMR spectra for increasing substitution along the PNDI-TfBTT backbone where the aromatic peaks from the aromatic protons were set to an integral value of 7H and directly compared with XHAz.

*
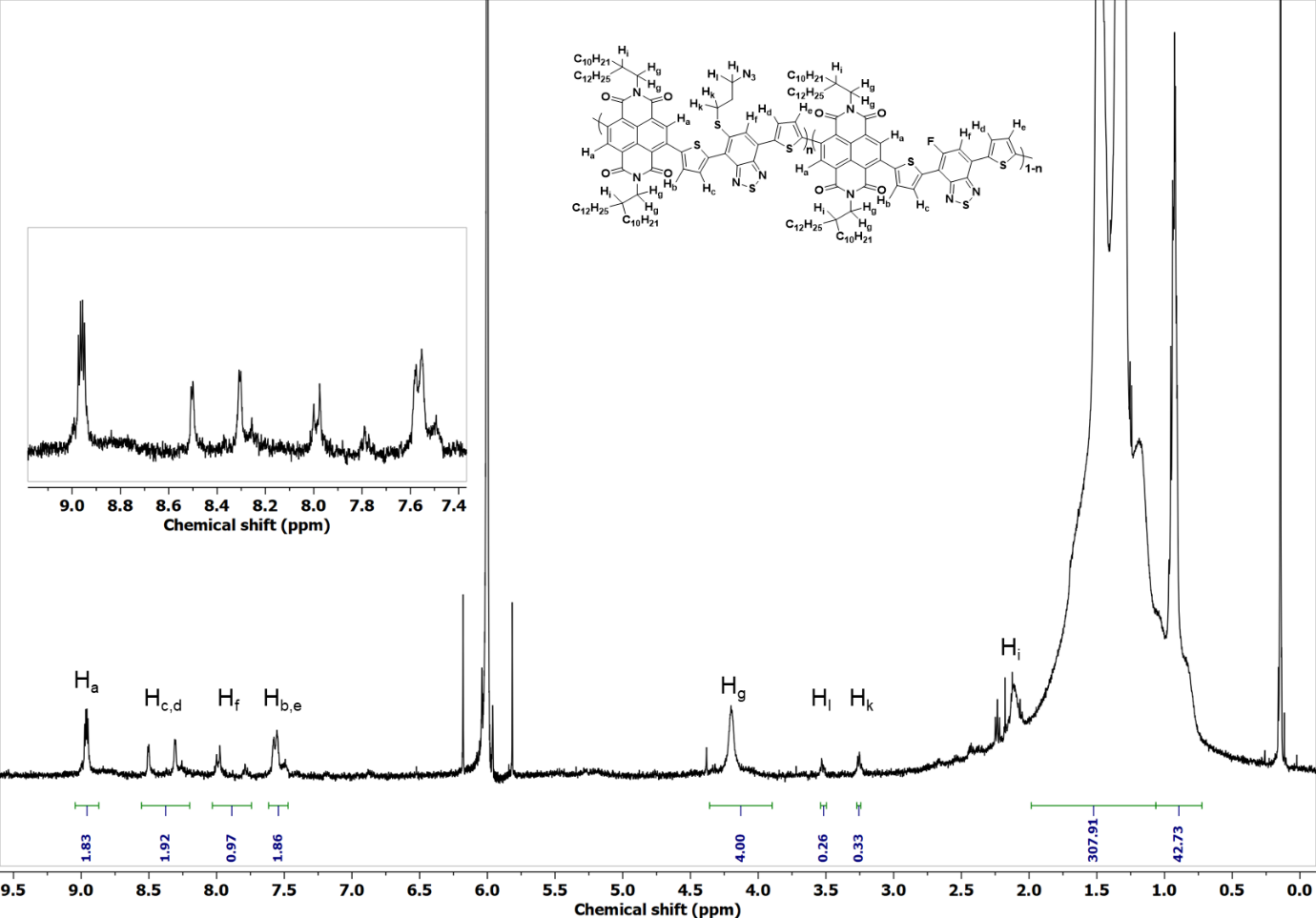
*

**Figure S24.** ^1^H NMR of PNDI-T(SAz)BTT-10% in TCE-d at 373K.


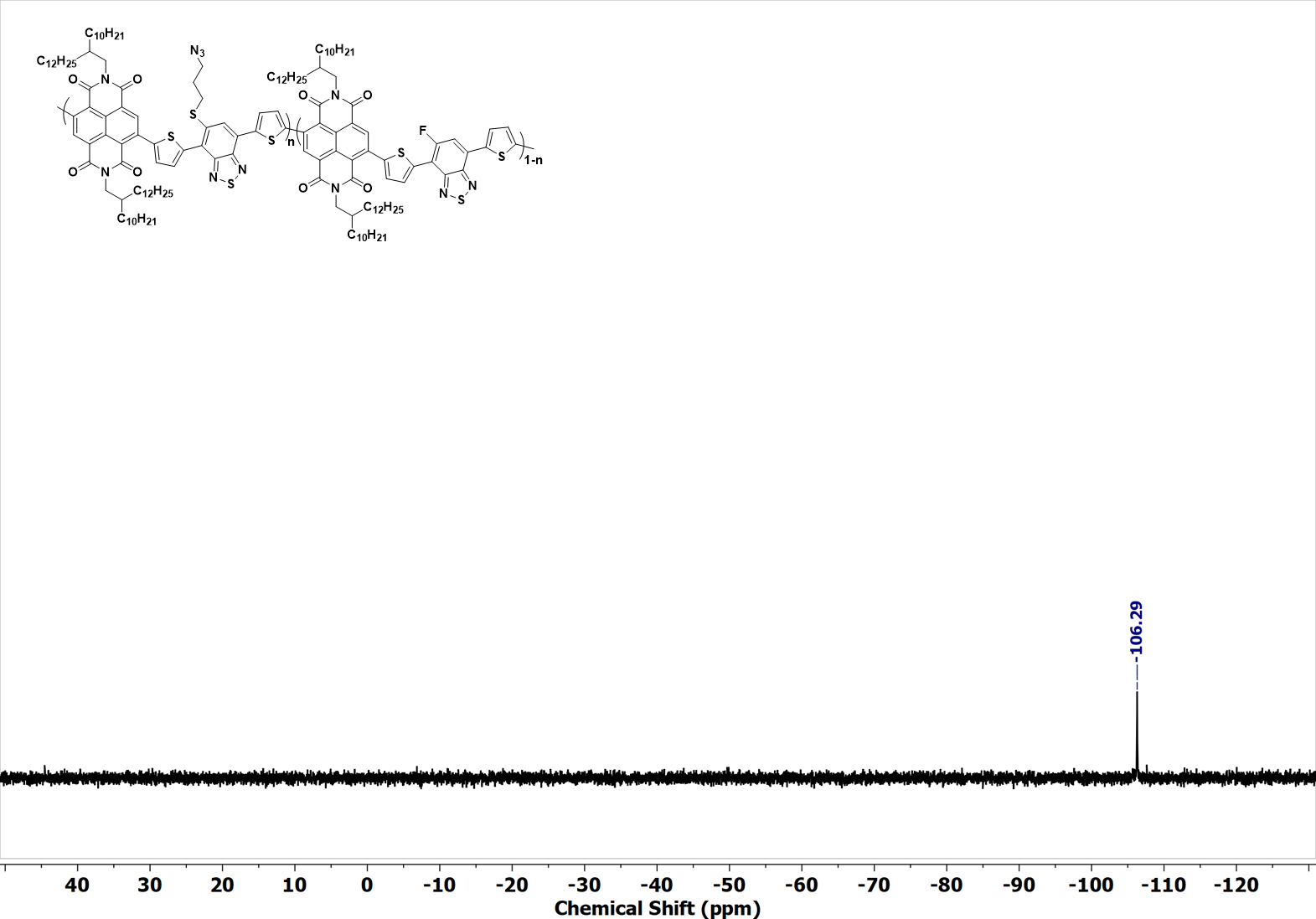


**Figure S25.** ^19^F NMR of PNDI-T(SAz)BTT-10% in TCE-d at 373 K. Baseline spline corrected.

**
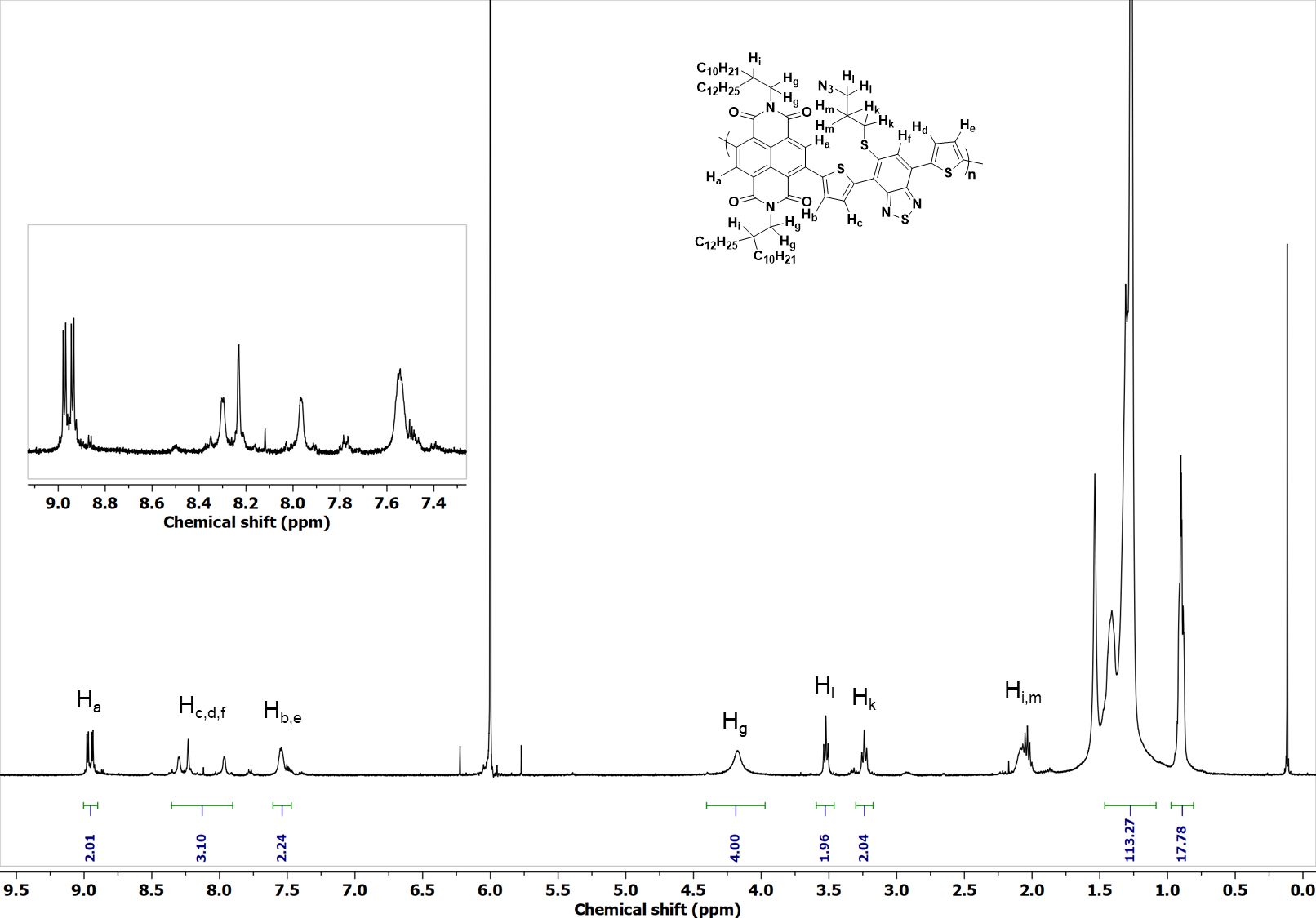
**

**Figure S26.** ^1^H NMR of PNDI-T(SAz)BTT-100% in TCE-d at 333 K..

**
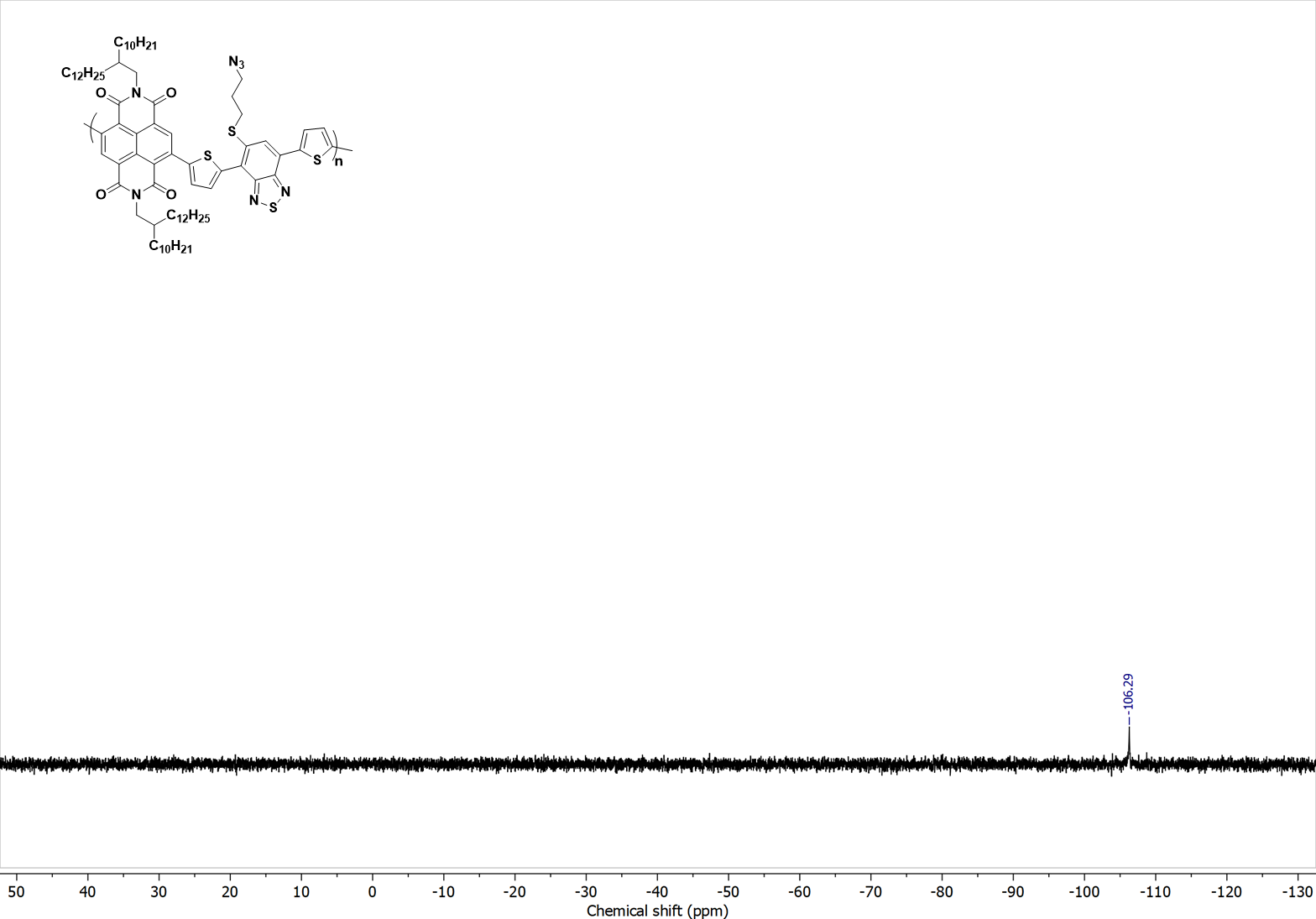
**

**Figure S27.** ^19^F NMR of PNDI-T(SAz)BTT-100% in TCE-d at 333 K. Baseline spline corrected.

**Table S8.** Calculated substitution percentage of the fluorine groups by SAz for PNDI-T(SAz)BTT 10% and 100%, as measured by ^1^H NMR.

| **Sample** | **H_g_** | **H_k_** | **H_l_** | **Mean of H_k_ and H_l_** | **Expected H_k_/H_l_ mean** | **Implied substitution**  **percentage (%)** |
| --- | --- | --- | --- | --- | --- | --- |
| PNDI-TfBTT 0% | 4.00 | 0.00 | 0.00 | 0.00 | 0.00 | 0 |
| PNDI-T(SAz)BTT 10% | 4.00 | 0.26 | 0.33 | 0.30 | 0.20 | 15 |
| PNDI-T(SAz)BTT 100% | 4.00 | 1.96 | 2.04 | 2.00 | 2.00 | 100 |


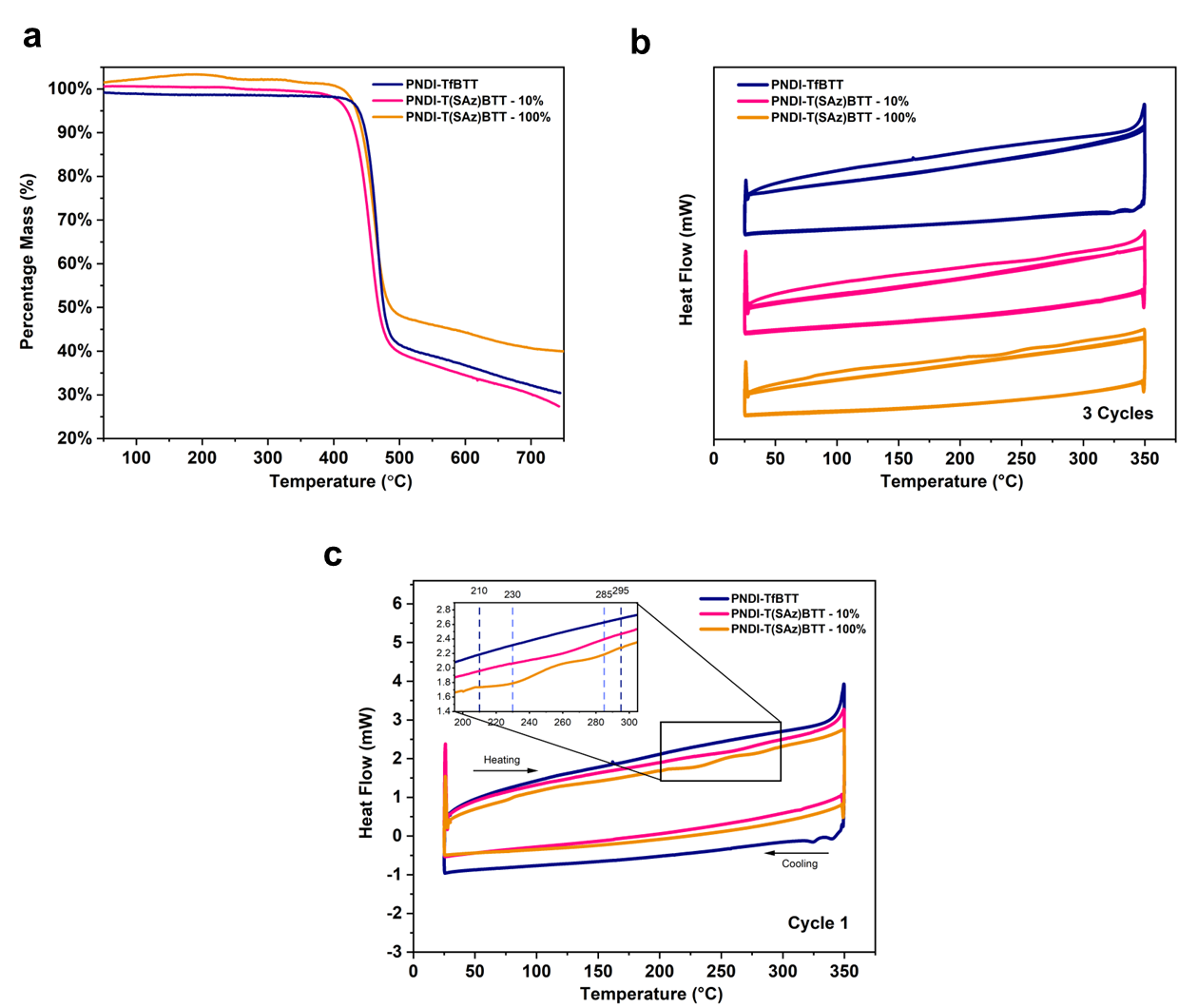


**Figure S28.** (a) TGA data for PNDI-TfBTT, PNDI-T(SAz)BTT-10% and PNDI-T(SAz)BTT-100% carried out in a nitrogen atmosphere at 5 °C min^-1^_._ (b,c) DSC data for PNDI-TfBTT, PNDI-T(SAz)BTT-10% and PNDI-T(SAz)BTT-100% carried out in a nitrogen atmosphere at 10 °C min^-1^.

**Figure S29.** GIWAXS diffraction patterns for PNDI-T(SAz)BTT-10% films as-cast and after annealing at 200 °C and 300 °C, with (254 nm) and without UV exposure prior to annealing.

**Figure S30.** GIWAXS diffraction patterns for PNDI-T(SAz)BTT-100% films as-cast, and after annealing at 200 °C, and 300 °C, with (254 nm) and without UV exposure prior to annealing.

**Table S9.** Summary of key metrics obtained from the GIWAXS data of PNDI-T(SAz)BTT-10%, and PNDI-T(SAz)BTT-100% thin films annealed at 200 °C for 30 min.

| **Polymer** | | **CCL (100) (nm)** | | **CCL (010) (nm)** | | **d-spacing (100) (Å)** | | **d-spacing (010) (Å)** | **Orientation** |
| --- | --- | --- | --- | --- | --- | --- | --- | --- | --- |
| **PNDI-T(SAz)BTT - 10%** | | 17.4 | | 2.5 | | 24.5 | | 3.8 | Edge-on |
| **PNDI-T(SAz)BTT - 100%** | | 8.5 | | 2.5 | | 23.9 | | 3.9 | Face-on and + Edge-on |
|  |  | |  | |  | |  | |  |


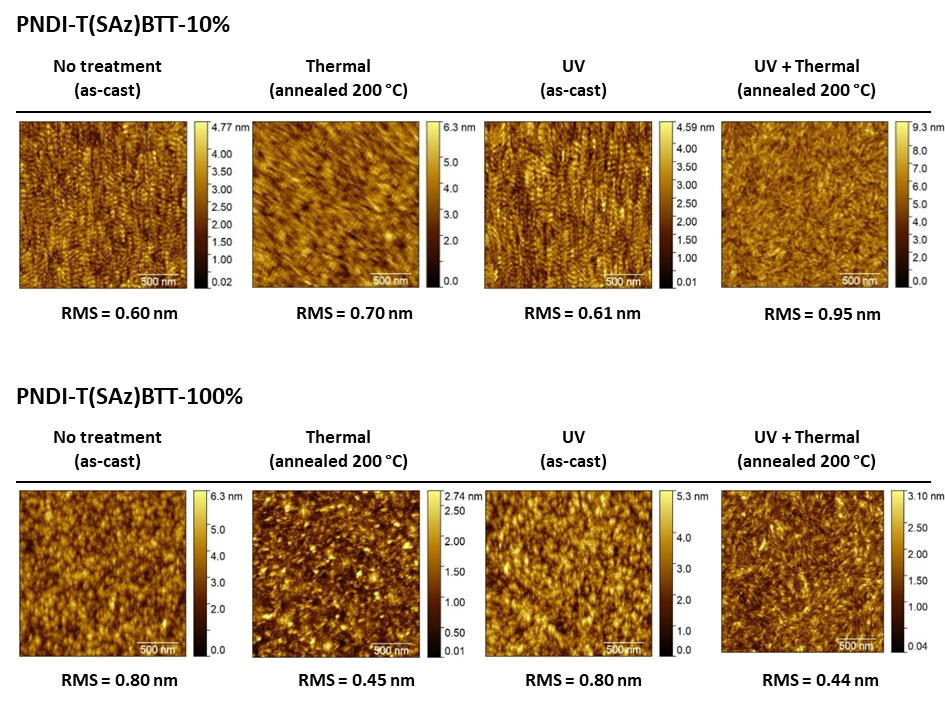


**Figure S31.** AFM images of PNDI-T(SAz)BTT-10% (top) and PNDI-T(SAz)BTT-100% (bottom) thin films, from spin casting on Si wafer. The films were imaged as-cast, after thermal annealing (at 200 °C), after UV light exposure (254 nm) and after both UV exposure (254 nm) followed by thermal annealing (at 200 °C).

**As-cast**

**As-cast**

**Annealed at 200 °C**

**Annealed at 300 °C**

**Figure S32.** Transfer and output characteristics of the PNDI-T(SAz)BTT-10% OTFT device.

**Annealed at 200 °C**

**Annealed at 300 °C**

**As-cast**

**As-cast**

**Figure S33.** Transfer and output characteristics of the PNDI-T(SAz)BTT-100% OTFT device.

**UV - 200 °C**

**UV - 300 °C**

**UV - As-cast**

**Figure S35.** Transfer characteristics of the PNDI-T(SAz)BTT-100% OTFT device, where the thin films were exposed to UV light.

**UV - 200 °C**

**UV - 300 °C**

**UV - As-cast**

**Figure S34.** Transfer characteristics of the PNDI-T(SAz)BTT-10% OTFT device, where thin films were exposed to UV light.

References

(1) Letizia, J. A.; Salata, M. R.; Tribout, C. M.; Facchetti, A.; Ratner, M. A.; Marks, T. J. N-Channel Polymers by Design: Optimizing the Interplay of Solubilizing Substituents, Crystal Packing, and Field-Effect Transistor Characteristics in Polymeric Bithiophene-Imide Semiconductors. *J. Am. Chem. Soc.* **2008**, *130* (30), 9679–9694. https://doi.org/10.1021/ja710815a.

(2) Guo, X.; Watson, M. D. Conjugated Polymers from Naphthalene Bisimide. *Org. Lett.* **2008**, *10* (23), 5333–5336. https://doi.org/10.1021/ol801918y.

(3) Chen, Z.; Zheng, Y.; Yan, H.; Facchetti, A. Naphthalenedicarboximide- vs Perylenedicarboximide-Based Copolymers. Synthesis and Semiconducting Properties in Bottom-Gate N-Channel Organic Transistors. *J. Am. Chem. Soc.* **2009**, *131* (1), 8–9. https://doi.org/10.1021/ja805407g.

(4) Fei, Z.; Han, Y.; Martin, J.; Scholes, F. H.; Al-Hashimi, M.; Alqaradawi, S. Y.; Stingelin, N.; Anthopoulos, T. D.; Heeney, M. Conjugated Copolymers of Vinylene Flanked Naphthalene Diimide. *Macromolecules* **2016**, *49* (17), 6384–6393. https://doi.org/10.1021/acs.macromol.6b01423.

(5) Zhao, Z.; Yin, Z.; Chen, H.; Zheng, L.; Zhu, C.; Zhang, L.; Tan, S.; Wang, H.; Guo, Y.; Tang, Q.; Liu, Y. High-Performance, Air-Stable Field-Effect Transistors Based on Heteroatom-Substituted Naphthalenediimide-Benzothiadiazole Copolymers Exhibiting Ultrahigh Electron Mobility up to 8.5 Cm V−1 S−1. *Adv. Mater.* **2017**, *29*, 1602410. https://doi.org/10.1002/adma.201602410.
